# Estimating the burden of antimicrobial resistance: a systematic literature review

**DOI:** 10.1186/s13756-018-0336-y

**Published:** 2018-04-25

**Authors:** Nichola R. Naylor, Rifat Atun, Nina Zhu, Kavian Kulasabanathan, Sachin Silva, Anuja Chatterjee, Gwenan M. Knight, Julie V. Robotham

**Affiliations:** 1 National Institute for Health Research Health Protection Research Unit in Healthcare Associated Infection and Antimicrobial Resistance at Imperial College, Hammersmith Campus, London, W12 0NN UK; 2 Imperial College London, Sir Alexander Fleming Building, South Kensington Campus, London, UK; 3 Harvard University, 665 Huntington Avenue, Boston, MA 02115 USA; 4 Modelling and Economics Unit, National Infection Service, Public Health England, 61 Colindale Avenue, London, NW9 5EQ UK

**Keywords:** Antimicrobial resistance, Antibiotic resistance, Burden, Cost

## Abstract

**Background:**

Accurate estimates of the burden of antimicrobial resistance (AMR) are needed to establish the magnitude of this global threat in terms of both health and cost, and to paramaterise cost-effectiveness evaluations of interventions aiming to tackle the problem. This review aimed to establish the alternative methodologies used in estimating AMR burden in order to appraise the current evidence base.

**Methods:**

MEDLINE, EMBASE, Scopus, EconLit, PubMed and grey literature were searched. English language studies evaluating the impact of AMR (from any microbe) on patient, payer/provider and economic burden published between January 2013 and December 2015 were included. Independent screening of title/abstracts followed by full texts was performed using pre-specified criteria. A study quality score (from zero to one) was derived using Newcastle-Ottawa and Philips checklists. Extracted study data were used to compare study method and resulting burden estimate, according to perspective. Monetary costs were converted into 2013 USD.

**Results:**

Out of 5187 unique retrievals, 214 studies were included. One hundred eighty-seven studies estimated patient health, 75 studies estimated payer/provider and 11 studies estimated economic burden. 64% of included studies were single centre. The majority of studies estimating patient or provider/payer burden used regression techniques. 48% of studies estimating mortality burden found a significant impact from resistance, excess healthcare system costs ranged from non-significance to $1 billion per year, whilst economic burden ranged from $21,832 per case to over $3 trillion in GDP loss. Median quality scores (interquartile range) for patient, payer/provider and economic burden studies were 0.67 (0.56-0.67), 0.56 (0.46-0.67) and 0.53 (0.44-0.60) respectively.

**Conclusions:**

This study highlights what methodological assumptions and biases can occur dependent on chosen outcome and perspective. Currently, there is considerable variability in burden estimates, which can lead in-turn to inaccurate intervention evaluations and poor policy/investment decisions. Future research should utilise the recommendations presented in this review.

**Trial registration:**

This systematic review is registered with PROSPERO (PROSPERO CRD42016037510).

**Electronic supplementary material:**

The online version of this article (10.1186/s13756-018-0336-y) contains supplementary material, which is available to authorized users.

## Background

Antimicrobial resistance (AMR) is a cause for global concern due to the current and potential impact on global population health, costs to healthcare systems and Gross Domestic Product (GDP), mainly through reduced treatment options [1]. Recent reports suggest that absolute numbers of infections due to resistant microbes are increasing globally [2–4]. Estimates of the potential economic burden of AMR from recent reports, such as ‘The Review on Antimicrobial Resistance’ [1], are being utilised by policy makers to push AMR up the political agenda [5]. However, more precise estimates of AMR burden are needed to inform policy through health economic models evaluating interventions attempting to prevent, treat or stop the spread of resistant infections [6, 7].

In order to establish burden, the perspective being taken should be defined. The patient perspective refers to associated mortality and morbidity (including clinical outcomes), while the payer perspective can include attributable costs to the payers of healthcare including insurers or national payers [6]. The provider perspective estimates the burden to certain providers of healthcare, such as hospitals and primary care practices [6]. In cases where there is national/governmental provision and payment of healthcare services, the provider and payer perspectives may align to be the same, such as in the case of the National Health Service and the Department of Health in England [6]. The economic (or societal) perspective generally includes the potential impact on the labour force through lost productivity [8, 9], but can also include the burden on carers and patient out-of-pocket expenses [10, 11]. AMR may also create burden through secondary effects, referred to in this review as secondary burden. Secondary patient burden, and onward effects to healthcare system or society, occurs when procedures that utilise antimicrobials to reduce the risk of post-intervention adverse events (such as surgical procedures utilising prophylactic antibiotics) are performed less frequently due to AMR increasing the risk of adverse events [12].

The different perspectives of burden produce varying outcome measures. For example, from a patient perspective an excess mortality figure may be determined [13], whilst looking from a payer or provider perspective could produce an excess cost of hospital treatment in monetary terms [14], and economic burden could refer to excess GDP losses for a country [15]. Obtaining estimates for these different outcomes, may require different methodologies, ranging from case-control studies with regression analyses to complex mathematical and economic models [16, 17].

Past descriptive review articles have discussed the methods of estimating the burden of AMR [9, 18, 19]. However, since an update in 2012 of a previous systematic review [8, 20], there has been no formal systematic assessment of the methods used in AMR burden estimation, or the variation in resulting outcomes. The 2012 update concluded economic evidence was lacking, and that over the 21 studies included there were large variations in the estimates of AMR burden [8]. However, there been no formal quality assessment of recent AMR burden evidence across the stated perspectives, which is needed when discussing study methodology and its limitations. Given the growing media coverage of and policy interest in AMR over recent years [21, 22], there has been a corresponding marked increase in the published evidence on AMR impact. As such, this review aimed to capture and assess this recent portion of the literature, by reviewing the evidence published since the review in 2012 by Smith and Coast [8]. This review aimed to capture both the burden in the sense of the incremental impact of resistance (in addition to infection) and the burden of resistant infections among all of the population. Therefore this systematic review aimed to address the following research questions in regards to the human population, with no limitation on microbe of interest; (i) what perspectives and resulting methodologies have been used to estimate AMR burden in the recent published literature? (ii) Do AMR burden estimates differ by perspective and methodology? (iii) What is the quality of this recent evidence on the burden of AMR?

## Methods

This Systematic review is in line with PRISMA guidance, is registered with PROSPERO (registration number PROSPERO CRD42016037510) and has a previously published protocol [23–25].

### Search strategy and eligibility criteria

Studies which aimed to quantify the burden of AMR (in humans) published since the Smith and Coast review in 2012 [8] were of interest, and as such, the search was limited from January 2013 – December 2015. Ovid ‘Medline & EMBASE’, Scopus and EconLit were searched utilising a search string, which employed combinations of the following terms; excess, associated, attributable, burden, morbidity, mortality, cost, economic, clinical, global, impact, outcome, burden, antibiotic, antimicrobial, multi-drug, microbial-drug, resistan*, gram-positive, gram-negative, susceptib*,nonsusceptib*, enterococc*, Escherichia, streptococc*, staphylococc*, klebsiealla, pseudomonas, neisseria, chlamydia, clostridi*, mycobacteri*. In deviation to the published protocol [25], an additional search of PubMed was conducted for literature published within the stated time period, whereby titles were searched using the following string; “(((((mortality[Title]) OR cost[Title]) OR length of stay[Title]) OR productivity[Title]) AND resistant[Title]) AND infection*[Title]” [26]. The following websites were also searched for grey literature: Public Health England, Public Health Wales, Health Protection Scotland, NHS Health Scotland, Department of Health (UK), Health Protection Agency, National Institute for Health and Care Excellence, Centers for Disease Control and Prevention, World Health Organisation, European Commission for Public Health, Review on Antimicrobial Resistance.

The modified PICO inclusion/exclusion criteria (Table 1) were utilised to screen title/abstracts and subsequently full-texts [27].

**Table 1:** Inclusion/Exclusion Criteria Applied [25]

Criteria	Inclusion	Exclusion
Population	Humans	Animals only
	All ages	Plants only
	All sexes	
	Infection with antimicrobial resistant organism (or similar such as Extended Spectrum Beta-lactamase producing organisms). This includes future predictions of related infected populations, such as in the case of a “post-antibiotic” era	
Outcomes	Associated health burden, including mortality and morbidity	Health-Related Quality of Life only
	Associated healthcare cost burden, including resource use and opportunity cost	Molecular biology only
	Economic burden, including loss of productivity	Epidemiology only
	Burden from not being able to use antibiotics in ways previously or currently used in healthcare, including reduced surgery or chemotherapy	Outcomes associated with the evaluation of an intervention only
Study design	Case–control studies	Editorials
	Cohort studies	Letters
	Cross–sectional studies	Case series reports
	Longitudinal studies	Conference reports
	Randomised controlled trials	Evaluations of interventions
	Modelling studies	Reviews
	Economic Evaluations	

### Data extraction and quality assessment

Data were extracted from each study into a Data Extraction Table using Microsoft Excel. The following variables were collected as part of this process; study identifier, perspective, infection of interest (exposure, non-exposure, case and control definitions), outcome, country setting, study population, study design, data setting, epidemiological scope, method, sample size, (resistance-related) result, stated limitations and quality (risk of bias).

To establish what perspectives and resulting methodologies have been used in establishing AMR burden, study perspectives were grouped into either patient, healthcare system (representing payer and/or provider perspectives) or economic/societal burden. Studies estimating the secondary effects of AMR can do so by estimating the potential impact on health (such as excess deaths), healthcare system or economic burden (excess cost), so could be marked under each of these perspectives. Study methodologies were grouped into the following categories; regression analysis (parametric regression models), survival analysis (this included semi-parametric Cox proportional hazard models and non-parametric time to event analysis), matching, multistate models (including decision trees), economic models (including total factor or computable general equilibrium models), stepwise calculations (for example synthesising evidence from the literature and applying simple calculations to arrive at an estimate), significance tests and other (if none of the former categories applied).

The outcome of particular interest for patient burden was mortality, using odds ratio (OR) and hazard ratio (HR) measures. In this review, length of stay (LoS), alongside monetary cost, was recorded as a healthcare system burden outcome, as it has been shown that for healthcare associated infections LoS is a major contributing factor to hospital costs [28]. For economic burden, monetary cost was considered the main outcome of interest, however reporting of productivity, GDP and other such economic measures were also recorded. Impact significance was defined by *p* values (less than 0.05) and 95% confidence intervals (CIs), where appropriate.

Risk of bias within individual studies was assessed using the Newcastle-Ottawa scales for cohort and case-control studies [29]. For this review a case-control study was defined when outcomes of interest (such death) were used to select the case and control groups, whereas cohort studies selected cases based on exposure to resistance [30]. The Newcastle-Ottawa checklists include four domains of quality and eight possible stars [29]. The Philips checklist was used for modelling studies, which includes three domains of quality and 57 possible stars [31]. A quality score was derived as the proportion of applicable checklist stars achieved (zero and one representing no checklist items achieved and all checklist items achieved respectively), please see Additional file 1: Tables S1, S2 and S3 for the full checklists utilised.

Risk of bias across the evidence was presented using the median and interquartile ranges of the quality score by perspective. This was a deviation in methodology from the published protocol, which proposed evaluating sign and significance of results [25], and was chosen as any other summary figures would be flawed due to the high heterogeneity in infection of interest and outcome [32].

### Data analysis

Due to study heterogeneity no meta-analyses were performed. Monetary costs found were converted into 2013 United States dollars (USD) by inflating the cost to 2013 original-currency estimates using annual inflation rates [33, 34], then converting this into USD utilising 2013 average exchange rates [35].

Descriptive statistics (such as proportions of papers and average quality scores), tables and graphs to collate and present ORs, HRs, LoS and monetary cost were executed in Microsoft Excel and R version 3.3.2 using packages ‘plyr’, ‘metafor’ and ‘ggplot2’ [36–38].

## Results

**Fig. 1:**
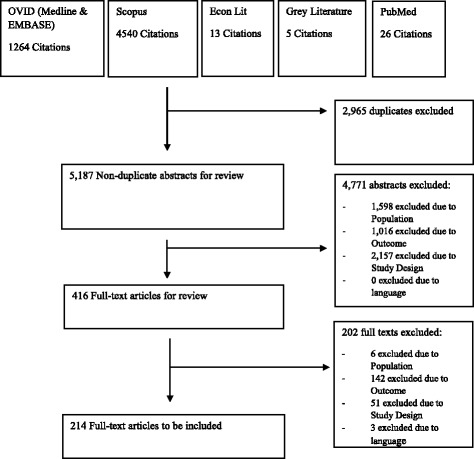
PRISMA Diagram of Article Retrieval & Inclusion

A total of 5187 unique titles and abstracts were retrieved over the specified search period. Applying the selection process resulted in a total of 214 studies being included in the final review (Fig. 1). One hundred eighty-seven studies included a patient burden measure, 75 a healthcare system burden measure, and only 11 studies included an economic burden measure (Table 2). The most individually studied genus was *Staphylococcus* (23%, 50/214), followed by *Klebsiella* (9%, 19/214) and *Acinetobacter* (8%, 18/214) respectively. The countries which individually produced the highest number of studies were the USA (19%, 40/214), followed by South Korea (7%, 15/214) and Spain (6%, 13/214). 64% of studies (138/214) used data from just one centre. The majority of studies (89%, 190/214) did not specify the epidemiological scope in estimating AMR burden, for example by stating whether relevant included infections were due to endemic- or outbreak-related microbes. See Additional file 1: Table S4 for individual study details.

**Table 2:** Perspectives & Methods used to Estimate the Burden of Antimicrobial Resistance

	Patient % (*n* = 187)	Healthcare System % (*n* = 75)	Economic % (*n* = 11)
Regression Analysis	44.9%	34.7%	9.1%
Survival Analysis	20.9%	9.3%	0.0%
Matching	4.8%	10.7%	9.1%
Multistate model	2.1%	6.7%	27.3%
Economic Model	0.0%	0.0%	18.2%
Significance Tests	26.2%	32.0%	0.0%
Stepwise calculation	1.1%	6.7%	27.3%
Qualitative	0.0%	0.0%	9.1%

Note that some studies included more than one burden perspective per study (for example a study reporting impact on mortality and costs would appear in multiple perspective categories)

### Estimating the patient burden of AMR

For estimating the patient burden of AMR, regression analyses and significance tests were the most utilised methods (Table 2). 95% (177/187) of studies estimating patient burden calculated mortality burden, with the remaining 5% (10/187) focusing on morbidity burden. Of those estimating a mortality burden, 48% (85/177) of studies found that AMR had a significant impact on mortality. Studies which aimed to estimate the impact of resistance on morbidity mainly focused on clinical outcomes such as clinical failure, time to stability, recurring infections or development of secondary infections [39–47].

**Fig. 2:**
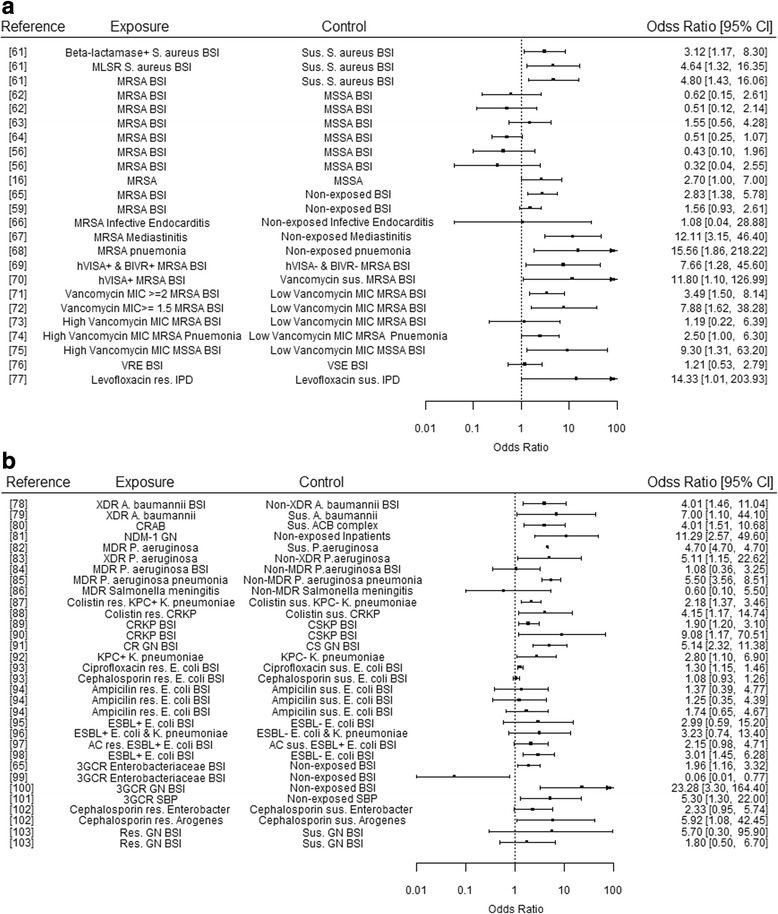
Odds ratios of Mortality Outcomes for Resistant Infections. Results presented are from studies utilising regression techniques, where 1.0 represents the point at which exposure does not affect the odds of the outcome occurring. The box point represents the reported OR value, with horizontal lines representing the reported 95% Confidence Interval. Results have not been adjusted or adapted to represent sample size, and are presented grouped by genera. **a** Gram-positive Bacteria. **b** Gram-negative Bacteria. [16, 56, 59, 61–103]

The majority of studies that utilised standard regression techniques to estimate ORs of a mortality event found resistance to be associated with higher mortality (Fig. 2a and Fig. 2b). Focusing on those which directly compared resistant and susceptible infections explicitly, 50% (7/14) and 47% (9/19) of the studies for Gram-positive and Gram-negative exposures, respectively, had 95% confidence intervals which crossed this OR = 1 threshold. This suggests non-statistical significance of such results. Across Gram-positive and Gram-negative infections, this occurred in 4/15 studies investigating resistant cases against “non-exposed” controls and in 2/8 studies investigating the burden of additional resistance mechanisms on already resistant infections (for example the impact of vancomycin resistance on Methicillin resistant *Staphylococcus aureus* (MRSA) patients).

**Fig. 3:**
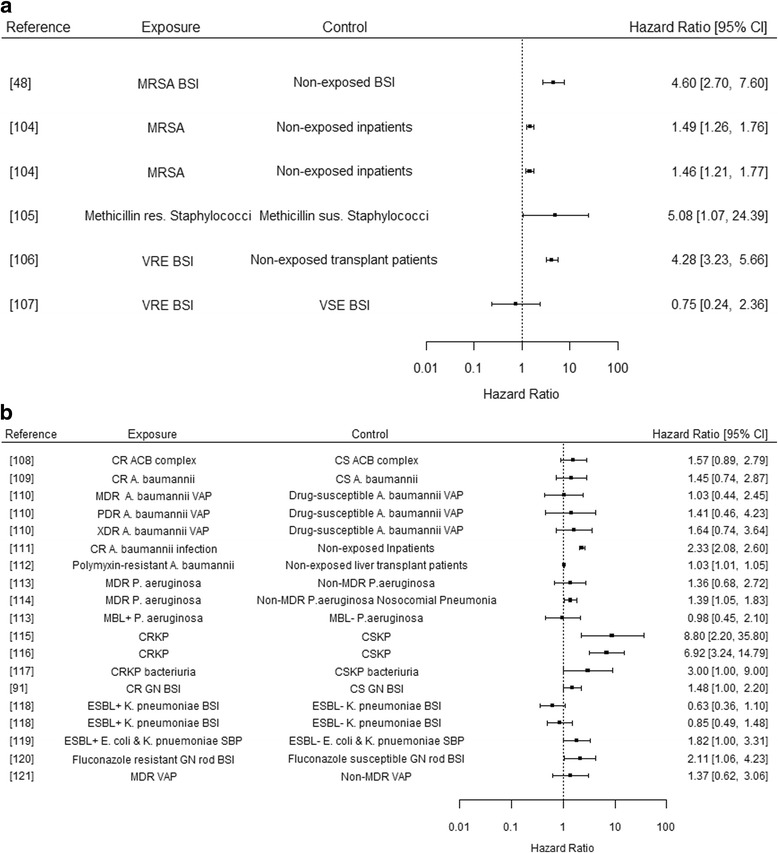
Hazard Ratios of Mortality Outcomes for Resistant Infections. Results presented are from studies utilising Cox proportional hazards regression techniques, where 1.0 represents the point at which exposure and control experience the same event rate at any point in time. The box point represents the reported HR value, with horizontal lines representing the reported 95% Confidence Interval. Results have not been adjusted or adapted to represent sample size, and are presented grouped by genera. **a** Gram-positive Bacteria. **b** Gram-negative Bacteria. [48, 91, 104–121]

Comparing Figs. 2 and 3 would suggest there is no clear consensus from either method (parametric regression or Cox proportional hazards regression) as to whether there is a significant impact of bacterial resistance mechanisms on mortality outcomes, though it should be noted that different measures of mortality have been used across these studies (see Additional file 1: Table S4). 32% (8/25) of these studies estimating mortality-related hazard ratios, did so in relation to in-hospital mortality. Such analysis may not accurately estimate the impact of exposure (versus non-exposure) on outcome, due to the failure of the non-informative censoring assumption, as the cause of being discharged may be related to the likelihood of experiencing death. As the infection type, resistance type and outcomes are different across the aforementioned studies a rigorous comparison cannot be made.

Only one study estimated the potential patient burden of AMR via secondary effects (secondary patient burden), by evaluating the impact on mortality (excess deaths) of reduced prophylactic antibiotic efficacy in the United States [12]. Utilising an evidence synthesis and stepwise calculation approach, this study estimated that a 30% reduction in the efficacy of antibiotic prophylaxis for certain surgical and chemotherapeutic procedures would result in 6300 infection-related deaths annually [12].

### Estimating the healthcare system burden of AMR

**Fig. 4:**
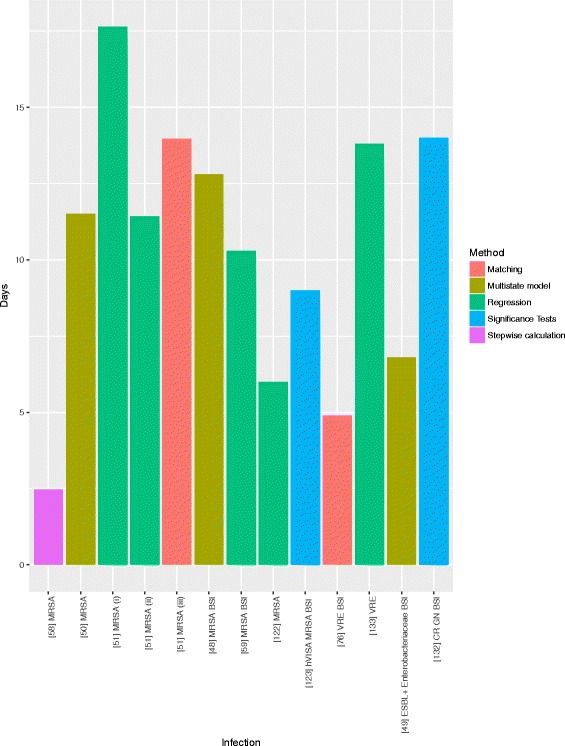
Estimates of Excess Length of Stay of Hospital/ICU Stay Caused by Antimicrobial Resistance. (i) - (iii) denote different methods used in a single study [48–51, 58, 59, 76, 122, 123, 132 ,133]

For estimating the healthcare system burden (which incorporates the payer and provider perspectives) of AMR, regression analyses and significance tests were the most utilised methods. Seventy-five studies estimated healthcare system burden, 64 of these estimated LoS burden due to AMR and 31 estimated monetary cost (with some studies estimating both). The majority of studies evaluating LoS (69%, 44/64) found that resistance had a statistically significant impact, however 39% (17/44) of these utilised significance testing against descriptive statistics (such as median length of stay). Thirteen studies estimated excess LoS due to resistant infections (Fig. 4), with 3 studies using a multistate modelling methodology to estimate LoS. Multistate models attempt to adjust for time dependency bias [48–50], however this bias has also been adjusted for in other ways, such as shifting forward the index date from the date of hospital admission to the first day of the second calendar month of the patient’s stay [51]. The majority of excess LoS estimates were for Gram-positive bacteria, with only two estimates for a Gram-negative bacteria (Fig. 4). Excess LoS was estimated at 12.8 days for MRSA bloodstream infections [95% CI 6.2 - 26.1 days] [48] in Australia, 11.5 [95% CI: 7.9-15] days for MRSA in Switzerland [49] and 11.43 [95% CI; 10.44 – 12.43] days for MRSA in American Veterans, utilising time dependency adjusted methods described above [50]. These are similar to those computed by regression but much higher than that estimated by stepwise calculations (Fig. 4), though all are from different populations.

A variety of methods have been used to calculate healthcare system monetary costs, with no clear majority of one method seen (Table 3). After conversion to 2013 USD for comparing excess/attributable costs, for Extended-Spectrum beta-Lactamase (ESBL) in bloodstream infections, a matching study found no significant impact on healthcare system costs [52], whilst a multistate model found an associated $10,154 loss per case [49]. For MRSA estimates ranged from non-significance [53] to $28,553 per case [51] (across different populations for different infections, Table 3).

**Table 3:** Excess Healthcare system Cost Estimates of Antimicrobial Resistance

Study	Exposure Group	Control Group	Country	Method	Excess Cost Estimate (2013 USD)
Cost per case					
[109]	CRAB in Columbia	Carbapenem susceptible A. baumannii	Columbia	Regression	4,583^a^
[124]	ESBL+ *E. coli* & Klebsiella species UTI	ESBL- *E. coli* & Klebsiella species UTI	USA	Significance Tests	3,237^a^
[125]	ESBL+ *E.coli* BSI	ESBL- *E.coli* BSI	Germany	Matching	-2081
[49]	ESBL+ *Enterobacteriaceae* BSI	ESBL- *Enterobacteriaceae* BSI	Switzerland	Multistate model	10,154
[45]	ESBL+ UTI	ESBL- UTI	Spain	Matching & Regression	3146†
[126]	ESBL+ and/or beta-lactamases resistant UTI	Susceptible UTI	Turkey	Significance Tests	90^a^
[126]	Ciprofloxacin resistant UTI	Ciprofloxacin susceptible UTI	Turkey	Significance Tests	114^a^
[127]	MDR *A. baumannii* BSI	Susceptible *A. baumannii* BSI	Turkey	Significance Tests	15,365
[58]	MRSA	Non-exposure inpatients	Germany	Stepwise Calculations	11,878
[53]	MRSA breast abscess	MSSA breast abscess	USA	Matching	515
[128]	MRSA BSI	Non-exposure BSI	South Korea	Stepwise Calculations	5216
[129]	MRSA BSI (survivors)	Non-nosocomial-infected patients	South Korea	Matching	11,627
[129]	MRSA BSI (non-survivors)	Non-nosocomial-infected patients	South Korea	Matching	15,254
[51]	MRSA infections	Non-exposure inpatients	USA	Matching	28,553^a^
[130]	MRSA colonisation & infection	Non-exposure inpatients	USA	Matching	12,167^a^
[131]	Resistant BSI	Susceptible BSI	India	Significance Tests	912^a^
[132]	Carbapenem-resistant device associated healthcare acquired infections ICU patients	Non-“device associated healthcare acquired infections” ICU patients	Greece	Significance Tests	3,884^a^
[133]	VRE colonisation & infections	Non-exposure inpatients	Canada	Matching & Regression	18,631^a^
[76]	VRE BSI	VSE BSI	Australia	Matching & Regression	30,093^a^
[134]	VRE BSI in allo-HSCT recipients	Non-exposure in allo-HSCT recipients	USA	Significance Tests	6104
[135]	MDR TB	Non-MDR TB	Germany	Stepwise Calculations	86,321
[136]	MDR TB	Non-MDR TB	South Africa	Stepwise Calculations	6728
[137]	MDR TB	Non-MDR TB	Latvia	Regression	33291^a^
[138]	XDR and pre-XDR TB	Rifampicin-mono-resistant or MDR TB	South Africa	Stepwise Calculations	15,567
[136]	XDR TB	Non-“XDR or MDR” TB	South Africa	Stepwise Calculations	26,989
[139]	VRE BSI in leukaemia patients	Non-exposure leukaemia patients	USA	Matching	88150^a^
Per-patient per-day					
[50]	MRSA in Switzerland	Non-exposure inpatients	Switzerland	Multistate model	867
[140]	Resistant Gram-negative Bacilli infection	Susceptible Gram-negative Bacilli infection	Singapore	Matching	812
Annual cost per stated country or stated region					
[57]	MRSA	No-MRSA	USA	Multistate model	1,382,733,079
[141]	Resistant Streptococcus pneumonia	Susceptible Streptococcus pneumonia	USA	Multistate model	91,773,500
[142]	Artemisinin resistant malaria	No-“Artemisinin resistant malaria”	High endemicity region	Multistate model	32,000,000

^a^Statistically significant where *p*-value is less than 0.05

### Estimating the economic burden of AMR

From the economic perspective stepwise calculations, multistate modelling and economic models were commonly used (Table 2). Only a small number of studies were found in this perspective category, with 11 studies assessing the burden to the economy resulting from AMR. Eight of these reported an estimate for the monetary impact of resistance (Table 4). In addition, two studies found investigated the potential psycho-social impact of having multi-drug resistant Tuberculosis, concluding that many patients in the qualitative study felt that multi-drug resistant Tuberculosis increased stigma and social isolation [54], and increased the odds of incurring catastrophic costs (in which [OR = 1.61 (95% CI = 0.98–2.64), *p* < 0.06]) [43]. A report from ‘The Review on Antimicrobial Resistance’ did mention the secondary effects of AMR from the economic perspective, however did not attempt to quantify an exact figure for this, instead stating that some proportion of the 120 trillion USD gained from caesareans, joint operations, chemotherapy and organ transplants could be lost [1].

**Table 4:** Excess Economic Burden Estimates of Antimicrobial Resistance

Study	Exposure Group	Control Group	Country	Method	Excess Cost Estimate (2013 USD)
Cost per case					
[135]	MDR TB	Susceptible TB	Germany	Stepwise Calculation	110,063
[135]	XDR TB	Susceptible TB	Germany	Stepwise Calculation	145,679
[143]	MDR TB	Susceptible TB	Europe	Stepwise Calculation	62,931
[143])	XDR TB	Susceptible TB	Europe	Stepwise Calculation	215,038
[56]	MRSA BSI	Non-nosocomial-infected patients	South Korea	Matching	21,832
Annual cost per stated country or stated region					
[141]	Resistant Streptococcus pneumonia	Susceptible Streptococcus pneumonia	USA	Multistate model	236,495,000
[57]	MRSA	No MRSA	USA	Multistate model	7,848,223,600
[142]	Artemisinin resistant malaria	No resistance	High endemicity regions	Decision Tree	385,000,000
Global economic cost					
[15]	Resistance globally (doubling of current infection rates and 100% resistance)	Lower rates of resistance (a 40% resistance increase from current rates)	Global	Total Factor Productivity model	14,228,000,000 less GDP produced in 2050 compared to 2050 in a scenario with lower resistance
[55]	Resistance globally (100% resistance rate)	No resistance	Global	Computable General Equilibrium model	3,158,862,360 less GDP produced in 2050 compared to 2050 with no resistance

The only studies explicitly estimating the economic burden of AMR to include Gram-negative infections were those commissioned by ‘The Review on Antimicrobial Resistance’ (in which *Escherichia coli* and *Klebsiella pnuemoniae* were two out of six studied microbes) [1, 15, 55]. These modelling studies, which utilised economic modelling techniques, provided the largest estimates of cost (though of six resistant microbes), estimating over $14 billion to over $3 trillion (2013 USD) in loss to global GDP by 2050 [15, 55]. Whilst the studies which utilised multistate modelling techniques (including decision tree analysis) or stepwise calculations, estimated national costs for a specific resistance type to range from over $20,000 per MRSA bloodstream infection case to over $7 billion per year attributable to community acquired MRSA in the United States (Table 4) [56, 57].

#### Quality of included studies

**Fig. 5:**
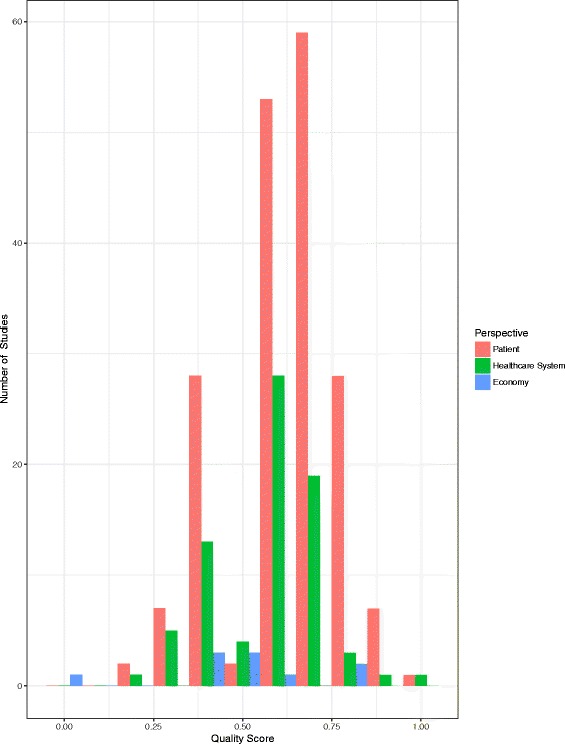
Histograms of Quality Assessment Scores by Study Perspective

For the outcomes and exposures tested relevant to this review, 199 studies were considered cohort, 1 case-control and 13 modelling studies, whereby the stated quality checklists were then applied. One additional study was a survey-based qualitative study and was not quality assessed [54]. Median quality scores (with interquartile range (IQR)) for health, healthcare system and economic burden studies were 0.67 (0.56-0.67), 0.56 (0.46-0.67) and 0.53 (0.44-0.60) respectively. Within all perspectives, quality rarely exceeded 0.75. Notably there was a lack of economic burden studies and their median quality score was lower than that of the health and healthcare system studies (Fig. 5).

The Newcastle-Ottawa checklist criteria showed that only 54% of the total available points for adjustment/comparability of different exposure groups across all cohort studies were awarded (in all 199 studies, where two points were available per study [29]), suggesting a need for more robust analyses estimating the impact of AMR. Some of the least fulfilled quality assessment categories by studies were those that focused on representativeness of the sample utilised (18% of the appraised studies met this criteria), description/adequacy of follow-up or non-response rates (40% met this criteria) and demonstration of non-exposure before study period (8% met this criteria), across the Newcastle-Ottawa cohort quality assessment checklist (199 studies).

For the 13 studies for which the Philips checklist was utilised to assess quality, there were many indicators for which scores were generally low, including those related to descriptions of the choice of model structure and the approach to obtaining parameter values (less than 1/3 of studies obtaining the related criteria). However, of note, none of the relevant studies met the following criteria; “Have the four principal types of uncertainty been addressed?”, “If not, has the omission been justified?” and “Is there evidence that the mathematical logic of the model has been tested thoroughly before use?” [31]. Additionally, only 23% of studies used a lifetime horizon or justified why they didn’t use it and only 15% demonstrated a systematic approach to parameter selection.

**Box 1: Across Perspectives: A Tuberculosis Case Study**

**Table:** 

To illustrate the importance of clarifying the perspective chosen when investigating the burden of AMR, the case of multidrug resistant (MDR-) and extensively drug resistant (XDR-) Tuberculosis (TB) will be used, as studies of this infection-type provided cost estimates (monetary costs are 2013 USD) across perspectives (for full study details refer to Additional file 1: Table S4).
**Patient burden:** In Peruvian adult patients, it was estimated that MDR-TB was significantly associated with mortality [HR = 7.5 (95% CI; 4.1 – 13.4)] using regional network data [144]. Likewise, in American patients, using data from a national institute, XDR was found to be significantly associated with mortality [HR = 2.8 (95% CI; 1.4 – 5.4)] [145]. In South African adult patients from one hospital, both MDR and XDR were associated with increased mortality [MDR HR = 3.37, *p* < 0.0001, and XDR HR = 6.75, *p* < 0.001] [146]. All of the aforementioned studies looking into TB and mortality utilised a Cox proportional hazards approach. Another study utilised a Kaplan-Meier survival analysis methodology, and found that capromycin resistance in XDR TB was not significantly associated with mortality (*p* < 0.0573) [147]. Using Israeli patient data from a national registry, MDR was found to be significantly associated with TB-related death [OR = 2.83(95% CI; 1.70-4.72), p < 0.001], using a logistic regression approach [148].
**Healthcare system burden:** MDR-TB was estimated to cost the South African healthcare system $6728 excess per case, $33,291 total per case in Latvia and $86,321 total per case in Germany using an evidence synthesis approach [135–137]. These studies included factors such as length of stay, drug costs and services consumed (varying between studies).
**Economic burden:** It was estimated, by evidence synthesis and stepwise calculation, that the total economic and societal burden costs per case for the original EU-15 states (i.e. healthcare system costs plus productivity loss, compared to susceptible TB) from MDR and XDR TB were $62,931 and $215,038 [143]. MDR-TB was also found to be independently associated with incurring catastrophic costs in Peruvian patients older than 15 years [OR = 1.61 (95% CI = 0.98–2.64)], whereby catastrophic costs were defined as total costs greater than or equal to 20% of household annual income. Costs included direct medical and non-medical out-of-pocket costs (such as excess transport and food) [43].

## Discussion

The first aim of this review was to establish what perspectives and resulting methodologies have been used to estimate AMR burden in recent literature and to discern the impact this has on the actual estimates of burden. This review found that out of the 214 studies included, 187 studies provided an estimate of burden from the health perspective, 75 studies from the healthcare system perspective and 11 studies from the economic perspective. This review describes the large range of estimates that fall within these categories, and the methodologies used to obtain them. The most utilised methodologies were regression analysis for patient health and healthcare system burden calculations and either stepwise calculations or multistate models for economic burden estimates. An additional aim of this paper was to assess the quality of evidence for health, healthcare system and economic burden. There was a lack of economic burden studies, with the median quality score of these studies being lower than those of the health and healthcare system studies.

AMR is thought to potentially impact patient health through increasing patient mortality, though only around 50% of studies found a statistically significant impact when comparing resistant and susceptible infections. A substantial proportion of studies which used parametric regression or Cox proportional hazards techniques to estimate the impact of resistant infections in Gram-negative bacteria on mortality through OR or HR measures, had high uncertainty.

Evidence on additional length of hospital stay, a key driver of cost of infections in hospitals, was variable in terms of methodological choice and values found. A large proportion of the studies addressing length of stay in a healthcare facility did not explicitly state excess or associated length of stay, but rather estimated average length of stay for different groups and performed univariate comparisons of these averages. For those that did estimate excess length of stay, it was expected (given previous literature [28]) that multistate model length of stay estimates compared to those found using alternative methods would be more conservative. However, this review found that multistate model estimates for length of stay for MRSA infection and MRSA bloodstream infection were higher than those using stepwise analysis and regression analysis [48, 50, 58, 59]. This is likely due to the fact that the described studies were set in different countries (Australia, Germany, Switzerland and US) and were mainly single centre studies, and so less externally valid. The use of multistate modelling methods to estimate attributable health and cost burden for resistant infections has previously been recommended because standard regression techniques may overstate the attributable length of stay for hospital onset infections, thus overestimating burden of AMR [28, 60]. However, we demonstrate that very few studies took advantage of this method over the search period.

Due to the small sample of economic burden studies, no consensus on method can be stated for estimating the economic burden, though 6 out of the 11 studies did utilise evidence synthesis and stepwise approaches or multistate models. The median quality of studies quantifying the economic burden of AMR fell below those of health and healthcare system burden studies, seemingly mainly due to a lack of rigorous, transparent modelling studies which appropriately present or incorporate uncertainty.

The recent reports produced by the ‘The Review on Antimicrobial Resistance’ attempted to address the lack of economic burden estimates in the field of AMR [1, 15, 55]. However due to the large scope of these projects the models used provide only broad, general estimates (such as the global economic burden of AMR in general) which may be unsuitable for cost-effectiveness or resource allocation models. The review itself calls for the estimates to be developed using more data-driven approaches [1]. This type of analysis has also recently been called into question due to its lack of scientific scrutiny and transparency, questioning the methodology used [7].

Based on the results of this study, focusing on the results of the quality assessment of included studies, the following actions are recommended for future research in AMR burden;

1. Utilise data from a representative sample of the population of interest. If this is not achievable due to data limitations, create and publish a clearly defined protocol that can be utilised in other institutions. This will enable future meta-analyses to be conducted.
2. Choose an appropriate methodology that takes into account potential confounding factors (such as patient comorbidities or age) and biases (such as time dependency bias, competing risks or non-informative censoring).
3. Describe data collection, data cleaning, follow-up, response rates and/or censoring clearly, where appropriate.
4. Estimate healthcare system and economic impact where possible.
5. If performing a mathematical or economic model, clearly describe the reasons for the chosen model structure (for example by detailing a formal health economic reasoning, including for chosen time horizon) and methods of parameterisation (with structured or systematic methods preferred). In addition, it is important to discuss how methodological, structural, heterogeneity and parameter uncertainty has been addressed (or discuss why these were not addressed).

Smith and Coast reported that estimates ranged from less than $15 to around $50,000 for additional costs per patient per episode to about $20 billion per year from the societal/economic perspective (converted to 2013 USD) [8]. This review found the healthcare system excess costs ranged from just almost non-significance to over $90 million per year, whilst economic costs ranged from just over $100,000 per case to over $3 trillion total GDP loss by 2050 (in 2013 USD). Adding to the 21 studies included in the Smith and Coast 2012 review, this review discusses the results from an additional 214 studies. This review encountered a similar result to that of Smith and Coast in terms of lack of indirect burden evidence [8]. However this review did find additional evidence on the potential secondary effects of AMR, with studies estimating the potential secondary effect of AMR on health and economic burden [1, 12].

Strengths of this review include its rigorous systematic methodology and its use of accepted quality assessment scales to present the first systematic picture of the quality of recent AMR burden estimates. This is particularly important when establishing the current evidence, as previously published review studies in this area have either been commentary pieces or have not incorporated any quality assessment across all three perspectives [8, 9, 18]. One limitation of this study is that is has a relatively short search time period. This was chosen to build upon rather than duplicate the evidence produced within a previous review, and to capture recent evidence [8]. Other limitations of the review include that the Health Related Quality of Life was not included as an outcome of interest. This was beyond the scope of this review given the very different methodologies applied in order to estimate Health Related Quality of Life burden, and that the majority of discussion of patient burden currently surrounds mortality impact.

## Conclusion

This study concludes that perspective affects chosen methodology and outcome for quantifying the burden of AMR. The review finds substantially more research in patient burden and seemingly more of a consensus on the most appropriate methods to use (regression or survival techniques), in comparison to healthcare system and economic burden research. However, across patient and healthcare system burden studies, a worryingly high number of studies are utilising univariate statistical significance tests, suggesting that a high proportion of this evidence is unreliable. The review also concludes that the evidence on the economic burden of AMR is not substantial, whereby the majority of studies have not used established health economic modelling techniques or adhered well to the Philip’s checklist [31]. More evidence on the secondary effect of AMR on health, healthcare system and economic burden is also needed [1, 12].

The estimates presented in this review can be used as parameter inputs in future health economics models used to inform health policy, whilst the description of previous methods used can inform future researchers’ methodology choice (based on their desired perspective). The review also highlights key areas where research is needed, including multivariate, internally and externally valid health and healthcare system burden studies. This research is needed particularly for Gram-negative bacteria. Additionally, high quality economic burden and secondary burden research is needed in general. Future AMR burden research should follow the recommendations highlighted in this review, in order to increase the quality of evidence available.

## Additional file

Additional file 1: Supplementary Material for the Systematic Literature Review. (DOCX 359 kb)

## Abbreviations

3GCR: Third-generation cephalosporin resistant
*A. baumannii*: *Acinetobacter baumannii*
AC: Amoxicilline-clavulanate
Allo-HSCT: Allogeneic hematopoietic stem cell transplantation
AMR: Antimicrobial Resistance
BIVR: Beta-lactam induced vancomycin resistance
BSI: Bloodstream infection
CR: Carbapenem resistant
CRAB: Carbapenem-resistant *Acinetobacter baumannii*
CRKP: Carbapenem-resistant *Klebsiella pneumoniae*
*E. coli*: *Escherichia coli*
ESBL: Extended Spectrum Beta-lactamase
EUR: Euro
GDP: Gross Domestic Product
GN: Gram-negative
HR: Hazard ratio
hVISA: Heterogeneous vancomycin-intermediate *Staphylococcus aureus*
ICU: Intensive care unit
IQR: Interquartile range
*K. pneumoniae*: *Klebsiella pneumoniae*
KPC: *Klebsiella pneumoniae* Carbapenemase
LoS: Length of stay
MBL: Metallo-beta-lactamase
MDR: Multidrug resistance
MIC: Minimum inhibitory concentration
MLSR: Macrolide-lincosamide-streptogramin resistance
MRSA: Methicillin resistant *Staphylococcus aureus*
MSSA: Methicillin susceptible *Staphylococcus aureus*
NDM-1: New Delhi Metallo-beta-lactamase-1
OR: Odds ratio
*P. aeruginosa*: *Pseudomonas aeruginosa*
PDR: Pan drug resistant
res.: Resistant
*S. aureus*: *Staphylococcus aureus*
sus.: Susceptible
TB: Tuberculosis
USD: United States Dollars
UTI: Urinary tract infection
VAP: Ventilator associated pneumonia
VRE: Vancomycin resistant *Enterococcus*
VSE: Vancomycin susceptible *Enterococcus*
XDR: Extensively drug resistant

## References

[1] The AMR Review (2014). Antimicrobial Resistance : Tackling a crisis for the health and wealth of nations.

[2] Public Health England (2014). English surveillance programme for antimicrobial utilisation and resistance (ESPAUR).

[3] Centers for Disease Control and Prevention. Antibiotic resistance threats in the United States, 2013. Current. 2013:1–114.

[4] European Food Safety Authority, European Centre for Disease Prevention and Control (2016). The European Union Summary Report on antimicrobial resistance in Antimicrobial resistance in zoonotic and indicator bacteria from humans, animals and food in the European Union in 2014. EFSA J.

[5] Public Health England. New report shows stark effect of antibiotic resistance [Internet]. 2014. Available from: https://www.gov.uk/government/news/new-report-shows-stark-effect-of-antibiotic-resistance. [cited 15 May 2016].

[6] Husereau D, Drummond MPS (2013). Consolidated health economic evaluation reporting standards (CHEERS)—Explanation and elaboration: A report of the ISPOR health economic evaluations publication guidelines good reporting practices task force.

[7] de Kraker MEA, Stewardson AJ, Harbarth S (2016). Will 10 Million People Die a Year due to Antimicrobial Resistance by 2050?. PLoS Med.

[8] Smith R, Coast J (2012). The economic burden of antimicrobial resistance: why it is more serious than current studies suggest.

[9] De Angelis G, D’Inzeo T, Fiori B, Spanu T, Sganga G (2014). Burden of Antibiotic Resistant Gram Negative Bacterial Infections: Evidence and Limits. J Med Microbiol Diagn.

[10] Jacobs P, Fassbender K (1998). The measurement of indirect costs in the health economics evaluation literature. A review. Int J Technol Assess Health Care.

[11] Tam CC, O’Brien SJ (2016). Economic cost of campylobacter, norovirus and rotavirus disease in the United Kingdom. PLoS One.

[12] Teillant A, Gandra S, Barter D, Morgan DJ, Laxminarayan R (2015). Potential burden of antibiotic resistance on surgery and cancer chemotherapy antibiotic prophylaxis in the USA: A literature review and modelling study. Lancet Infect Dis.

[13] Lambert ML, Suetens C, Savey A, Palomar M, Hiesmayr M, Morales I (2011). Clinical outcomes of health-care-associated infections and antimicrobial resistance in patients admitted to European intensive-care units: A cohort study. Lancet Infect Dis.

[14] Alam MF, Cohen D, Butler C, Dunstan F, Roberts Z, Hillier S (2009). The additional costs of antibiotics and re-consultations for antibiotic-resistant Escherichia coli urinary tract infections managed in general practice. Int J Antimicrob Agents.

[15] KPMG (2014). The global economic impact of antimicrobial resistance.

[16] Yao Z, Peng Y, Chen X, Bi J, Li Y, Ye X (2015). Healthcare associated infections of Methicillin-resistant Staphylococcus aureus: A case-control-control study. PLoS One.

[17] Stewardson AJ, Allignol A, Beyersmann J, Graves N, Schumacher M, Meyer R (2016). The health and economic burden of bloodstream infections caused by antimicrobial-susceptible and non-susceptible Enterobacteriaceae and Staphylococcus aureus in European hospitals, 2010 and 2011: a multicentre retrospective cohort study. Eur Secur.

[18] Gandra S, Barter DM, Laxminarayan R (2014). Economic burden of antibiotic resistance: how much do we really know?. Clin Microbiol Infect.

[19] Cosgrove SE, Carmeli Y (2003). The Impact of Antimicrobial Resistance on Health and Economic Outcomes. Clin Infect Dis.

[20] Wilton P, Smith R, Coast J, Millar M (2002). Strategies to contain the emergence of antimicrobial resistance: a systematic review of effectiveness and cost-effectiveness. J Health Serv Res Policy.

[21] Gallagher J. Antibiotic resistance: World on cusp of “post-antibiotic era” [Internet]. BBC. 2015. Available from: http://www.bbc.co.uk/news/health-34857015. [cited 1 Nov 2016].

[22] Department of Health, Department for Environment Food and Rural Affairs (2013). UK Five Year Antimicrobial Resistance Strategy 2013 to 2018.

[23] Naylor N, Silva S, Kulasabanathan K, Atun R, Zhu N, Knight G, et al. How to estimate the burden of antimicrobial resistance: a systematic literature review [Internet]. PROSPERO. 2016. Available from: http://www.crd.york.ac.uk/PROSPERO/display_record.asp?ID=CRD42016037510. [cited 13 Jul 2016].

[24] Moher D, Liberati A, Tetzlaff J, Altman DG, The PRISMA Group (2009). Preferred Reporting Items for Systematic Reviews an Meta-Analyses: The PRISMA Statement. PLoS Med.

[25] Naylor NR, Silva S, Kulasabanathan K, Atun R, Zhu N, Knight GM (2016). Methods for estimating the burden of antimicrobial resistance : a systematic literature review protocol. Syst Rev.

[26] PubMed [Internet]. 2018. Available from: https://www.ncbi.nlm.nih.gov/pubmed/. [cited 10 Jan 2018].

[27] Takizawa C, Thompson PL, Van Walsem A (2015). Epidemiological and Economic Burden of Alzheimer ’ s Disease : A Systematic Literature Review of Data across Europe and the United States of America. J Alzheimers Dis.

[28] Graves N, Harbarth S, Beyersmann J, Barnett A, Halton K, Cooper B (2010). Estimating the cost of health care-associated infections: mind your p’s and q’s. Clin Infect Dis.

[29] Wells G., Shea B, O’Connell D, Peterson J, Welch V, Losos M, et al. The Newcastle-Ottawa Scale (NOS) for assessing the quality of nonrandomised studies in meta-analyses [Internet]. University of Ottawa. 2014. Available from: http://www.ohri.ca/programs/clinical_epidemiology/oxford.asp. [cited 1 Feb 2016].

[30] Song J, Chung K (2011). Observational Studies: Cohort and Case-Control Studies. Plast Reconstr Surg.

[31] Philips Z, Bojke L, Sculpher M, Claxton K, Golder S (2006). Good Practice Guidelines for Decision-Analytic Modelling in Health Technology Assessment. PharmacoEconomics.

[32] Higgins JPT, Green S. Cochrane Handbook for Systematic Reviews of Interventions Version 5.1.0 [updated March 2011]: The Cochrane Collaboration; 2011. Table 7.7.a: Formulae for combining groups

[33] Rate Inflation. Historical Inflation Rates for Australia (2007 to 2017) [Internet]. 2017. Available from: http://www.rateinflation.com/inflation-rate/australia-historical-inflation-rate. [cited 10 Jan 2017].

[34] Triami Media BV. Historic Inflatin - CPI inflation year pages [Internet]. inflation.eu. 2017. Available from: www.inflation.eu. [cited 12 Feb 2017].

[35] UKForex. Yearly Average Rates [Internet]. 2017. Available from: www.ukforex.co.uk/forex-tools/historical-rate-tools/yearly-average-rates. [cited 12 Nov 2017].

[36] Wickham H, Chang W, RStudio. ggplot2: Create Elegant Data Visualisations Using the Grammar of Graphics [Internet]. 2016. Available from: https://cran.r-project.org/package=ggplot2

[37] Wickham H (2011). The Split-Apply-Combine Strategy for Data Analysis. J Stat Softw.

[38] Viechtbauer W. Package “metafor.” 2017.

[39] Bodro M, Sanclemente G, Lipperheide I, Allali M, Marco F, Bosch J (2015). Impact of antibiotic resistance on the development of recurrent and relapsing symptomatic urinary tract infection in kidney recipients. Am J Transplant.

[40] Qureshi ZA, Syed A, Clarke LG, Doi Y, Shields RK (2014). Epidemiology and clinical outcomes of patients with carbapenem-resistant Klebsiella pneumoniae bacteriuria. Antimicrob Agents Chemother.

[41] Vazirani J, Wurity S, Ali MH (2015). Multidrug-resistant Pseudomonas aeruginosa keratitis: Risk factors, clinical characteristics, and outcomes. Ophthalmology.

[42] Francis JR, Blyth CC, Colby S, Fagan JM, Waring J (2014). Multidrug-resistant tuberculosis in Western Australia, 1998–2012. Med J Aust.

[43] Wingfield T, Boccia D, Tovar M, Gavino A, Zevallos K, Montoya R (2014). Defining Catastrophic Costs and Comparing Their Importance for Adverse Tuberculosis Outcome with Multi-Drug Resistance: A Prospective Cohort Study, Peru. PLoS Med.

[44] Rello J, Molano D, Villabon M, Reina R, Rita-Quispe R, Previgliano I (2013). Differences in hospital- and ventilator-associated pneumonia due to Staphylococcus aureus (methicillin-susceptible and methicillin-resistant) between Europe and Latin America: a comparison of the EUVAP and LATINVAP study cohorts. Med Intensiva.

[45] Esteve-Palau E, Solande G, Sanchez F, Sorli L, Montero M, Guerri R (2015). Clinical and economic impact of urinary tract infections caused by ESBL-producing Escherichia coli requiring hospitalization: A matched cohort study. J Infect.

[46] Ong SJ, Huang Y-C, Tan H-Y, Ma DHK, Lin H-C, Yeh L-K (2013). Staphylococcus aureus Keratitis: A Review of Hospital Cases. PLoS One.

[47] Kini AR, Shetty V, Kumar AM, Shetty SM, Shetty A (2013). Community-associated, methicillin-susceptible, and methicillin-resistant Staphylococcus aureus bone and joint infections in children. J Pediatr Orthop B.

[48] Barnett AG, Page K, Campbell M, Martin E, Rashleigh-Rolls R, Halton K (2013). The increased risks of death and extra lengths of hospital and ICU stay from hospital-acquired bloodstream infections: a case-control study. BMJ Open.

[49] Stewardson A, Fankhauser C, De Angelis G, Rohner P, Safran E, Schrenzel J (2013). Burden of bloodstream infection caused by extended-spectrum β-lactamase-producing enterobacteriaceae determined using multistate modeling at a Swiss University Hospital and a nationwide predictive model. Infect Control Hosp Epidemiol.

[50] Macedo-Viñas M, De Angelis G, Rohner P, Safran E, Stewardson A, Fankhauser C (2013). Burden of meticillin-resistant Staphylococcus aureus infections at a Swiss University hospital: excess length of stay and costs. J Hosp Infect.

[51] Nelson RE, Samore MH, Jones M, Greene T, Stevens VW, Liu CF (2015). Reducing Time-dependent Bias in Estimates of the Attributable Cost of Health Care-associated Methicillin-resistant Staphylococcus aureus Infections: A Comparison of Three Estimation Strategies. Med Care.

[52] Leistner R, Gürntke S, Sakellariou C, Denkel LA, Bloch A, Gastmeier P (2014). Bloodstream infection due to extended-spectrum beta-lactamase (ESBL)-positive K. pneumoniae and E. coli: an analysis of the disease burden in a large cohort. Infection.

[53] Branch-Elliman W, Lee GM, Golen TH, Gold HS, Baldini LM, Wright SB (2013). Health and Economic Burden of Post-Partum Staphylococcus aureus Breast Abscess. PLoS One.

[54] Morris MD, Quezada L, Bhat P, Moser K, Smith J, Perez H (2013). Social, Economic, and Psychological Impacts of MDR-TB Treatment in Tijuana, Mexico: A Patient’s Perspective. Int J Tuberc Lung Dis.

[55] Taylor J, Hafner M, Yerushalmi E, Smith R, Bellasio J, Vardavas R (2014). Estimating the economic costs of antimicrobial resistance.

[56] Lee JY, Chong YP, Kim T, Hong HL, Park SJ, Lee ES (2014). Bone and joint infection as a predictor of community-acquired methicillin-resistant Staphylococcus aureus bacteraemia: A comparative cohort study. J Antimicrob Chemother.

[57] Lee BY, Singh A, David MZ, Bartsch SM, Slayton RB, Huang SS (2013). The economic burden of community-associated methicillin-resistant Staphylococcus aureus (CA-MRSA). Clin Microbiol Infect.

[58] Hübner C, Hübner NO, Hopert K, Maletzki S, Flessa S (2014). Analysis of MRSA-attributed costs of hospitalized patients in Germany. Eur J Clin Microbiol Infect Dis.

[59] Shorr AF, Zilberberg MD, Micek ST, Kollef MH (2015). Outcomes associated with bacteremia in the setting of methicillin-resistant Staphylococcus aureus pneumonia: a retrospective cohort study. Crit Care.

[60] Graves N, Weinhold D, Roberts JA (2005). Correcting for bias when estimating the cost of hospital-acquired infection: an analysis of lower respiratory tract infections in non-surgical patients. Health Econ.

[61] Rieg S, Jonas D, Kaasch AJ, Porzelius C, Peyerl-Hoffmann G, Theilacker C (2013). Microarray-Based Genotyping and Clinical Outcomes of Staphylococcus aureus Bloodstream Infection: An Exploratory Study. PLoS One.

[62] Manandhar S, Pai G, Gidwani H, Nazim S, Buehrle D, Shutt KA (2016). Does Staphylococcus aureus Bacteriuria Predict Clinical Outcomes in Patients With Bacteremia? Analysis of 274 Patients With Staphylococcus aureus Blood Stream Infection. Infect Dis Clin Pract.

[63] Theodorou P, Lefering R, Perbix W, Spanholtz TA, Maegele M, Spilker G (2013). Staphylococcus aureus bacteremia after thermal injury: The clinical impact of methicillin resistance. Burns.

[64] Cobos-Carrascosa E, Soler-Palacín P, Nieves Larrosa M, Bartolomé R, Martín-Nalda A, Antoinette Frick M (2015). Staphylococcus aureus Bacteremia in Children. Pediatr Infect Dis J.

[65] Hernández C, Feher C, Soriano A, Marco F, Almela M, Cobos-Trigueros N (2014). Clinical characteristics and outcome of elderly patients with community-onset bacteremia. The Journal of infection.

[66] Okada Y, Hosono M, Sasaki Y, Hirai H, Suehiro S (2014). Preoperative increasing C-reactive protein affects the outcome for active infective endocarditis. Ann Thorac Cardiovasc Surg.

[67] Yavuz SŞ, Şensoy A, Çeken S, Deniz D, Yekeler I (2014). Methicillin-resistant Staphylococcus aureus infection: An independent risk factor for mortality in patients with poststernotomy mediastinitis. Med Princ Pract.

[68] Sakoda Y, Ikegame S, Ikeda-Harada C, Takakura K, Kumazoe H, Wakamatsu K (2014). Retrospective analysis of nursing and healthcare-associated pneumonia: Analysis of adverse prognostic factors and validity of the selection criteria. Respiratory Investigation.

[69] Takata T, Miyazaki M, Futo M, Hara S, Shiotsuka S, Kamimura H (2013). Presence of both heterogeneous vancomycin-intermediate resistance and β-lactam antibiotic-induced vancomycin resistance phenotypes is associated with the outcome in methicillin-resistant Staphylococcus aureus bloodstream infection. Scand J Infect Dis.

[70] Hu H-C, Kao K-C, Chiu L-C, Chang C-H, Hung C-Y, Li L-F (2015). Clinical outcomes and molecular typing of heterogenous vancomycin-intermediate Staphylococcus aureus bacteremia in patients in intensive care units. BMC Infect Dis.

[71] Lee H-Y, Chen C-L, Liu S-Y, Yan Y-S, Chang C-J, Chiu C-H (2015). Impact of Molecular Epidemiology and Reduced Susceptibility to Glycopeptides and Daptomycin on Outcomes of Patients with Methicillin-Resistant Staphylococcus aureus Bacteremia. PLoS One.

[72] Lee JK, Lee J, Park YS, Lee CH, Yim JJ, Yoo CG (2015). Clinical manifestations of pneumonia according to the causative organism in patients in the intensive care unit. Korean J Intern Med.

[73] Hope R, Blackburn RM, Verlander NQ, Johnson A, Kearns A, Hill R (2013). Vancomycin MIC as a predictor of outcome in MRSA bacteraemia in the UK context. J Antimicrob Chemother.

[74] Tadros M, Williams V, Coleman BL, McGeer AJ, Haider S, Lee C (2013). Epidemiology and Outcome of Pneumonia Caused by Methicillin-Resistant Staphylococcus aureus (MRSA) in Canadian Hospitals. PLoS One.

[75] Castón JJ, González-Gasca F, Porras L, Illescas S, Romero MD, Gijón J (2014). High vancomycin minimum inhibitory concentration is associated with poor outcome in patients with methicillin-susceptible Staphylococcus aureus bacteremia regardless of treatment. Scand J Infect Dis.

[76] Cheah ALY, Spelman T, Liew D, Peel T, Howden BP, Spelman D (2013). Enterococcal bacteraemia: Factors influencing mortality, length of stay and costs of hospitalization. Clin Microbiol Infect.

[77] Kang C-I, Song J-H, Kim SH, Chung DR, Peck KR, Thamlikitkul V (2013). Association of levofloxacin resistance with mortality in adult patients with invasive pneumococcal diseases: a post hoc analysis of a prospective cohort. Infection.

[78] Fu Q, Ye H, Liu S (2015). Risk factors for extensive drug-resistance and mortality in geriatric inpatients with bacteremia caused by Acinetobacter baumannii. Am J Infect Control.

[79] Kitazono H, Rog D, Sa G, Nm C, Acinetobacter RGE. Acinetobacter baumannii infection in solid organ transplant recipients. Clin Transpl. 2015:227–32. 10.1111/ctr.12508 25580999

[80] Park SY, Choo JW, Kwon SH, Yu SN, Lee EJ, Kim TH (2013). Risk Factors for Mortality in Patients with Acinetobacter baumannii Bacteremia. Infect Chemother.

[81] De Jager P, Chirwa T, Naidoo S, Perovic O, Thomas J (2015). Nosocomial outbreak of New Delhi metallo-Beta-lactamase-1-producing Gram-negative bacteria in South Africa: A case-control study. PLoS One.

[82] Zhang HT, Liu H (2015). Laboratory-based evaluation of MDR strains of Pseudomonas in patients with acute burn injuries. Int J Clin Exp Med.

[83] Samonis G, Vardakas KZ, Kofteridis DP, Dimopoulou D, Andrianaki AM, Chatzinikolaou I (2014). Characteristics, risk factors and outcomes of adult cancer patients with extensively drug-resistant Pseudomonas aeruginosa infections. Infection.

[84] Theodorou P, Thamm O, Perbix W, Phan V (2013). Pseudomonas aeruginosa bacteremia after burn injury: The impact of multiple-drug resistance. J Burn Care Res.

[85] Micek ST, Kollef MH, Torres A, Chen C, Rello J, Chastre J (2015). Pseudomonas aeruginosa Nosocomial Pneumonia: Impact of Pneumonia Classification. Infect Control Hosp Epidemiol.

[86] Keddy KH, Sooka A, Musekiwa A, Smith AM, Ismail H, Tau NP (2015). Clinical and microbiological features of salmonella meningitis in a South African Population, 2003-2013. Clin Infect Dis.

[87] Tumbarello M, Trecarichi EM, De Rosa FG, Giannella M, Giacobbe DR, Bassetti M (2014). Infections caused by KPC-producing Klebsiella pneumoniae: Differences in therapy and mortality in a multicentre study. J Antimicrob Chemother.

[88] Capone A, Giannella M, Fortini D, Giordano A, Meledandri M, Ballardini M (2013). High rate of colistin resistance among patients with carbapenem-resistant Klebsiella pneumoniae infection accounts for an excess of mortality. Clin Microbiol Infect.

[89] Hussein K, Raz-Pasteur A, Finkelstein R, Neuberger A, Shachor-Meyouhas Y, Oren I (2013). Impact of carbapenem resistance on the outcome of patients’ hospital-acquired bacteraemia caused by Klebsiella pneumoniae. J Hosp Infect.

[90] Biehle LR, Cottreau JM, Thompson DJ, Filipek RL, O’Donnell JN, Lasco TM (2015). Outcomes and risk factors for mortality among patients treated with carbapenems for klebsiella spp. bacteremia. PLoS One.

[91] Andria N, Henig O, Kotler O, Domchenko A, Oren I, Zuckerman T (2015). Mortality burden related to infection with carbapenem-resistant Gram-negative bacteria among haematological cancer patients: A retrospective cohort study. J Antimicrob Chemother.

[92] Papadimitriou-Olivgeris M, Marangos M, Fligou F, Christofidou M, Sklavou C, Vamvakopoulou S (2013). KPC-producing Klebsiella pneumoniae enteric colonization acquired during intensive care unit stay: the significance of risk factors for its development and its impact on mortality. Diagn Microbiol Infect Dis.

[93] Abernethy JK, Johnson AP, Guy R, Hinton N, Sheridan EA, Hope RJ (2015). Thirty day all-cause mortality in patients with Escherichia coli bacteraemia in England. Clin Microbiol Infect.

[94] Bergin SP, Thaden JT, Ericson JE, Cross H, Messina J, Clark RH (2015). Neonatal Escherichia coli Bloodstream Infections: Clinical Outcomes and Impact of Initial Antibiotic Therapy. Pediatr Infect Dis J.

[95] Denis B, Lafaurie M, Donay JL, Fontaine JP, Oksenhendler E, Raffoux E (2015). Prevalence, risk factors, and impact on clinical outcome of extended-spectrum beta-lactamase-producing Escherichia coli bacteraemia: A five-year study. Int J Infect Dis.

[96] Kim S-H, Kwon J-C, Choi S-M, Lee D-G, Park SH, Choi J-H (2013). Escherichia coli and Klebsiella pneumoniae bacteremia in patients with neutropenic fever: factors associated with extended-spectrum β-lactamase production and its impact on outcome. Ann Hematol.

[97] Rodríguez-Baño J, Mingorance J, Fernández-Romero N, Serrano L, López-Cerero L, Pascual A (2013). Outcome of bacteraemia due to extended-spectrum β-lactamase-producing Escherichia coli: Impact of microbiological determinants. J Infect.

[98] Ha YE, Kang C-II, Cha MK, Park SY, Wi YM, Chung DR (2015). Epidemiology and clinical outcomes of bloodstream infections caused by extended-spectrum β-lactamase-producing Escherichia coli in patients with cancer. Int J Antimicrob Agents.

[99] Hernandez C, Cobos-Trigueros N, Feher C, Morata L, De La Calle C, Marco F (2014). Community-onset bacteraemia of unknown origin: clinical characteristics, epidemiology and outcome. Eur J Clin Microbiol Infect Dis.

[100] Seboxa T, Amogne W, Abebe W, Tsegaye T, Azazh A, Hailu W (2015). High mortality from blood stream infection in Addis Ababa, Ethiopia, is due to antimicrobial resistance. PLoS One.

[101] Chaulk J, Carbonneau M, Qamar H, Keough A, Chang HJ, Ma M (2014). Third-generation cephalosporin-resistant spontaneous bacterial peritonitis: a single-centre experience and summary of existing studies. Can J Gastroenterol Hepatol.

[102] Huh K, Kang CI, Kim J, Cho SY, Ha YE, Joo EJ (2014). Risk factors and treatment outcomes of bloodstream infection caused by extended-spectrum cephalosporin-resistant Enterobacter species in adults with cancer. Diagn Microbiol Infect Dis.

[103] Haeusler GM, Mechinaud F, Daley AJ, Starr M, Shann F, Connell TG (2013). Antibiotic-resistant Gram-negative Bacteremia in Pediatric Oncology Patients—Risk Factors and Outcomes. Pediatr Infect Dis J.

[104] Nelson RE, Stevens VW, Jones M, Samore MH, Rubin MA (2015). Health care associated methicillin-resistant Staphylococcus aureus infections increases the risk of postdischarge mortality. Am J Infect Control.

[105] Nitta H, Beppu T, Itoyama A, Higashi T, Sakamoto K, Nakagawa S (2015). Poor outcomes after hepatectomy in patients with ascites infected by methicillin-resistant staphylococci. Journal of Hepatobiliary-Pancreatic Sciences.

[106] Tavadze M, Rybicki L, Mossad S, Avery R, Yurch M, Pohlman B (2014). Risk factors for vancomycin-resistant enterococcus bacteremia and its influence on survival after allogeneic hematopoietic cell transplantation. Bone Marrow Transplant.

[107] Cho S-Y, Lee D-G, Choi S-M, Kwon J-C, Kim S-H, Choi J-K (2013). Impact of vancomycin resistance on mortality in neutropenic patients with enterococcal bloodstream infection: a retrospective study. BMC Infect Dis.

[108] Chusri S, Chongsuvivatwong V, Rivera JI, Silpapojakul K, Singkhamanan K, McNeil E (2014). Clinical Outcomes of Hospital-Acquired Infection with Acinetobacter nosocomialis and Acinetobacter pittii. Antimicrob Agents Chemother.

[109] Lemos EV, de la Hoz FP, Alvis N, Einarson TR, Quevedo E, Castañeda C (2014). Impact of carbapenem resistance on clinical and economic outcomes among patients with Acinetobacter baumannii infection in Colombia. Clin Microbiol Infect.

[110] Inchai J, Pothirat C, Bumroongkit C, Limsukon A, Khositsakulchai W, Liwsrisakun C (2015). Prognostic factors associated with mortality of drug-resistant Acinetobacter baumannii ventilator-associated pneumonia. Journal of Intensive Care.

[111] Henig O, Weber G, Hoshen MB, Paul M, German L, Neuberger A (2015). Risk factors for and impact of carbapenem-resistant Acinetobacter baumannii colonization and infection: matched case-control study. Eur J Clin Microbiol.

[112] Freire MP, Van Der Heijden IM, do Prado GVB, Cavalcante LS, Boszczowski I, Bonazzi PR (2014). Polymyxin use as a risk factor for colonization or infection with polymyxin-resistant *Acinetobacter baumannii* after liver transplantation. Transpl Infect Dis.

[113] Willmann M, Kuebart I, Marschal M, Schröppel K, Vogel W, Flesch I (2013). Effect of metallo-β-lactamase production and multidrug resistance on clinical outcomes in patients with Pseudomonas aeruginosa bloodstream infection: a retrospective cohort study. BMC Infect Dis.

[114] Micek ST, Wunderink RG, Kollef MH, Chen C, Rello J, Chastre J (2015). An international multicenter retrospective study of Pseudomonas aeruginosa nosocomial pneumonia: Impact of multidrug resistance. Crit Care.

[115] Simkins J, Muggia V, Cohen HW, Minamoto GY (2014). Carbapenem-resistant *Klebsiella pneumoniae* infections in kidney transplant recipients: a case-control study. Transpl Infect Dis.

[116] Pereira MR, Scully BF, Pouch SM, Uhlemann A-C, Goudie S, Emond JE (2015). Risk factors and outcomes of carbapenem-resistant Klebsiella pneumoniae infections in liver transplant recipients. Liver Transpl.

[117] Pouch SM, Kubin CJ, Satlin MJ, Tsapepas DS, Lee JR, Dube G (2015). Epidemiology and outcomes of carbapenem-resistant Klebsiella pneumoniae bacteriuria in kidney transplant recipients. Transpl Infect Dis.

[118] Gürntke S, Kohler C, Steinmetz I, Pfeifer Y, Eller C, Gastmeier P (2014). Molecular epidemiology of extended-spectrum beta-lactamase (ESBL)-positive Klebsiella pneumoniae from bloodstream infections and risk factors for mortality. J Infect Chemother.

[119] Kim MJ, Song K-H, Kim N-H, Choe PG, Park WB, Bang JH (2014). Clinical outcomes of spontaneous bacterial peritonitis due to extended-spectrum beta-lactamase-producing Escherichia coli or Klebsiella pneumoniae: a retrospective cohort study. Hepatol Int.

[120] Miles-Jay A, Butler-Wu S, Rowhani-Rahbar A, Pergam SA (2015). Incidence rate of fluoroquinolone resistant gram-negative rod bacteremia among allogeneic hematopoietic cell transplant patients during an era of levofloxacin prophylaxis. Biol Blood Marrow Transplant.

[121] Tedja R, Nowacki A, Fraser T, Fatica C, Griffiths L, Gordon S (2014). The impact of multidrug resistance on outcomes in ventilator-associated pneumonia. Am J Infect Control.

[122] Issler-Fisher AC, McKew G, Fisher OM, Harish V, Gottlieb T, Maitz PKM (2015). Risk factors for, and the effect of MRSA colonization on the clinical outcomes of severely burnt patients. Burns.

[123] Casapao AM, Leonard SN, Davis SL, Lodise TP, Patel N, Goff DA (2013). Clinical Outcomes in Patients with Heterogeneous Vancomycin-Intermediate Staphylococcus aureus Bloodstream Infection. Antimicrob Agents Chemother.

[124] MacVane SH, Tuttle LO, Nicolau DP (2014). Impact of extended-spectrum β-lactamase-producing organisms on clinical and economic outcomes in patients with urinary tract infection. J Hosp Med.

[125] Leistner R, Bloch A, Sakellariou C, Gastmeier P, Schwab F (2014). Costs and length of stay associated with extended-spectrum β-lactamase production in cases of Escherichia coli bloodstream infection. J Glob Antimicrob Resist.

[126] Sozen H, Caylak S, Cetinkaya M, Citil BE, Sahins C, Deliktas H (2015). Clinical and Economic Outcomes Associated with Urinary Tract Infections Caused by Extended Spectrum Beta-lactamase Producing Bacteria in a Tertiary Care Hospital. Stud Ethnomed.

[127] Gulen TA, Guner R, Celikbilek N, Keske S, Tasyaran M (2015). Clinical importance and cost of bacteremia caused by nosocomial multi drug resistant acinetobacter baumannii. Int J Infect Dis.

[128] Joo E-JJ, Peck KRR, Ha YEE, Kim Y-SS, Song Y-GG, Lee S-SS (2013). Impact of acute kidney injury on mortality and medical costs in patients with meticillin-resistant Staphylococcus aureus bacteraemia: a retrospective, multicentre observational study. J Hosp Infect.

[129] Kim C-J, Kim H-B, Oh M, Kim YYK, Kim A, Oh S-H (2014). The burden of nosocomial staphylococcus aureus bloodstream infection in South Korea: a prospective hospital-based nationwide study. BMC Infect Dis.

[130] Nelson RE, Jones M, Liu C-F, Samore MH, Evans ME, Graves N (2015). The Impact of Healthcare-Associated Methicillin-Resistant Staphylococcus Aureus Infections on Post-Discharge Healthcare Costs and Utilization. Infect Control Hosp Epidemiol.

[131] Chandy SJ, Naik GS, Balaji V, Jeyaseelan V, Thomas K, Lundborg CS (2014). High cost burden and health consequences of antibiotic resistance: The price to pay. J Infect Dev Ctries.

[132] Apostolopoulou E, Zikos D, Tselebis A, Drosatou X, Steganidis E, Xristodoulou A, et al. Clinical outcomes and economic variables in critically ill patients with bloodstream infections. Health Sci J. 2014;8

[133] Lloyd-Smith P, Younger J, Lloyd-Smith E, Green H, Leung V, Romney MG (2013). Economic analysis of vancomycin-resistant enterococci at a Canadian hospital: assessing attributable cost and length of stay. J Hosp Infect.

[134] Ford CD, Lopansri BK (2015). Gazdik M a, Snow GL, Webb BJ, Konopa KL, et al. The clinical impact of vancomycin-resistant Enterococcus colonization and bloodstream infection in patients undergoing autologous transplantation. Transpl Infect Dis.

[135] Diel R, Nienhaus A, Lampenius N, Rüsch-Gerdes S, Richter E (2014). Cost of multi drug resistance tuberculosis in Germany. Respir Med.

[136] Pooran A, Pieterson E, Davids M, Theron G, Dheda K (2013). What is the Cost of Diagnosis and Management of Drug Resistant Tuberculosis in South Africa?. PLoS One.

[137] Miller TTL, Cirule A, Wilson FA, Holtz TH, Riekstina V, Cain KP (2013). The value of effective public tuberculosis treatment: an analysis of opportunity costs associated with multidrug resistant tuberculosis in Latvia. Cost Eff Resour Alloc.

[138] Cox H, Ramma L, Wilkinson L, Azevedo V, Sinanovic E (2015). Cost per patient of treatment for rifampicin-resistant tuberculosis in a community-based programme in Khayelitsha, South Africa. Trop Med Int Health.

[139] Ford CD, Lopansri BK, Haydoura S, Snow G, Dascomb KK, Asch J (2015). Frequency, risk factors, and outcomes of vancomycin-resistant Enterococcus colonization and infection in patients with newly diagnosed acute leukemia: different patterns in patients with acute myelogenous and acute lymphoblastic leukemia. Infect Control Hosp Epidemiol.

[140] Vasudevan A, Memon B, Mukhopadhyay A, Li J, Tambyah P (2015). The costs of nosocomial resistant gram negative intensive care unit infections among patients with the systemic inflammatory response syndrome- a propensity matched case control study. Antimicrob Resist Infect Control.

[141] Reynolds C, Finkelstein J, Ray G, Moore M, Huang S (2014). Attributable healthcare utilization and cost of pneumoniae due to drug-resistant Streptococcus pneumoniae: a cost analysis. Antimicrob Resist Infect Control.

[142] Lubell Y, Dondorp A, Guérin PJ, Drake T, Meek S, Ashley E (2014). Artemisinin resistance – modelling the potential human and economic costs. Malar J.

[143] Diel R, Vandeputte J, De Vries G, Stillo J, Wanlin M, Nienhaus A (2014). Costs of tuberculosis disease in the European Union: A systematic analysis and cost calculation. Eur Respir J.

[144] Chung-Delgado K, Guillen-Bravo S, Revilla-Montag A, Bernabe-Ortiz A (2015). Mortality among MDR-TB cases: Comparison with drug-susceptible tuberculosis and associated factors. PLoS One.

[145] Ershova JV, Kurbatova EV, Moonan PK, Cegielski JP (2014). Mortality Among Tuberculosis Patients With Acquired Resistance to Second-line Antituberculosis Drugs--United States, 1993-2008. Clin Infect Dis.

[146] Gandhi NR, Brust JCM, Moodley P, Weissman D, Heo M, Ning Y (2014). Minimal diversity of drug-resistant Mycobacterium tuberculosis strains, South Africa. Emerg Infect Dis.

[147] O’Donnell MR, Pillay M, Pillay M, Werner L, Master I, Wolf A (2015). Primary capreomycin resistance is common and associated with early mortality in patients with extensively drug-resistant tuberculosis in KwaZulu-Natal, South Africa. J Acquir Immune Defic Syndr.

[148] Shuldiner J, Leventhal A, Chemtob D, Mor Z (2014). Mortality of tuberculosis patients during treatment in israel, 2000-2010. Int J Tuberc Lung Dis.

