# Supplementary material for: Estimating the burden of antimicrobial resistance: a systematic literature review
Source: Antimicrob Resist Infect Control. 2018 Apr 25;7:58. doi: 10.1186/s13756-018-0336-y (PMC5918775; doi:10.1186/s13756-018-0336-y)
Supplement: Supplementary file 1 — Supplementary Material for the Systematic Literature Review. (DOCX 359 kb) [file 13756_2018_336_MOESM1_ESM.docx]

**Supplementary Material**

**Quality Assessment Checklists**

For the quantification of a quality score, one point was given for each * achieved in the below checklists, the total points awarded to a given manuscript was divided by the total number of points available for that checklist. For example, if a study achieved 7 checklist points for the Newcastle-Ottawa Quality Assessment Scales for case control studies [1] out of the available 8, it’s quality assessment score would be 0.88 (7/8).

1. Case control & Cohort studies

For case control and cohort studies the Newcastle-Ottawa Quality Assessment Scale was applied [1]. Assumptions that were made regarding the application of the scale to this review are within the Tables 1 and 2.

**Table 1: Quality Assessment Checklist for Case control Studies**

| **Domain** | **Checklist Criteria** | **Additional Information** |
| --- | --- | --- |
| Selection | 1) Is the case definition adequate?  a) yes, with independent validation *****  b) yes, eg record linkage or based on self reports  c) no description |  |
|  | 2) Representativeness of the cases  a) consecutive or obviously representative series of cases *  b) potential for selection biases or not stated | Assumption: Given the nature of the review, studies received a star if they discuss representation (and reasons for their study being representative) or if they are multicentre/regional/national. |
|  | 3) Selection of Controls  a) community controls *****  b) hospital controls  c) no description | Assumption: It was assumed that if the study was in a hospital setting in which cases were hospital patients, hospital controls were accepted. |
|  | 4) Definition of Controls  a) no history of disease (endpoint) *****  b) no description of source | Assumption: History of disease/infection was used in this criteria even in studies looking at mortality or other burden outcomes [2] |
| Comparability | 1) Comparability of cases and controls on the basis of the design or analysis  a) study controls for age/sex/comorbidities *  b) study controls for any additional factor ***** | 2 * maximum allotted for this criteria.  Assumption: For studies in which hospital associated cases and LoS were being analysed, two stars were only given if time dependency was controlled for. |
| Exposure | 1) Ascertainment of exposure  a) secure record (eg surgical records) *****  b) structured interview where blind to case/control status *****  c) interview not blinded to case/control status  d) written self report or medical record only  e) no description | Assumption: Studies which utilised lab techniques were used to ascertain exposure received one star. |
|  | 2) Same method of ascertainment for cases and controls  a) yes *****  b) no  3) Non-Response rate  a) same rate for both groups *****  b) non respondents described  c) rate different and no designation | 3) Assumption: No description of data cleaning or linkage and loss to missing data for retrospective studies was panelised by not awarding a star. |

**Table 2 Quality Assessment Checklist for Cohort Studies**

| **Domain** | **Checklist Criteria** | **Additional Information** |
| --- | --- | --- |
| Selection | 1) Representativeness of the exposed cohort  a) truly representative of the average population in the community *  b) somewhat representative of the average population in the community *  c) selected group of users eg nurses, volunteers  d) no description of the derivation of the cohort | 1 * maximum allotted for this criteria.  Assumption: Given the nature of the review, studies received a star if they discuss representation (and reasons for their study being representative) or if they are multicentre/regional/national. |
|  | 2) Selection of the non exposed cohort  a) drawn from the same community as the exposed cohort *****  b) drawn from a different source  c) no description of the derivation of the non-exposed cohort |  |
|  | 3) Ascertainment of exposure  a) secure record (eg surgical records) *****  b) structured interview *****  c) written self report  d) no description |  |
|  | 4) Demonstration that outcome of interest was not present at start of study  a) yes *****  b) no | Assumption: This was taken to mean whether there was discussion of history of disease prior to/at start of study. If there was a star was given. |
| Comparability | 1) Comparability of cohorts on the basis of the design or analysis  a) study controls for age/sex/comorbidity *  b) study controls for any additional factor ***** | 2 * maximum allotted for this criteria.  Assumption: For studies in which hospital associated cases and LoS were being analysed, two stars were only given if time dependency and other potential covariates was controlled for.  If age and sex were excluded for a specific justified reason and other factors included, then this was also awarded a star. If time dependency was not an issue and there were justifications for included factor then the study could be awarded two stars also. |
| Outcome | 1) Assessment of outcome  a) independent blind assessment *****  b) record linkage *****  c) self report  d) no description |  |
|  | 2)Was follow-up long enough for outcomes to occur  a) yes (select an adequate follow up period for outcome of interest) *  b) no | Assumption: This was placed at >=30 days for mortality. |
|  | 3) Adequacy of follow up of cohorts  a) complete follow up - all subjects accounted for *  b) subjects lost to follow up unlikely to introduce bias, small number lost (<20%) or description provided of those lost *  c) follow up rate < 80 % and no description of those lost  d) no statement | Assumption: No description of data cleaning, patient exclusion, sample derivation, or loss to missing data for retrospective studies was not awarding a star. |

1. Economic Modelling Studies

A recent systematic review on the quality assessment tools that can be used when assessing health economic evaluations stated that where it is not a cost-effectiveness study being assessed, but rather economic modelling in general the Philips checklist [3] can be employed. Note that this method has also been recommended by Cochrane for modelling studies [4]. Table 3 represents the Philips checklist utilised in this systematic review [3].

**Table 3: Quality Assessment for Modelling Studies**

| Domains & Dimension of Quality | | Questions for critical appraisal |
| --- | --- | --- |
| Structure (S) | S1 | Is there a clear statement of the decision problem? * |
|  |  | Is the objective of the evaluation and model specified and consistent with the stated decision problem? * |
|  |  | Is the primary decision maker specified? * |
|  | S2 | Is the perspective of the model stated clearly? * |
|  |  | Are the model inputs consistent with the stated perspective? * |
|  |  | Has the scope of the model been stated and justified? * |
|  |  | Are the outcomes of the model consistent with the perspective, scope and overall objective of the model? * |
|  | S3 | Has the evidence regarding the model structure been described? * |
|  |  | Is the structure of the model consistent with a coherent of the health condition under evaluation? * |
|  |  | Have any competing theories regarding model structure been considered? * |
|  |  | Are the sources of data used to develop the structure of the model specified? * |
|  |  | Are the causal relationships described by the model structure justified appropriately * |
|  | S4 | Are the structural assumptions transparent and justified? * |
|  |  | Are the structural assumptions reasonable given the overall objective, perspective and scope of the model? * |
|  | S5 | Is there a clear definition of the options under evaluation? * |
|  |  | Have all feasible and practical options been evaluated? * |
|  |  | Is there justification for the exclusion of feasible options? * |
|  | S6 | Is the chosen model type appropriate given decision problem and specified causal relationships within the model? * |
|  | S7 | Is the time horizon of the model sufficient to reflect all important differences between options? Is the time horizon of the model, and the duration of treatment and treatment effect described and justified? * |
|  |  | Has a lifetime horizon been used? If not, has a shorter time horizon been justified? * |
|  | S8 | Do the disease states (state transition model) or the pathways (decision tree model) reflect the underlying biological process of the disease in question and the impact of interventions? * |
|  | S9 | Is the cycle length defined and justified in term of the natural history of disease? * |
| Data (D) | D1 | Are the data identification methods transparent and appropriate given the objectives of the model? * |
|  |  | Where choices have been made between data sources, are these justified appropriately? * |
|  |  | Has particular attention been paid to identifying data for the important parameters in the model? * |
|  |  | Has the process of selecting key parameters been justified and systematic methods used to identify the most appropriate data? * |
|  |  | Has the quality of the data been assessed appropriately? * |
|  |  | Where epert opinion has been used are the methods described and justified? * |
|  | D2 | Are the pre-model data analysis methodology based on justifiable statistical and epidemiological techniques? * |
|  | D2a | Is the choice of baseline data described and justified? * |
|  |  | Are transition probabilities calculated appropriately? * |
|  |  | Has a half cycle correction been applied to both cost and outcome? * |
|  |  | If not, has this omission been justified? * |
|  | D2b | If relative treatment effects have been from trial data, have they been synthesised using appropriate techniques? * |
|  |  | Have the methods and assumptions used to extrapolate short-term results to final outcomes been documented and justified? * Have alternative assumptions been explored through sensitivity analysis? * |
|  |  | Have assumptions regarding the continuing effect of treatment once treatment is complete been documented and justified?*  Have alternative assumptions been explored through the sensitivity? * |
|  | D2c | Are the utilities incorporated into the model appropriate? * |
|  |  | Is the source for utility weights referenced? * |
|  |  | Are the methods of derivation for the utility weights justified? * |
|  | D3 | Have all data incorporated into the model been described and referenced in sufficient detail? * |
|  |  | Has the use of mutually inconsistent data been justified (i.e. are assumptions and choices appropriate)? * |
|  |  | Is the process of data incorporation transparent? * |
|  |  | If data have been incorporated as distributions, has the choice of distribution for each parameter been described and justified? * |
|  |  | If data have been incorporated as distributions, is it clear that second order uncertainty is reflected? * |
|  | D4: Assessment of uncertainty | Have the four principal type of uncertainty been addressed? * |
|  |  | If not, has the omission been justified? * |
|  | D4a: methodological | Have methodological uncertainties been addressed by running alternative versions of the model with different methodological assumptions? * |
|  | D4b: structural | Is there evidence that structural uncertainties have been addressed via sensitivity analysis? * |
|  | D4c: heterogeneity | Has heterogeneity been dealt with by running the model separately for different sub-groups? * |
|  | D4d: parameter | Are the methods of assessment of parameter uncertainty appropriate? * |
|  |  | Has probabilistic sensitivity analysis been done, if not has this been justified? * |
|  |  | If data are incorporated as point estimates, are the ranges used for sensitivity analysis stated clearly and justified? * |
| Consistency (C) | C1 | Is there evidence that the mathematical logic of the model has been tested thoroughly before use? * |
|  | C2 | Are the conclusions valid given the data presented? * |
|  |  | Are any counterintuitive results explained and justified? * |
|  |  | If the model has been calibrated against independent data, have any differences been explained and justified? * |
|  |  | Have the results of the model been compared with those of previous models and any differences in results explained? * |

**Table 4: Individual Study Characteristics & Quality Score**

Please note that the studies presented in this table are to be considered cohort studies unless “**Case-Control Study:**” or “**Modelling Study:**” have been added to the beginning of the “**Methods**” column. These classifications for each study were utilised using the relevant defined checklists in Tables 1-3 above. One study was labelled as a “Qualitative study” and was not quality assessed [5]. * Population of interest was assumed to be “All” of the patients in the study with the exposure/non-exposure of interest if a particular subgroup was not specified.† These studies either mentioned taking place during or near an outbreak.

| **Study** | **Resistance Related Exposure(s) of Interest** | **Non-exposure definition** | **Outcome of Interest** | **Country Setting** | **Population of Interest*** | **Data Setting** | **Methods** | **Sample Size (resistance related)** | **Result (resistance-related)** | **Stated Limitations** | **Quality Score** |
| --- | --- | --- | --- | --- | --- | --- | --- | --- | --- | --- | --- |
| [6] | *Escherichia coli* bacteremia (cephalosporin resistant and ciprofloxacin resistance separately) | *E. coli* bacteremia (cephalosporin susceptible and ciprofloxacin susceptible separately) | 30-day all-cause mortality | England | All | National surveillance database | Multivariate logistic regression | Out of 28,616 cases; 18 479, 18 256 and 20 475 patients had cephalosporin, carbapenem and ciprofloxacin susceptibility test results, of which 1838 (10.0%), 24 (0.13%) and 3647 (%) were non-susceptible respectively | Ciprofloxacin non-susceptibility increased the odds of death [OR= 1.30 (95% CI 1.15–1.46)]. Carbapenem non-susceptibility was not included in the adjusted model. Cephalosporin non-susceptibility was not associated with mortality in the adjusted model [OR= 1.08 (95% CI 0.93–1.26)] | Missing susceptibility data for many patients and missing data on potentially important factors | 0.89 |
| [7] | Methicillin resistant *Staphylococcus aureus* bacteriuria (MRSA) | Methicillin susceptible *Staphylococcus aureus* bacteriuria (MSSA) | 12-month mortality | USA | All | 1 medical centre | Multivariate logistic regression | 157 MRSA cases, 119 MSSA cases, 50 cases not tested | MRSA was not associated with mortality (in the univariate analysis) with p=0.107 | Retrospective nature, missing data on resistance for some patients and not generalizable due to age and sex bias of population | 0.67 |
| [8] | MRSA infections | MSSA infections and no Staphylococcus aureus infection | All-cause mortality | Global | Post-cardiothoracic surgery patients | Global | Significance tests | 3712 patients without Staphylococcus aureus infection, 97 MSSA infections (MSSA) infections and 23 MRSA infections | The all-cause mortality rate was 4.1% in patients without infection of interest, 7.2% in MSSA infections and 17.4% in MRSA infections (p< 0.01) | Post hoc investigation of data previously collected for another study & missing data | 0.67 |
| [9] | Vancomycin MIC >=2 in MRSA infections | Vancomycin MIC of 1.5 - 2 and of <1.5 in MRSA infections | Mortality | Iran | All | 1 hospital | Descriptive statistics | 18 cases were MIC < 1.5, 17 were MIC 1.5 - 2 and 12 were MIC >2 | Mortality was not significantly associated with a higher MIC (p>0.05) | Not clearly stated | 0.33 |
| [10] | carbapenem resistant *Klebsiella pneumoniae* Gram-negative rod BSI | non-"carbapenem-resistant *K. pneumoniae* " Gram-negative rod BSI | 7-day case fatality rate | Israel | All | 1 hospital | Significance test | 431 CRKP carriers, 19 CRKP BSI cases and 68 GNR BSI cases | The 7-day case fatality rate was 24.4% for patients with GNR BSI excluding CRKP, and 38.9% for patients with CRKP bacteraemia (p = 0.26) | Small power and the group was mainly severely ill patients | 0.56 |
| [11] | General resistance | No resistance | Secondary Effects; World GDP impact (and monetary GDP impact) | International (Global) | Caesarean, join replacement, cancer and organ transplant patients | Not stated | **Modelling Study:** Stepwise calculation (not clear) | N/A | It is estimated that caesarean sections, cancer drugs, organ transplants and joint replacements contribute to around 4% of GDP, worth around 120 trillion USD, some of this could be lost due to resistance. | None clearly stated in relation to this finding in the report. | 0.02 |
| [12] | Carbapenem-resistant Gram-negative bacteremia | Carbapenem-susceptible Gram-negative bacteremia | 14-day mortality and 1 year mortality | Israel | Haematological cancer patients | 1 hospital centre | Logistic regression for 14 day mortality and cox regression analysis for 1 year survival | 320 carbapenem susceptible episodes and 103 carbapenem resistant episodes | Carbapenem resistance was significantly associated with 14-day mortality [OR= 5.14 95% CI; 2.32–11.38 ,p <0.001] and 1-year mortality [HR = 1.48, 95% CI; 1–2.2, p=0.05] | Potential missing factors in adjustment for other covariates | 0.78 |
| [13] | Drug resistant Tuberculosis | Drug susceptible Tuberculosis | Mortality during treatment | Thailand | All | National and regional surveillance data | Cox regression | 7684 pan-susceptible cases, 358 Rifampicin resistant cases, 691 Isoniazid monoresistant cases and 785 Ethambutol/streptomycin resistant cases | There was significantly higher mortality in rifampicin resistance [HR = 1.9, 95% CI; 1.5-2.5) and isoniazid monoresistance [HR 1.4 (95% CI; 1.1-1.7)] than in susceptible cases, in the adjusted models. Resistance was not significant in the adjusted models [HR = 1.0 (95% CI; 0.8-1.3)]. | Use of secondary data & heterogeneity in treatment of patients | 0.78 |
| [14] | Carbapenem-resistant device associated healthcare acquired infections in an ICU | No device associated healthcare acquired infections in an ICU | Crude ICU mortality and crude-excess mortality | Greece | All | 3 hospital ICUs | Significance tests | 294 total included. | Crude ICU mortality for ventilator associated pneumonia (VAP) due to carbapenem-resistant *Acinetobacter baumannii* and carbapenem-resistant *K. pneumoniae* was significantly higher than for patients without HAI in the ICU [RR= 2.50 (95% CI; 1.29-4.82), p = 0.006]. The respective results for catheter-associated BSI are as follows; only carbapenem-resistant *A. baumannii* [RR= 2.12 (95% CI, 1.21-3.70), 0.008] and for patients with only catheter-associated BSI due to carbapenem-resistant *K. pneumoniae* 3.7% [RR =2.50 (95% CI, 1.29-4.82) p =0.006] | Generalisability issue | 0.44 |
| [15] | Carbapenem resistant Gram-negative bloodstream infections | Non-"Carbapenem resistant" Gram-negative bloodstream infections | Excess mortality, length of stay and antibiotic cost | Greece | Adult patients | 2 ICUs | Significance tests | Not clear for resistance comparisons | CR-Gram-negative cases had a relative risk of experiencing mortality of 2.32 [(95% CI; 1.43 - 3.73), p=0.0005], compared to "none infecting organisms". Non-CR-Gram-negative cases had a relative risk of experiencing mortality of 1.87 [(95% CI; 1.19 - 2.93), p=0.003], compared to "none infecting organisms". The medians' difference of length of stay for these comparisons were 14 [(95% CI; 9 - 19), p<0.001], and 10 [(7.5 - 12.5), p<0.001], respectively. The medians' difference in costs for these comparisons were 2,760 EUR (95% CI; 1,492 - 2,570 EUR), p<0.001, and 1,896 EUR (95% CI; 1,222 - 2,570 EUR), p<0.001. (cost date not specified but assumed 2010 EUR) | Small sample size & generalisability | 0.22 |
| [16] | Imipenem resistant MBL positive *Pseudomonas aeruginosa* nosocomial infections | Imipenem resistant MBL negative-*P. aeruginosa* nosocomial infections | Crude mortality and mean ICU duration | India | All patients | 1 hospital | Significance tests | 24 Imipenem resistant MBL positive *P. aeruginosa* isolates and 10 Imipenem resistant MBL negative isolates | Crude mortality was not significantly associated with MBL-positivity (42.86% [6/14] vs. 20% [2/10], p = 0.49), but mean ICU duration was (3.167 ± 0.98 days vs. 16 ± 2.82, p < 0.001) | Potential issues with case definitions (and exclusion of polymicrobial infections) | 0.33 |
| [17] | MDR sepsis and septic shock | non-MDR sepsis and septic shock | Mortality rate | Turkey | All | 1 hospital ICU | Significance tests | 34 MDR cases, 139 total cases | MDR was significantly (p<0.01) associated with a higher mortality rate (70.6% vs 29.4% for non-MDR cases respectively) | Retrospective nature, limited number of patients due to missing data | 0.44 |
| [18] | MRSA Healthcare associated BSIs | non-"MRSA" Healthcare associated BSIs | In-hospital mortality and length of stay (LoS) | Australia | All | 9 hospitals | Multistate modelling | 5847 total HAI BSIs (189 MRSA) | Resistance (MRSA) was associated with increased mortality [HR=4.6 (95% CI; 2.7 to 7.6)], resistance was also associated with excess LoS if discharged alive [12.8 days (95% CI 6.2 to 26.1 days)] | Missing data | 0.67 |
| [19] | Ampicilin resistant *E. coli* bloodstream infection | Ampicilin susceptible *E. coli* bloodstream infection | Main outcome is 30-day mortality and secondary outcomes include 7-day mortality and mortality at discharge | USA | Infants | 77 neonatal ICUs | Multivariate logistic regression | 123 resistant cases, 135 controls | Resistance was not significantly associated with 30-day mortality [OR= 1.37 (95% CI; 0.39,4.77)] and was not significantly associated with other outcomes including 7-day mortality [OR=1.25 (95% CI; 0.35, 4.39)] and in-hospital mortality [OR=1.74 (95% CI; 0.65, 4.67)] | Missing data on potential factors and not powered for mortality analysis | 0.67 |
| [20] | Carbapenem-Resistant *Klebsiella* bacteraemia | Non-"Carbapenem-Resistant" *Klebsiella* bacteraemia | 30-day mortality | USA | Adult patients treated with carbapenems | 2 hospitals | Logistic regression | 7 carbapenem resistance cases, 2017 total | Carbapenem non-susceptibility was significantly associated with 30-day mortality [OR = 9.08 (95% CI, 1.17–70.51) p = 0.04] | Retrospective observational nature and small sample size | 0.78 |
| [21] | MDR-UTIs | Non-MDR UTIs | Relapsing and recurring UTIs | Spain | All post-kidney transplant patients | 1 hospital | Variable significance tests and multivariate logistic regression. Multivariate analysis for Odds Ratios | 119 non-recurrent UTIs, 55 recurrent UTIs (54 first or second episodes caused by MDR pathogens) | MDR (associated with first or second UTI episodes) was associated with recurrent UTI [OR= 12(95 CI; 5 - 28)] and relapsing episodes [OR 3.8 (95% CI; 1.8 - 83)] | Single centre and some data incomplete (no data for some major causative pathogens) | 0.67 |
| [22] | ART pre-treatment resistant HIV | Non-"ART pre-treatment resistant" HIV | All-cause mortality | Global (Pan-African) | Adult | International regional network | Cox regression | 12 pre-treatment drug resistant cases, 176 non-pre-treatment drug resistant cases | Pre-treatment resistance was not significantly associated with mortality [HR=0.75 (95% CI; 0.24–2.35) p = 0.617] | Participation bias, attrition bias and misclassification of non-resistant cases | 0.89 |
| [23] | Healthcare associated vancomycin-resistant *Enterococcus* (VRE) and MRSA infections | Non-"VRE " and non-"MRSA" infections | In-hospital mortality | Italy | Adult patients staying in hospital longer than 48 hours | 1 hospital | Significance tests | There were 234 alive cases and 55 dead cases total, there were 41 MRSA cases and 11 VRE cases | MRSA and vancomycin-resistant *Enterococcus* were not significantly more likely to be found in dead patients compared to alive patients (p>0.05) | Missing information on potentially important factors | 0.44 |
| [24] | MRSA post-partum breast abscess | MSSA post-partum breast abscess | Direct medical cost and healthcare utilization | USA | Post-partum women | 1 hospital | Matching and Significance tests | 30 MRSA cases and 24 MSSA controls | Health services utilization was similar among case patients with MRSA and MSSA, however, MRSA cases had significantly more outpatient visits (median 6.0 versus 3.0). There was no significant cost difference between patients with MRSA infection and those with MSSA. Attributable costs (in 2012 USD) were similar regardless of methodology used - Mean Attributable Hospital Direct Costs= $507 (95% CI; -818, 1842), p= 0.45 for MRSA vs MSSA. [Results also presented using a Medicare unit cost and partial costing, none significant] | Single centre, large cluster of specific MRSA type during study, some lost to follow up | 0.78 |
| [25] | Colistin resistant carbapenem-resistant *K. pneumoniae* | Non-"Colistin resistant" carbapenem-resistant *K. pneumoniae* | In-hospital mortality | Italy | All | 9 hospitals | Binary Logistic regression | 97 CRKP cases | Colistin resistance was found to be an independent risk factor of in-hospital mortality [OR=4.15 (95% CI; 1.17–14.74) p=0.02] | Small sample size and heterogeneity of infections | 0.67 |
| [26] | Heterogeneous vancomycin-intermediate MRSA bloodstream infection | Vancomycin susceptible MRSA bloodstream infection | 30-day mortality, MRSA-infection related mortality and LoS (total and after onset) | USA | >=18 years old | Multicentre (5 centres), National | Matching, multivariate analysis (variables chosen from univariate analysis). Significance tests for LoS. | 61 cases (hVISA MRSA) and 61 controls (vancomycin susceptible MRSA) | Thirty-day non-hVISA MRSA infection-related mortality was not significantly different to hVISA cases (p=0.081), also all-cause 30-day mortality was not significantly different (p=0.076). hVISA was significantly associated with longer total hospital LoS (median difference of 8 days, p = 0.022) and longer LoS after the onset of infection (median difference of 9 days, p=0.021) | Potential case issues from testing methods, relatively small sample size and potential selection bias | 0.56 |
| [27] | High vancomycin minimum inhibitory concentration MSSA bacteraemia | Low vancomycin minimum inhibitory concentration MSSA bacteraemia | 30-day all-cause mortality | Spain | All | 1 hospital | Logistic Regression analysis | 21 of 53 (39.6%) strains had vancomycin MICs= 2 μ g/ml. | High vancomycin MIC [OR= 9.3 (95% CI; 1.31 – 63.20) p= 0.027] was associated with mortality | Retrospective nature and missing data on a potentially important factor (genetic testing) | 0.67 |
| [28] | Multiple species | Non-resistant infections | Resistant infection related deaths | USA | All | Surveillance, literature, expert opinion | **Modelling study:** Synthesis of evidence from different sources on the pathogens and resistance and stepwise calculation | N/A | Estimates the minimum number of illnesses and deaths caused by antibiotic resistance to be 2,049,442 and 23,000 respectively annually | The data presented in this report are approximations, and totals as provided in the national summary tables can provide only a rough estimate due to data limitations | 0.19 |
| [29] | Oseltamivir-resistant seasonal A(H1N1) Influenza | Susceptible seasonal A(H1N1) Influenza | mortality and median length of stay | Hong Kong | >= 18 years | 2 hospitals | Significance tests | 46 resistant cases and 31 non-resistant | The two groups had similar mortality (11–13%) and duration of hospitalization (median 5–6 days) indicators (p=1.000 and p=0.224 respectively). | Small sample size | 0.33 |
| [30] | General resistance in bacteremia | Susceptible bacteremia | Hospital costs (including drug and laboratory cost), LoS, mortality, admissions and complication | India | All | 1 hospital | Variable significance tests | 133 resistant, 87 susceptible cases | Resistance significantly impacted cost in the three main categories – overall cost (88,686 vs 47,380 INR, p=0.001), antibiotic cost (p<0.001), and total pharmacy cost (p<0.001) Intensive care admissions (p<0.001), complications (p=0.006), and mortality (0.011) were significantly higher in the resistant group. Resistance also impacted median LoS (14 vs 11 median days for resistant compared to susceptible respectively, p=0.027). | None stated clearly | 0.56 |
| [31] | Third-generation cephalosporin-resistant Spontaneous bacterial peritonitis | Non-"Third-generation cephalosporin-resistant" Spontaneous bacterial peritonitis | 30-day mortality | Canada | Adult cirrhosis patients | 1 hospital | Logistic regression | 15 resistant cases, 192 cases total | In multivariate analysis, resistance to third-generation cephalosporins was significantly associated with 30-day mortality [OR=5.3 (95 % CI; 1.3 to 22), p=0.02] | Retrospective, small sample size & generalisability | 0.78 |
| [32] | Vancomycin-resistant Enterococcus (VRE) Bacteremia | Vancomycin-susceptible Enterococcus (VSE) Bacteremia | In-hospital mortality, LoS and hospital cost | Australia | Non-pregnant over 18 year olds | 2 hospitals | Matched regression | 116 cases, 116 controls | Resistance was not an independent risk factor for mortality [OR= 1.21 (95% CI; 0.53–2.79)]. Vancomycin resistance increased LoS (4.89 days; 95% CI, 0.56–11.52) and hospitalization costs (AU$ 28 872; 95% CI, 734–70 667) | Potential issues with independent factors in model | 0.56 |
| [33] | Resistant ventilator-associated bacterial pneumonia | Susceptible ventilator-associated bacterial pneumonia | Mortality | Thailand | All | 1 hospital surgical ICU | Cox regression | 150 total patients | Resistance was found to be a significant factor for mortality; including 'resistant organisms' [HR = 1.93 (95% CI; 1.04–3.59) p= 0.04] and XDR [HR= 2.22 (95% CI; 1.16–4.27) p=0.02], but not MDR [HR=1.39 (95% CI; 0.59–3.31), p= 0.45] | Potential external factors influencing result not accounted for (such as bundle intervention) | 0.67 |
| [34] | VRE Bacteremia | VSE Bacteremia | Attributable mortality and 30-day crude mortality | South Korea | >=19 year old patients with neutropenia after chemotherapy or stem cell transplantation (SCT) for underlying hematologic diseases | 1 hospital | Cox proportional hazards regression for mortality and Kaplan-Meier for survival analysis | 24 VRE bacteraemia and 67 VSE bacteraemia cases | Resistance not significantly associated with attributable mortality (p=0.447). In the multivariate analysis, vancomycin resistance was not significantly associated with 30-day mortality [HR= 0.75 (95 CI; 0.24 - 2.36)] | Retrospective, small sample size and potential issues with case definition | 0.67 |
| [35] | Methicillin resistant Septic failures | Methicillin susceptible Septic failures | Mortality | USA | All patients post total knee arthroplasty | 1 hospital | Logistic regression | 26 resistant cases, 62 susceptible cases | Resistance was not significantly associated with mortality (p=0.52, OR not presented) | Retrospective nature, potential database issues and potential causality issues | 0.56 |
| [36] | Carbapenem- and ampicillinsulbactam- resistant *A*. *baumannii* bloodstream infections | Carbapenem- and ampicillinsulbactam- susceptible *A. baumannii* bloodstream infections | In-hospital mortality | USA | All | 8 hospitals | Logistic regression | 68 “Carbapenem- and ampicillinsulbactam- resistant” cases and 206 non-“ Carbapenem- and ampicillinsulbactam- resistant” cases | Resistance was not significantly associated with in-hospital mortality [OR= 1.15 (95% CI; 0.51 to 2.63),p=0.74] | No clear stated limitations | 0.67 |
| [37] | Multi-drug resistant tuberculosis | Non-multi-drug resistant tuberculosis | Mortality | Peru | Adult patients | Regional network | Cox proportional hazards regression | 236 MDR cases, 1232 total | MDR-TB was significantly associated with increased mortality in the crude model (p < 0.001) and was significant when adding other potential covariates [HR = 7.5 (4.1-13.4)] | Potential bias with category definitions (timing) and incomplete data (susceptibility for non-MDR TB cases) | 0.78 |
| [38] | Carbapenem-resistant *Acinetobacter nosocomialis* and *Acinetobacter pittii*, which belong to *Acinetobacter calcoaceticus-A. baumannii*  complex | Carbapenem-susceptible *Acinetobacter nosocomialis* and *Acinetobacter pittii* | 30-day mortality | Thailand | >=18 years old | 1 hospital | Cox proportional hazard regression | 139 Carbapenem intermediate or resistant *A. baumannii* cases, 58 Carbapenem susceptible *A. baumannii* cases | Carbapenem resistance was not significantly associated with 30-day mortality [HR=1.57 (95% CI; 0.89-2.79) p = 0.105] | Retrospective, single centre and potential testing bias | 0.67 |
| [39] | Macrolide resistant community acquired *Streptococcus pnuemoniae* pneumonia | Macrolide susceptible community acquired *Streptococcus pnuemoniae* pneumonia | LoS, 30-day in-hospital mortality, clinical outcomes (e.g. acute renal failure, shock) | Spain | Adults | 1 hospital | Significance test | 135 resistant cases, 504 sensitive cases | Resistance was not significantly associated with LoS [8 vs 8 median days, p=0.96], 30-day mortality (p=0.59) or clinical outcomes such as acute renal failure (0.84). However, resistant cases were significantly less likely to have bacteraemia (p=0.009) or shock (p=0.05) | Generalisability issue, small sample size of resistant cases, lack of information for some factors | 0.56 |
| [40] | MRSA bacteraemia | Non-"Methicillin-Resistant" *Staphylococcus aureus* bacteraemia | 30-day mortality | Spain | Paediatric patients | 1 hospital | Cox regression | 269 episodes of Staphylococcus bacteraemia, 21 MRSA strains isolated | MRSA was not significantly associated with mortality in the univariate analysis [OR=0.51 (95% CI; 0.25–1.07) p=0.07] and so was not included in the multivariate analysis | Retrospective and single centre | 0.67 |
| [41] | Resistant intra-abdominal infection | Susceptible intra-abdominal infection | Mortality, ICU admission | International | Adult patients - post-surgery or treatment for intra-abdominal infection | Multicentre, International  - 116 medical institutions worldwide | Chi squared tests and logistic regression | 21 resistant cases, 546 controls | Resistance was not significantly associated with mortality [Gram positive resistance p=0.115, Gram negative resistance p=0.845] or ICU admission [Gram negative resistance p=0.047, Gram positive resistance p=0.436] | Data did not allow for certain patient classifications or investigation | 0.56 |
| [42] | MRSA foot infections | Non-"MRSA" foot infections | Median antibiotic course and hospital duration, and antibiotic cost per person | Australia | Adult diabetic patients | 1 hospital | Significance tests | 21 MRSA cases, 156 non-MRSA cases | Median length of stay was significantly longer in MRSA infections than non-MRSA infections [68 (IQR; 32.5 -136) days versus 26 (IQR; 10.25 - 42) days respectively, p<0.001]. Median antibiotic course duration was significantly longer in MRSA infections than non-MRSA infections [68 (IQR; 42- 141.5) days versus 30.5 (IQR; 15 - 58.75) days respectively, p<0.001]. Median antibiotic cost per person was significantly higher in MRSA infections than non-MRSA infections [3431 (IQR; 1186 - 5334) AU$ versus 896 (IQR; 380 - 1736) AU$ respectively, p<0.001] (AU$ assumed 2013) | Generalisability | 0.44 |
| [43] | MRSA bacteraemia | MSSA bacteraemia | 30-day mortality | Australia | All | National network | Significance tests | 334 MRSA cases, 1378 MSSA cases | MRSA cases experienced mortality at a significantly higher rate than susceptible cases (p < 0.0001). | Not clearly stated | 0.67 |
| [44] | MDR bacterial hospital acquired infections | Susceptible bacterial hospital acquired infections | Attributable mortality and hospitalisation days | Mexico | Adult cancer patients | ICU in 1 hospital | Variable significance tests and ORs | 134 total patients, 105 MDR isolates | MDR was associated with increased hospitalisation stay in days [16+/- 10 vs. 12 +/-6 days, p = 0.02] and mortality [p=0.05] | None stated clearly | 0.44 |
| [45] | Carbapenem-resistant *K. pneumoniae* | Carbapenem-susceptible *K. pneumoniae* | In-hospital mortality | Brazil | All | 1 hospital | Univariate analysis for ORs & Matching | 20 resistant cases, 236 total cases | Resistance was not significantly associated with mortality [OR=2.64 (95% CI; 0.86-8.07) p=0.085] | Generalisability issue, small sample size and potential unknown factors (colonisation) | 0.67 |
| [46] | Fluconazole resistant Candida species blood stream infections | Fluconazole susceptible Candida species blood stream infections | In-hospital and 14-day mortality | Columbia | All | 7 hospitals | Logistic regression | 125 total, 4 resistant according to 2008 breakpoints and 22 according to 2012 breakpoints | (In-hospital assumed) mortality was not significantly impacted by resistance according to 2008 or 2012 MIC breakpoints [OR=0.54 (95% CI; 0.03–7.6) and OR=1.65 (95% CI; 0.53–5.15)] | Generalisability | 0.56 |
| [47] | XDR Tuberculosis | Rifampicin-mono-resistant and/or MDR Tuberculosis | Monetary cost per patient | South Africa | All | Community programme | **Modelling Study:** Stepwise calculations - Direct medical costs/ outcomes from patient records, applied to unit costs | 467 Rifampicin-resistant TB cases, in which there were 101 mono-resistant, 309 MDR and 20 XDR cases | The mean cost (2013, USD) per patient was 7,916 (Range 260 - 87,140). For XDR-TB and pre-XDR TB this mean cost per patient was 2.5 times higher than for Rifampicin-resistant-mono resistance and MDR (group estimate as 15,567 mean cost (range 1472 - 87,140)) | Missing data on some outcomes and procedures | 0.33 |
| [48] † | New Delhi metallo-β-lactamase (NDM-1) gram negative infection | All non-exposure patients | LoS and in-hospital mortality | South Africa | All | 1 hospital | Bivariate conditional logistic regression analysis was undertaken to calculate crude odds ratio’s for duration of stay. Conditional logistic regression was undertaken to calculate the odds of in-hospital mortality | 38 cases (New Delhi metallo-β-lactamase infection) and 68 controls (other admitted patients) | Cases had a longer mean total length of hospital stay (44.0 vs 13.3 days, P < 0.001), case status was significantly associated with in-hospital mortality, OR = 11.29 (95% CI; 2.57–49.60) p= 0.001 | Small sample size, missing data and potential issues with outbreak investigation | 0.67 |
| [49] | MDR Gram-negative bacterial infection | Non-MDR Gram-negative bacterial infection | 7-day and 30-day mortality | Brazil | <18 years old cancer patients | 1 hospital ICU | **Case-Control:** Logistic regression | 101 cases total, 47 infected by Gram negative bacteria | MDR was not included in multivariate analyses as not found to be significant | Retrospective design and small sample size | 0.89 |
| [50] | Extended-spectrum beta-lactamase (ESBL)-producing *E. coli* bacteraemia | ESBL-negative *E. coli* bacteraemia | 30-day mortality and LoS | France | All | 1 hospital | Logistic regression and significance test for LoS | 41 cases (ESBL+) and 41 controls (ESBL-) | ESBL-positivity was not associated with a longer hospital stay (15 days (IQR=10–21 vs 11 days (IQR= 7–17 days) for ESBL+ vs ESBL- respectively, p = 0.088]. 30-day mortality was not significantly different in the two groups [OR=2.99 (95% CI; 0.59-15.2) p=0.18] | Retrospective nature, single centre and small sample size | 0.67 |
| [51] | MDR tuberculosis | Non-MDR tuberculosis | Direct costs including hospitalization costs, outpatient costs, drug cost and treatment monitoring costs. ( Indirect costs due to loss of productivity on the part of the general economy). | Germany | >=17 years old | National Institute | **Modelling Study:** Evidence synthesis and Stepwise calculations; In patient costs were based on DRG prices. Monte Carlo simulation was performed. Treatment cost was derived from guidelines and prices. Productivity loss was measured using the human capital method. | 65 MDR-TB patients, 4220 TB cases | Mean drug costs were 51,113.22 EUR (range 19,586.14 EUR to 94,767.90 EUR). The weighted costs for hospitalization were 26,000.76 EUR per patient compared to only 2,192.13 EUR for primary outpatients; the total treatment costs of MDR-TB amounted to 64,429.23 EUR. From a societal perspective, the total cost per MDR-TB or XDR-TB case were between 82,150 EUR and 108,733 EUR per case, respectively. | Some data were incomplete | 0.52 |
| [52] | Tuberculosis (MDR and XDR-TB) | Drug-susceptible tuberculosis | Monetary cost; inpatient cost per day, outpatient cost, cost of medication and cost due to lost productivity | Europe | All | Surveillance and literature | **Modelling Study:** Synthesis of evidence from different sources on the pathogens (including a systematic literature review) and resistance and stepwise calculation | N/A | For total economic cost; Average per-TB case cost (in the original EU-15 states plus Cyprus, Malta and Slovenia) 10,282 EUR for drug-susceptible TB, 57,213 EUR for MDR-TB and 170,744 for XDR-TB | Heterogeneity across country delivery of healthcare and data collection | 0.44 |
| [53] | Antimicrobial resistant BSI | Antimicrobial susceptible BSI | Mortality | South Africa | <=14 years old | 1 hospital | Multivariate Logistic regression | 864 BSI episodes | Antimicrobial resistance was not significantly associated with mortality (p = 0.83) | Potential bias with category definitions (e.g. community-associated) and incomplete data (e.g. antimicrobial use) | 0.50 |
| [54] † | KPC-2-producing *K. pneumoniae*  infection | KPC-2-producing *K. pneumoniae*  colonization | Crude hospital mortality and LoS | Greece | All | 1 hospital | Poisson regression for mortality and significance tests for LoS | 35 infected cases and 37 colonized cases | Comparing resistant infection to colonisation - infection had an impact on mortality [IRR = 1.67, (95% CI; 0.99-2.82) p=0.056] and median LoS [57 vs 25 days, p=0.025] | Small observation period for some cases and potential case definition bias | 0.56 |
| [55] | Invasive MRSA infection. | Invasive MSSA infection | In-hospital, 7-day and 30-day mortality | USA | Infants | National network of neonatal ICUs | Modified Poisson regression | 1,110 MRSA cases, 2868 MSSA cases | MRSA did not significantly affect mortality for any of the used mortality measures. Results presented are for in-hospital, 7-day and 30-day mortality respectively; RR= 1.19 (95% CI; 0.96-1.49), RR = 0.90 (95% CI; 0.65-1.24), RR = 1.15 (95% CI; 0.90-1.46) | All-cause mortality used & potential misclassification of non-included cases | 0.89 |
| [56] | Second-line drug (SLI) and fluoroquinolone resistant tuberculosis | SLI and fluoroquinolone susceptible tuberculosis | Mortality and survival | USA | All | National Institute | Cox proportional hazards regression | SLI resistant = 49, SLR susceptible = 2225, fluoroquinolone resistant = 32, susceptible = 1109 | Resistance to SLIs after 8 months of treatment was significantly associated with higher mortality (HR, 2.8; 95% CI, 1.4–5.4). Fluoroquinolone resistance was significantly associated with lower survival (p = 0.03) | Potential lack of generalizability, bias with case definition and lack of data for factors such as comorbidity | 0.89 |
| [57] | ESBL-producing *E. coli* Urinary Tract Infections | ESBL-negative *E. coli* Urinary Tract Infections | Clinical failure and hospital cost per episode | Spain | >= 18 years old | 1 hospital | Matching and Logistic regression | 60 ESBL-producing and 60 non-ESBL producing cases | ESBL was a significant risk factor for clinical failure (p =0.002). ESBL-producing infection cost more than a non-ESBL-producing *E. coli* infection, (mean 4980 EUR vs. 2612 EUR, p=<0.001). Multivariate analysis showed ESBL-production was a significant factor for median cost, OR=3.1 (95% CI; 1.3 - 7.0) p=0.008. | Retrospective nature, only 1:1 matching and did not investigate reimbursement costs | 0.56 |
| [58] | Fluconazole & caspofungin resistant *Candida glabrata* hunfaemia | Fluconazole & caspofungin susceptible *Candida glabrata* hunfaemia | 28-day and in-hospital all-cause mortality | USA | Cancer patients | 1 cancer centre | Log-rank test and Cox regression for 28-day mortality and binary logistic regression for in-hospital mortality | 30 fluconazole resistant cases, 15 caspofungin resistant cases, 146 total cases | Resistance was not significantly associated with 28-day mortality in the multivariate analysis | Retrospective, single centre, missing data for some factors, not powered to detect some factor differences, all-cause mortality rather than attributable as an outcome | 0.56 |
| [59] † | MDR *Acinetobacter calcoaceticus*-*A. baumannii* | Non-MDR *Acinetobacter calcoaceticus*-*A. baumannii* | Main outcome is 30-day mortality and secondary outcomes include 14-day mortality and clinical outcomes such as LoS | USA | >= 18 years old | 1 hospital | Multivariate logistic regression to determine factors for 30-day mortality, significance tests to compare 30-day mortality, 14-day mortality and LoS | 147 ACB complex bacteraemia, 97 MDR | MDR was not significantly associated with 30-day mortality in the multivariate analysis [OR not given], but was associated with increased LoS in univariate comparison [11.5 vs 6, p=0.01]. | Small sample size, retrospective nature, choice of severity index | 0.67 |
| [60] | Resistant Gram-negative bacteremia | Susceptible Gram-negative bacteremia | Mortality (crude and sepsis-related) and LoS | Italy | <18 years old | 1 hospital | Variable significance tests and unadjusted odds ratios | 119 patients, 136 blood isolates; 53 MDR, 33 ESBL, 22 carbapenem resistant | Resistance was significantly associated with sepsis-related mortality [OR= 4.26 (95% CI;1.07-16.9)]. Multidrug resistance was associated with a longer average LoS but this was not significant [90.9 days vs 71.4 days, p-value = 0.093] | Single centre | 0.56 |
| [61] | VRE bloodstream infection | Non-"VRE bloodstream infection" | Mortality & hospital cost | USA | Acute leukaemia patients | 1 hospital (regional referral centre) | Matching and Cox proportional hazards models | 15 resistant cases and 45 controls | Impact on survival was not significant (HR= 1.9 [95% CI, 0.87–5.1], p=0.1). Total median hospital costs were significantly higher for patients with resistant infections ($172,000 vs. $86,000, p= 0.0003), largely due to increased median LoS (42 vs. 29 days, p= 0.0005). (Year of USD unclear, taken as last study year – 2012) | Retrospective nature, institutional practice and molecular typing methodology | 0.56 |
| [62] | VRE blood stream infection | Non-"VRE bloodstream infection" | LoS and hospital cost | USA | Allogeneic hematopoietic stem cell transplantation recipients | 1 hospital | Significance tests - not clear for outcomes | Out of 308 total; No VRE colonisation or BSI = 283, VRE colonisation without BSI= 201, VRE colonisation with BSI =13, VRE BSI without colonisation=1 | No significant differences were seen in 3-month mortality with and without VRE BSI (0% vs. 2.1%, respectively). Median LoS was significantly longer for patients with VRE BSI than for colonised patients without BSI (24 vs. 20.5 days, p = 0.04). Median costs were not significantly higher with VRE BSI ($61,151 vs. $54,992, p=0.34). VRE colonised and non-colonised patients without VRE BSI had no significant difference in LoS and there was no differences in 1-year survival (92% vs. 90% for VRE-positive and VRE-negative patients). [Comparators were not always clear]. | Retrospective nature, single centre and potential testing issues | 0.44 |
| [63] | MDR tuberculosis | Susceptible tuberculosis | Days treated and clinical outcomes (hospitalisation, adverse events, treatment success) | Australia | All | National surveillance system | Matching and significance tests | 16 cases matched to 3 controls each | MDR was associated with longer treatment (mean, 597 v 229 days, no p-value or significance test value given), hospitalisation (p<0.001) and adverse effects (p<0.001). There was no significant difference in treatment success, and there were no deaths. | Small sample size | 0.78 |
| [64] † | Polymyxin-resistant *A. baumannii*  infection | Non-"Polymyxin-resistant *A. baumannii*  infection" | 60-day mortality | Brazil | All liver transplant patients | 1 hospital | Cox proportional hazards regression | 7 cases with Polymyxin-resistant *A. baumannii* , 65 patients in total | Polymyxin-resistant *A. baumannii*  was associated with mortality [HR= 1.03 (95% CI; 1.01–1.05), p=0.03] | None stated clearly | 0.67 |
| [65] | XDR *A. baumannii*  bacteremia | Non-XDR *A. baumannii*  bacteremia | 30-day mortality | China | Elderly | 1 hospital | Variable significance tests and logistic regression | 39 resistance cases, 86 controls | XDR was found to be independently associated with 30-day risk of mortality in hospital [OR = 4.01 (95% CI; 1.46 - 11.04), p=0.01] | Retrospective nature | 0.67 |
| [66] † | MDR & XDR tuberculosis | Drug-susceptible tuberculosis | Mortality | South Africa | Adult patients | 1 hospital | Bivariate and Multivariable analysis using product limit estimates and Cox proportional hazards analysis. To account for missing CD4 counts for multivariable analysis, performed multiple imputation using a Markov Chain Monte Carlo method | 227 patients who contributed 297 TB isolates for this study. Eighty-six (38%) patients had drug-susceptible TB, 67 (30%) had MDR TB; and 74 (33%) had XDR-TB. | MDR TB and XDR TB remained independently associated with mortality [MDR HR= 3.37, p<0.0001; XDR HR =6.75, p<0.0001] | Potential selection bias, potential bias from nearby outbreak | 0.44 |
| [67] | Nosocomial MDR *A. baumannii*  blood stream infection | Nosocomial susceptible *A. baumannii*  blood stream infection | Total hospitalisation cost and antibiotic cost | Turkey | All | General Intensive Care (GICU) and Neurology-Neurosurgery Intensive Care (NNICU) Units in 1 hospital | Variable significance tests, Regression and Kaplan-Meier survival analysis | 41 resistant cases, 45 non-*A. baumannii*  controls | Resistant *A. baumannii*  infection was not significantly associated with a difference between mean total hospitalisation cost compared to sensitive infections (35277 USD vs 20398 USD) and not significantly associated with a difference between mean antibiotic cost (1052 USD vs 836 USD) | None stated clearly | 0.56 |
| [68] | ESBL- producing *K. pneumoniae*  BSI | ESBL-negative *K. pneumoniae*  BSI | In-hospital mortality, total LoS, LoS before BSI onset and LoS after BSI onset (median days) | Germany | All | 1 hospital | Cox proportional hazards model for mortality and significance tests for LoS | 66 ESBL + cases and 286 ESBL - controls | ESBL + did not significantly impact in-hospital mortality (HR=0.629(95% CI; 0.36-1.10), p= 0.101, and HR=0.854 (95% CI; 0.49-1.48), p = 0.573 for two model types). ESBL + did have a significant impact on total LoS, LoS before BSI onset and LoS after onset (ESBL +, ESBL - and p-value respectively); 44(26-82) vs 24(11-46) with p<0.001, 23(2-51) vs 5(0-17) with p<0.001 and 18(9-42) vs 12(7-25) with p=0.027. | Missing potentially important factors from the analysis, such as inappropriate therapy | 0.56 |
| [69] | ESBL-positive *E. coli* bacteraemia | ESBL-negative *E. coli* bacteraemia | 30-day mortality | South Korea | Cancer patients, >= 16 years old | 1 cancer centre | Logistic regression | 95 ESBL-positive, 255 ESBL-negative cases | ESBL production was found to significantly impact mortality in the multivariate analysis [ESBL-*E. coli* bacteraemia [OR = 3.01 (95% CI; 1.45–6.28), p = 0.003].In the multivariate analyses of each subgroup (e.g. patients with solid tumours) ESBL production was consistently found to be an independent risk factor of mortality. | Retrospective and observational nature, missing data on potential factors, generalisability | 0.67 |
| [70] | Resistant Gram-negative bacteraemia | Susceptible Gram-negative bacteraemia | Infection-related mortality and 30-day all-cause mortality, LoS | Australia | Paediatric oncology patients | 1 hospital | Logistic regression for categorical risk factors and outcomes. Significance tests used to estimate P values for continuous risk factors and outcomes. | 42 resistant cases, 238 non-resistant cases | There was a significant increase in the median hospital LOS in episodes of AR GN bacteraemia compared with non-AR GN bacteraemia (23.5 versus 14.0 days, p = 0.0007). There was not a significant increase in infection-related (OR 5.7, 95% CI: 0.3–95.9, p=0.22) and all-cause mortality (OR 1.8, 95% CI: 0.5–6.7, p=0.42) in this group | Retrospective and single centre | 0.67 |
| [71] | ESBL-producing *E. coli* and *K. pneumoniae*  bacteremia | ESBL-negative *E. coli* and *K. pneumoniae*  bacteremia | Crude & attributable mortality | South Korea | Febrile neutropenic patients <20 years old after anticancer chemotherapy or hematopoietic cell transplantation | 1 hospital | Significance tests | 20 ESBL-producing cases and 40 non-ESBL-producing group | Clinical outcomes did not significantly differ between the ESBL-producing and non-ESBL-producing cases (p-value for crude mortality was 0.683 and for attributable mortality was 1) | Potential testing bias, change in treatment between groups | 0.44 |
| [72] | High antibiotic resistance - Multiple types of resistance bacterial infections | Low antibiotic resistance - Multiple types of resistance bacterial infections | ICU and hospital mortality, ICU and hospital LoS | International | Adults | 1265 ICUs from 75 countries | Logistic regression with both matching and propensity score models for mortality, significance tests for LoS | 13796 total patients | Resistance was not found to be associated with mortality in any of the models (p>0.3 for all), in-hospital mortality comparing high antibiotic resistance countries’ infected patients to low antibiotic resistance countries' patients was not significant [OR = 1.1 (95% CI; 0.77-1.58) p=0.606], countries with higher resistance versus countries with lower resistance did have significantly longer LoS outcomes (p<0.001) | Missing data for some factors | 0.56 |
| [73] | Carbapenem resistant community- and health care-associated *P. aeruginosa* bacteremia | Carbapenem susceptible community- and health care-associated *P. aeruginosa* bacteremia | 30-day mortality | USA | All | 2 hospitals | Cox regression hazard model | 150 isolate total, 13 carbapenem resistant | Resistance was associated with a non-significant increase in 30-day mortality [HR=1.53, (95% CI;0.68-3.42),p=0.3] | Retrospective nature and potential impact from confounding factors | 0.44 |
| [74] | Carbapenem-resistant *A. baumannii*  colonization or infection | Non-exposure hospital patients | Mortality | Israel | Adult patients | Network of hospitals, outpatient clinics and long term care facilities | Kaplan-Meier survival analysis and Cox proportional hazards model | 1190 resistant cases and 1190 controls | Resistance was associated with increased mortality [HR = 2.33 (95 % CI; 2.08–2.6),p<0.001]. | Biases associated with matching technique | 0.89 |
| [75] | Third-generation cephalosporin resistant Enterobacteriaceae community-onset bacteremia | Non-exposure community-onset bacteremia | 7-day, 30-day related mortality | Spain | All | 1 hospital | Logistic regression | 44 third-generation cephalosporin resistant Enterobacteriaceae cases out of 745 total cases | Third-generation cephalosporin resistant Enterobacteriaceae had the lowest 7-day mortality compared to all other microorganisms (p= 0.07) or to third-generation cephalosporin susceptible Enterobacteriaceae (p=0.08). For 30-day mortality third-generation cephalosporin resistant Enterobacteriaceae was seen as a protective factor [OR= 0.06, (95% CI; 0.01–0.77)] | Single centre, small sample size and potential issues with case definition/exclusion | 0.78 |
| [76] | Methicillin-resistant and third-generation cephalosporin resistant Enterobacteriaceae community-onset bacteremia | Non-exposure community-onset bacteremia | 30-day mortality | Spain | >= 65 years old | 1 hospital | Multivariate logistic regression | Total sample was 2605, 52 MRSA cases and 166 third-generation cephalosporin resistant Enterobacteriaceae cases | In the multivariable analysis; MRSA had OR= 2.83 (95% CI; 1.38-5.78) and third generation cephalosporin resistant Enterobacteriaceae cases had OR= 1.96 (95% CI; 1.16-3.32) | Single centre, missing data on potentially important factors | 0.67 |
| [77] | MRSA Ventilator Associated Pneumonia | Non-exposure Ventilator Associated Pneumonia | Ventilator days, ICU LoS, hospital LoS, and mortality | USA | All trauma patients | 1 specialist centre | Significance tests for all outcomes except multivariate regression for mortality | 997 Ventilator Associated Pneumonia episodes, number of exposure cases unclear | Resistance was not associated with different outcomes. Resistance was not an independent predictor of mortality [OR= 0.815, (p = 0.59)]. ICU LoS was 24 vs 23 days (p=0.804) and hospital LoS was 34 vs 35 days (p=0.756) for MRSA vs non-MRSA respectively | Generalisability issue | 0.56 |
| [78] | High vancomycin MICs in MRSA bacteraemia | Low vancomycin MICs in MRSA bacteraemia | 28-day mortality | UK | All | 24 hospitals | Mixed-effects logistic regression models | 159 observations in analysis which we cite results from | Death was not significantly associated with higher vancomycin MICs by either method tested, for one method OR = 1.19 [95% CI; 0.22 - 6.39] for 1.4 or 2 MIC compared to 1 MIC. | Poor completion rate for some data | 0.78 |
| [79] | Carbapenem resistant *K. pnuemoniae* (CRKP) | Carbapenem susceptible *K. pnuemoniae* (CSKP) | 30-day crude mortality | Italy | >= 18 years old | 10 hospitals | Matched and Poisson regression | 49 CRKP cases & 49 CSKP cases | Resistance was associated with 30-day mortality [matched IRR = 3.0 (95% CI; 1.3 - 7.1) p=0.012] | Small sample size, national generalisability issue, missing data on some factors | 0.67 |
| [80] | Vancomycin resistant in MRSA bacteraemia | Vancomycin susceptible in MRSA bacteraemia | In-hospital mortality, MRSA bacteraemia related mortality, length of ICU stay, length of hospital stay | Taiwan | >=18 years old | 1 hospital | Logistic regression for in-hospital mortality, significance tests for attributable mortality and LoS outcomes | 14 hVISA, 34 VSSA | hVISA was not significantly associated with additional LoS [length of ICU stay after bacteraemia; 27.7 vs 31.5 mean days, p=0.629, hospital stay 32.6 vs 33.4 mean days (p=0.96) or attributable mortality (p=0.915) for sensitive vs resistant cases respectively]. hVISA was associated with in-hospital mortality [OR= 11.8 (95% CI; 1.1–126.99) p=0.042]. | Retrospective nature, single site and small sample size | 0.67 |
| [81] | High imipenem MIC *E. coli* & *K. pneumoniae* | Low imipenem MIC *E. coli* & *K. pneumoniae* | 30-day mortality | Taiwan | All | 1 hospital | Significance test | 76 cases total | There was no significant difference in the 30-day mortality rate between the group with an imipenem MIC <= 1 mg/mL and the group with an imipenem MIC> 4 mg/mL (p = 0.852). | No clear stated limitations | 0.44 |
| [82] | MRSA colonization or infection | Non-"MRSA colonization or infection" of the same health resource groups | Direct costs - hygienic management costs, laboratory cost and indirect costs - opportunity costs | Germany | All | 1 hospital | **Modelling Study:** Evidence synthesis & Stepwise calculations; internal evidence on financial & consumption data, also national reimbursement figures. Calculations applied based on cases seen at the hospital | 182 MRSA cases | Additional LoS for MRSA cases was 2.46 days, compared with mean length of stay for the same group classification (as defined by resource usage group), resulting in an additional EUR 1,081.53 per case. In total, the opportunity costs were estimated to be EUR 6,717.44 per case. Overall, the MRSA-attributed costs were EUR 8,673.04. | Retrospective, single centre, exclusion of certain costs (e.g. drug costs) and case definition bias (no difference between infected and colonized in study) | 0.48 |
| [83] | Extended-spectrum cephalosporin–resistant Enterobacter species | Extended-spectrum cephalosporin–susceptible Enterobacter species | all-cause 30- day mortality | South Korea | All cancer patients | 1 hospital | Multivariate logistic regression= | 53 resistant cases, 192 total | Resistance was slightly associated with mortality for the entire case group (OR 2.33: 95% CI; 0.95 - 5.74). In *E. arogenes* subgroup resistance was significantly associated with 30-day mortality (OR 5.92; 95% CI; 1.08 - 42.45) | Retrospective nature and generalisability issue | 0.56 |
| [84] | Healthcare acquired - Carbapenem-resistant *K. pneumoniae*  bacteremia | Healthcare acquired - Carbapenem-susceptible *K. pneumoniae*  bacteremia | 30-day mortality | Israel | Adult patients | 1 hospital | Logistic regression | 103 Cases, 214 controls | Resistance was not a significant factor for mortality [OR = 1.9 (95% CI; 1.2-3.1), p=0.01 in univariate and not included in multivariate] | Retrospective nature and other factors not included could be important | 0.78 |
| [85] | MDR, XDR and pan-drug resistant *A. baumannii*  ventilator-associated pneumonia | Drug-susceptible *A. baumannii*  ventilator-associated pneumonia | 30-day mortality | Thailand | Adult | ICU in 1 hospital | Cox proportional hazards regression | 72 MDR, 220 XDR, 12 PDR cases and 33 controls | Resistance was found to not be significantly associated with mortality in the multivariate analysis. [MDR HR=1.03 (95% CI; 0.44-2.45),p=0.936, XDR HR = 1.64 (0.74-3.64), p=0.220, and pan-drug resistant HR = 1.41 (0.46 - 4.23), p=0.537] | Single centre and some data incomplete | 0.67 |
| [86] | community-acquired pneumonia (resistance to empirical antibiotic prescribed) | community-acquired pneumonia (not resistant to empirical antibiotic prescribed) | Mortality and LoS | Nigeria | >= 18 years old | 4 hospitals | Logistic regression - but not clear, significance tests for LoS | 189 total cases analysed | Ceftriaxone resistance was associated with higher mortality [OR =4.5, 95% CI (1.3 – 18.6), p =0.01] and ciprofloxacin resistance was also significantly associated with mortality [OR =2.9, 95% CI (1.1 – 6.5), p= 0.04]. Among the survivors, resistance to coamoxiclav (12.7 (+/-5.1) vs 10.0(+/-3.6) days, p=0.001), ceftriaxone (12.4( +/- 5.0) vs 9.6 (+/-3.7) days, p=0.0006) and ciprofloxacin (12.0 (+/-4.9) vs 10.2 (+/-3.9) days, p =0.001) had a significant impact on LoS. | Retrospective nature and potential issues with testing | 0.67 |
| [87] | Levofloxacin resistance *Streptococcus pneumoniae* pneumococcal infections | Levofloxacin susceptible *Streptococcus pneumoniae* pneumococcal infections | Mortality | Spain | All | 1 hospital | Univariate analysis | 20 resistant cases 102 controls | Mortality not significantly associated with infection caused resistant vs susceptible cases (p=0.092) | None clearly stated | 0.44 |
| [88] | MRSA colonization or infection | MRSA negative | Main outcome is LoS, other clinical outcomes include admission to ICU, median LoS in ICU, death and more specific outcomes such as total number of grafting procedures | Australia | All burn patients | 1 hospital | Significance tests and linear regression model for LoS factors | 57 MRSA positive cases and 300 MRSA negative controls | Resistance was related significantly to all outcomes (p<0.001), apart from death (in which no MRSA positive patients died, p=0.60). MRSA was significantly associated with LoS in the regression analysis [6.0 additional days (95%CI 2.39–9.6), p = 0.001] | Potential bias with case definition (colonisation vs infection) | 0.56 |
| [89] | MDR Gram-negative BSI | Non-MDR Gram-negative BSI | All-cause mortality and sepsis-related mortality | Hungary | <18 years old | 6 hospitals | Logistic regression | 45 MDR cases, 134 total | Multidrug resistance was not significantly associated with all-cause or sepsis-related mortality (p=0.16) | Heterogeneity of samples and sites, missing data on potential factors | 0.67 |
| [90] | Linezolid resistant Enterococci infection | Linezolid susceptible Enterococci infection | In-hospital mortality | China | All | 1 hospital | Univariate OR calculation | 44 non-susceptible cases vs 44 susceptible cases | For non-susceptible vs susceptible cases the OR of in-hospital mortality was 1.54 (95% CI; 0.24-9.68) p>= .999 | Retrospective cohort study with small sample size and no-molecular level resistance found | 0.56 |
| [91] | MRSA BSI | Non-"MRSA BSI" | Direct medical cost | South Korea | Adult patients | 8 hospitals | Stepwise calculations - Direct medical costs from patient records, applied to unit costs from insurance literature (including cost of drugs, tests and procedures) | 335 MRSA BSI patients | Cost per MRSA patient was estimated as South Korean Won 5,435,361 (US$4,906) | Not internationally generalizable and potentially other influencing factors | 0.56 |
| [92] | Levofloxacin resistant community-onset invasive pneumococcal infections | Levofloxacin susceptible community-onset invasive pneumococcal infections | 30-day mortality | International (Asia) | All | International regional network | Logistic regression | 136 invasive pneumococcal infection cases in total | Levofloxacin resistance was significantly associated with 30-day mortality [OR= 14.33 (95% CI; 1.01–203.93), p= 0.049] | Retrospective nature, small sample size for further investigation, potentially other important factors | 0.56 |
| [93] | MDR Salmonella meningitis | Non-MDR Salmonella meningitis | Mortality | South Africa | All (who had HIV status recorded) | National surveillance network | Multivariate logistic regression (forward stepwise selection) | 49 MDR cases, 86 non-MDR cases | MDR was not significantly associated with mortality [OR= 0.6 (95% CI; 0.1-5.5, p=0.648]. | Missing data for patients | 0.67 |
| [94] | ESBL producing *E. coli* & *K. pneumoniae*  bacteraemia | ESBL-negative *E. coli* & *K. pneumoniae*  bacteraemia | 7-day and 30-day mortality | South Korea | >=16 years old | 1 hospital | Logistic regression for 30-day mortality, significance tests for 7-day | 26 ESBL-producing cases, 75 non-ESBL producing controls | From the logistic regression ESBL production was not significantly associated with 30-day mortality [OR= 3.221 (95% CI; 0.745 - 12.3982) p=0.117] | Single centre, small sample size, did not perform microbiologic analysis | 0.67 |
| [95] | Methicillin-resistant and methicillin-susceptible Staphylococcus aureus bacteraemia (hospital-onset) | Non-nosocomial infection | Mortality (at 12 weeks), excess cost (direct medical and societal (included cost to caregivers and lost productivity)) and excess length of stay. | South Korea | Adult patients | National network (hospitals with over 500 beds) | Matching | 133 resistant cases matched to 133 controls and 57 susceptible cases matched to 57 controls | Mortality was not significantly different between MSSA and controls (p<0.052), but was for MRSA compared to controls (p<0.001). The additional medical cost for one case of MRSA was $11,259 in the survivor group and $14,772in the non-survivor group, for MSSA cases these estimates were $4,797 in and $408 respectively. Excess economic burden per case of nosocomial MRSA case was US $20,494 and $6,914 for MSSA case. Comparing median length of stay with controls gave 38.5 (IQR; 26.8) days for MRSA and 25.8 (IQR; 28.0) days for controls (p<0.001). Costs in 2011 USD. | Small sampling period and potential bias in site selection | 0.78 |
| [96] | ESBL-producing and extended-spectrum cephalosporin (ESC) resistant Enterobacteriaceae BSIs | ESBL-negative and/or extended-spectrum cephalosporin-susceptible Enterobacteriaceae BSIs | 30-day mortality | Korea | Cancer patients | 1 hospital | Logistic regression | 31 ESBL positive cases, 100 ESC sensitive/ESBL negative cases, 72 ESC-resistant/ESBL negative cases | ESBL production and ESC was not significant in univariate analysis and was not included in the logistic regression | Single centre, potential testing issues and combining adult and paediatric cases | 0.56 |
| [97] | ESBL-producing *E. coli* & *K. pnuemoniae* spontaneous bacterial peritonitis | ESBL-negative *E. coli* & *K. pnuemoniae* spontaneous bacterial peritonitis | 30-day mortality | South Korea | All | 1 hospital | Cox proportional hazard regression | 52 ESBL-producing cases and 179 non-ESBL-producing cases | ESBL-production was significantly associated with 30-day mortality [HR=1.82 (95% CI; 1.002–3.31) p=0.049] | Misses potentially important co-founders and does not estimate attributable mortality | 0.67 |
| [98] | MRSA Cardiac Implanted Electronic Device-Related Infective Endocarditis | Non-"MRSA" Cardiac Implanted Electronic Device-Related Infective Endocarditis | Mortality | USA | Post-cardiac implant | 1 hospital | Kaplan-Meier and logistic regression for odds ratios | 29 MRSA cases, 80 total cases | MRSA was associated with mortality (p<0.001) in the survival analysis, the logistic regression found MRSA to be an independent predictor of mortality [OR=0.158 (95% CI; 0.047–0.534) p = 0.003] | Retrospective and single centre | 0.78 |
| [99] | Community associated-MRSA bone and joint infection | Community associated-MSSA bone and joint infection | Clinical outcome (e.g. C-reactive protein value), LoS and antibiotic course | India | <18 years old | 1 hospital | Multivariate logistic regression | 41 resistant cases, 33 susceptible cases | Resistance was significantly associated with worse clinical values [C-reactive protein values; p=0.006], longer LoS [16.6 vs 10.5 mean days, p=0.000] and length of antibiotic course [p=0.000] | Retrospective nature, small sample and case definition issue (community associated vs hospital associated hard to define) | 0.67 |
| [100] | XDR/MDR *A. baumannii*  infection | Susceptible *A. baumannii*  infection | 30-day mortality | USA | Adult post abdominal solid organ transplant (SOT) patients | 1 hospital | Variable significance tests and binary logistic regression. | Multidrug-resistant *A. baumannii -* 28 and XDR-*A. baumannii* -7 cases, 33 SOT recipients during the study period with *A. baumannii* infection. | XDR was associated with a greater risk of death [(OR=7.0 ( 95% CI 1.1–44.1), p = 0.047)] | Small sample size and incomplete data (on antimicrobial dosing) | 0.44 |
| [101] | Resistance in multiple species - 3 bacteria & 3 other microbes | Less resistance (different scenarios provided) | Monetary cost and proportion of GDP loss | Global | All | Surveillance, literature, expert opinion | **Modelling Study:** Productivity was modelled using a Total Factor Productivity (TFP) model | N/A | In a scenario with 100% resistance rates, world GDP is projected to be 3.4% lower by 2050 than if there were no resistance. Low-income countries are expected to have the biggest decline, Africa is projected to be the most hit region. The most severe scenario (doubling of current infection rates for the three bacteria, HIV and Tuberculosis, and 100% resistance rate in all countries) is projected to see world GDP over 14,228,000,000,000 USD lower in 2050, with world output projected to be just over 6% lower by 2050 also. | Only a certain number of diseases and only looking at impact on GDP, 'secondary' health care costs not included. Other economic relationships such as trade and cross border transit patterns were not accounted for. Data limitations, particular lack for infection rates in low- and middle-income countries. Future AMR scenarios used assumed no further development of new anti-microbial drugs. | 0.42 |
| [102] | Nalidix, ceftriaxone and multidrug resistant nontyphoidal salmonella infection | Non-exposure nontyphoidal salmonella infection | Death, LoS > 3 days and clinical outcomes such as diarrhoea | USA | All | National surveillance system | Significance tests with group comparison adjustment | 875 total patients | Risk of a hospital stay greater than 3 days was 2 times higher (95% CI 1.3–3.0) for patients with infections resistant to 5 antimicrobial classes, 1.7 times higher (95% CI 1.1–2.7) for those resistant to at least ampicillin, chloramphenicol, streptomycin, sulfisoxazole, and tetracycline ACSSuT, and 1.9 times higher (95% CI 1.2–3.2) for ACSSuT-only resistance. Only 1 patient died so there are no results presented on risk of death. | Small sample size for resistant cases and missing data on potentially important factors | 0.78 |
| [103] | Carbapenem-resistant *A. baumannii* BSI | Carbapenem-susceptible *A. baumannii* BSI | All-cause mortality | India | Neonate patients | 1 hospital | Significance test | 33 resistant cases, 32 susceptible cases | All-cause mortality rate was 27.3% for resistant cases vs 9.4% for susceptible cases, but not statistically significant (p = 0.074). | Retrospective nature and missing data on a potentially important factor | 0.44 |
| [104] | Sulbactam non-susceptible *Acinetobacter nosocomialis* bacteremia | Sulbactam susceptible *A. nosocomialis* bacteremia | 14-day mortality | Taiwan | >=18 years old | 1 hospital | Significance test | 41 resistant cases and 226 susceptible cases | The 14-day mortality was higher in patients with non-susceptible cases of *A. nosocomialis* bacteraemia (17.1% vs. 7.5%, p=0.049) | Retrospective and single centre | 0.44 |
| [105] | Carbapenem resistant Gram-negative culture | Carbapenem susceptible Gram-negative culture | All-cause mortality 90 days post transplantation | Italy | >=18 years old | 44 transplant units | Time-adjusted and multivariate mixed-effects Poisson regression model | 45 resistant cases, 136 susceptible cases | Carbapenem resistance was significantly associated with mortality [IRR= 10.23(95% CI; 4.69–22.31), p<0.001] | National surveillance data, heterogeneity in labs, missing data on potentially important factors | 0.78 |
| [106] | Community associated-MRSA | No "community associated-MRSA" | Third-party payer and productivity cost (including from caregivers) | USA | Adult | Literature | **Modelling Study:** Decision Tree Model | N/A | Third-party payer costs of up to $478 million- $2.2 billion and economic costs of up to $1.4–$13.8 billion annually | General limitations associated with model assumptions and parameter values used | 0.61 |
| [107] | Imipenem-resistant *A. baumannii*  complex bactereamia | Imipenem-susceptible *A. baumannii*  complex bactereamia | 30-day mortality | Taiwan | >=18 | 1 hospital | Logistic regression and Kaplan-Meier analysis | 298 total ACB cases, 73 imipenem resistant cases | Patients with imipenem resistant infections had a higher 30-day mortality rate (70.0%) than those infected by imipenem sensitive (24.5%) (p < 0.001) | No clear stated limitations | 0.56 |
| [108] | Community-acquired MRSA bacteraemia | Community-acquired MSSA bacteraemia | Mortality and LoS | South Korea | >16 years old | 1 hospital | Significance tests | 31 MRSA cases and 138 MSSA cases | MRSA was associated with additional hospital stay; median days (IQR) 47 (19–87) for MRSA and 24 (14–44.5) for MSSA, p= 0.004 MRSA was not associated with overall death, for 30 day mortality OR= 0.43 (95% CI; 0.10–1.96) p=0.37. MRSA was not associated with SAB-related death, for 30 day mortality OR= 0.32 (95% CI; 0.04–2.55) p=0.47. | Retrospective, single centre and small sample size | 0.44 |
| [109] | Imipenem-resistant *A. baumannii*  ST455 causing bloodstream infections | Imipenem-susceptible *A. baumannii*  ST455 causing bloodstream infections | 30-day mortality, attributable mortality | Taiwan | >=18 years old | 1 hospital | Logistic regression for 30-day mortality, backward root analysis for attributable mortality | 189 MRSA cases, 151 vancomycin MIC>=1.5, 163 teicoplanin MIC>=2, 138 daptomycin MIC>=0.38, 121 linezolid MIC>=1.5 cases | For the 30-day mortality model, vancomycin MIC>= 2 was significantly associated with mortality (p<0.001), in the multivariate analysis p=0.004 [OR = 3.49 (95% CI; 1.50–8.14) p=0.004]. In the attributable mortality model, higher vancomycin, teicoplanin and daptomycin MICs were associated with higher mortality (p=0.003, p=0.005, p=0.012 respectively) | Not clearly stated | 0.78 |
| [110] | MRSA bacteraemia - vancomycin, teicoplanin, daptomycin high MICs | MRSA bacteraemia - vancomycin, teicoplanin, daptomycin low MICs | Attributable mortality | Taiwan | ICU adult patients | 1 ICU | Significance tests (for our results of interest - though not clear) | 39 resistant cases, 13 susceptible cases | Imipenem-resistant *A. baumanniii* bacteremia was significantly associated with higher mortality than those with susceptible bacteremia (p= 0.009). | Not clearly stated | 0.33 |
| [111] | MDR pneumonia (multiple species) | Non-MDR pneumonia | ICU mortality, ICU LoS and other clinical outcomes | South Korea | All | 1 hospital | Cox proportional hazards, regression for mortality and univariate analysis for LoS | 22 MDR cases and 102 total | MDR was not significantly associated with mortality, OR=1.39(95% CI; 0.68-2.84, p=0.365). MDR was not significantly associated with LoS with a mean of 18 where MDR not present and 11.8 where MDR present (p=0.308) | Single centre, retrospective, missing data on potential confounding factors (antimicrobial therapy) | 0.67 |
| [112] | ESBL-producing *E. coli* bloodstream infection | ESBL-negative *E. coli* bloodstream infection | Mortality | Germany | All | 1 hospital | Multivariate logistic regression | 115 ESBL-positive cases and 983 ESBL-negative cases | ESBL not significant in multivariate analysis (results not presented) | Missing information on potentially important factors | 0.67 |
| [113] | ESBL-producing *E. coli* bacteraemia | ESBL-negative *E. coli* bacteraemia | In-hospital Mortality, Hospital cost and length of stay | Germany | All | 1 hospital | Linear regression for health system outcomes and significance tests for mortality | 66 ESBL-producing cases out of 352 *Klebsiella pnuemoniae* cases, 178 ESBL-producing cases out of 1,499 *Eschericia coli* cases. | For total hospital cost; *K. pneumoniae* ESBL-negative was the reference point, *K. pneumoniae* ESBL-positive multiplicative effect (ME)= 2.21 (95% CI; 1.63–2.98) p=0.001, *E. coli* ESBL-positive ME=1.11 (0.9–1.36) p= 0.345, *E. coli* ESBL-negative ME=0.79 (0.69–0.92) p=0.002. For total length of stay; *K. pneumoniae* ESBL-negative was the reference point, *K. pneumoniae* ESBL-positive ME = 1.86 (95% CI; 1.46–2.37) p=0.001, *E. coli* ESBL-positive ME= 1.07 (95% CI; 0.9–1.27) p=0.460 and *E. coli* ESBL-negative ME=0.83 (0.74–0.93) p=0.002. Significant impact of ESBL+ on mortality (p=0.006) | Retrospective, did not adjust for surviving patients, missing potentially important factors and did not evaluate possible interaction between predictor variables. | 0.63 |
| [114] | ESBL-producing *K. pneumoniae*  & *E. coli* | ESBL-negative *K. pneumoniae*  & *E. coli* | Mortality, hospital cost and LoS | Germany | All | 1 hospital | Matching | 115 ESBL-positive and 983 ESBL-negative cases | After matching for confounders, there were no significant differences in any outcomes - total hospital costs (15 082 vs. 16561 EUR; p = 0.359), total LoS (22 days vs. 28 median days; p = 0.390) or mortality (20.7% vs. 20.7%; p = 1.000) | Single centre and missing data on potentially important factors | 0.56 |
| [115] | Carbapenem resistant *A. baumannii* | Carbapenem susceptible *A. baumannii* | 30-day mortality, ICU and hospital LoS, hospital cost (including days, fees, procedures, tests and drugs - unit cost applied per patient) | Columbia | Adult patients | ICUs in 3 hospitals | Cox proportional hazards regression for mortality and generalized linear models for LoS and cost | 104 cases, 165 total | Resistance was not significantly associated with 30-day mortality [HR =1.45 (95% CI; 0.74–2.87), p=0.28] or LoS [adjusted mean days 19.3 vs 16.2, p=0.548] but was associated with increased costs [11359 vs 7049 USD, p<0.001] for resistant vs susceptible respectively | Small sample size and potentially other influencing factors | 0.56 |
| [116] | Drug resistant HIV | Drug susceptible HIV | Mortality | China | >= 18 years old | 4 counties/provinces | Kaplan-Meier and Cox proportional hazards regression for mortality. Significance tests for LoS | Drug-resistant mutations were found in 235 out of 365 total cases | Drug resistance in the first year of HIV drug resistance was significantly associated with a higher probability of death than drug resistance beyond the first year (p= 0.002). Cox proportional hazards modelling: death was significantly associated with drug resistance [HR= 1.9 (95% CI; 1.2–3.1) p = 0.046]. | Generalisability issue and missing data (initial baseline data for some patients) | 0.78 |
| [117] | General resistance in bacterial infections | General susceptible in bacterial infections | Mortality and repeat surgery and limb amputation | Brazil | All patient who had undergone peripheral artery revascularization surgery | 1 hospital (Not specified) | Kaplan-Meier survival analysis and Cox multiple regression (Wald method) | 46 resistant cases and 24 non-resistant | Resistance was not significantly associated with mortality (no factor found to be significant with p<0.05). With the Kaplan-Meier analysis comparing resistant to non-resistant strains also showed resistance was not significantly associated with survival (p=0.652) | Small sample size and potential bias with case definition/exclusion criteria | 0.78 |
| [118] | XDR *P. aeruginosa* | Colonization and/or infection by a non-gram-negative pathogen or no infection | Infection-related mortality & LoS | Singapore | >16 year olds with hematologic malignancies | 1 hospital | Matching & Significance tests | 26 cases and 53 controls (controls did not have a gram negative bacilli infection) | The infection-attributed mortality of the cases during the study period was 53% (14 cases), being statistically significant (p <0 .01). Median duration of hospitalization was 41 days (range, 19-83 days) in cases after infection isolation and 28 days (range, 5-97 days) in controls (p<0.01) | Single centre, retrospective, small sample size, potential colonisation in controls | 0.44 |
| [119] | Carbapenem resistant *P. aeruginosa* | Carbapenem susceptible *P. aeruginosa* | All-cause in-hospital mortality | Taiwan | All | National surveillance system - 26 hospitals | Matching and logistic regression | 82 resistant cases and 82 controls | Resistance was not associated with mortality (not included in regression analysis) | Retrospective, small sample size to investigate further factors and potential generalisability issues internationally | 0.67 |
| [120] | MRSA (septic) arthritis | MSSA (septic) arthritis | In-hospital all-cause mortality | Taiwan | All | 2 hospitals | Significance test | 38 MRSA cases, 55 MSSA cases | Resistance was not significantly associated with mortality (p=0.67) | Small sample size, retrospective nature, issue of generalisability, not attributable mortality investigated | 0.44 |
| [121] | Vancomycin-resistant enterococci colonisations and infections | Non-exposure patients in hospital | Attributable (healthcare) cost and attributable LoS (after onset) | Canada | >=18 years old | 1 hospital | Generalized linear modelling approach for associations with both outcomes (patient costs were provided by the finance records) | 217 VRE cases and 1075 non-VRE controls | VRE was a significant independent variable for total cost (p<0.01), estimating the attributable cost of VRE to be $17,949 (Canadian dollars)[95% CI; 13,949-21,464] and also estimating an average of 13.8 additional days [95% CI; 10.0 - 16.9]. There was no statistically significant difference in the attributable cost of VRE between patients with infections and patients with colonisations (data not shown). | Potential confounding factors not included, the endogenous variable bias arising from the relationship between VRE and LoS, a relatively small sample size and not including societal costs | 0.56 |
| [122] † | KPC-2-producing *K. pneumoniae*  infection (post-colonization) | Non KPC-2-producing patients | Mortality | Germany | All | 1 hospital | Significance tests | 9 KPC-2-producing cases, 18 controls | Hospital mortality was higher in the cases [78 % (KPC-2-KP cases) vs. 11 % (controls); p=0.001], LoS was also significantly higher (60 vs. 32 days; p=0.035). Relative risk of mortality for cases was 7.0 (95 % CI; 1.8–27.1). | Missing potentially important factors for analysis, potential confounding with excess mortality | 0.44 |
| [123] | Artemisinin resistant malaria | No resistance | Excess mortality, excess cost of treatment and productivity loss (GDP per capita). | International (Malaria-endemic countries) | All | surveillance, literature | **Modelling Study:** Decision Tree Model | N/A | Annual excess of 116,000 deaths in the widespread resistance scenario, with excess treatment costs of US$32 million per year and excess productivity losses resulting estimated at US$385 million for each year in which failing artemisinin-based combination therapy is used as first-line treatment. Costs in 2013 USD | Limited scope in terms of costs included and assumptions about transmission/treatment failure (including static nature) | 0.44 |
| [124] | Healthcare-acquired infections with MBL- producing *P. aeruginosa* | Healthcare-acquired infections with non-"MBL" *P. aeruginosa* | Death, death related to infection, LoS to death and LoS until discharge | Brazil | All | 1 hospital | Significance tests | 58 MBL- *P. aeruginosa* cases and 26 non-MBL- *P. aeruginosa* cases | MBL- *P. aeruginosa* cases had a faster progression to death (p=0.04), there was no significant association with LoS until discharge (p=0.32), death related to infection (p=0.24) or death (p=0.24) | Retrospective nature and sample size for controls | 0.44 |
| [125] | Healthcare associated MRSA | Non "MRSA" patients | Excess LoS and hospital cost | Switzerland | Adult patients | 1 hospital | Multistate modelling and Cox proportional hazards for excess LoS. Applying unit cost to difference in LoS and also comparison of observed cost | 167 MRSA-infected, 115 MRSA-colonised but uninfected, and 25,766 MRSA-free | Excess LOS attributable to MRSA infection was 11.5 [95% confidence interval (CI): 7.9-15] or 15.3 days according to multistate modelling and matched analysis, respectively. The likelihood of discharge after MRSA infection was significantly less [HR = 0.69 (95% CI: 0.59-0.81)]. MRSA infection resulted in an additional cost of about 800 Swiss francs per day. | Costing method limitations (DRG system has inherent limitations and costing of LoS was for whole episode) | 0.67 |
| [126] | ESBL-producing *E. coli* and *Klebsiella* species Urinary Tract Infections | ESBL-negative *E. coli* and *Klebsiella* species Urinary Tract Infections | Median LoS, median cost, hospital monetary loss and infection-related mortality | USA | >= 18 years old | 1 hospital | Significance tests | 55 cases (ESBL-*E. coli* or *Klebsiella* infections) and 55 controls (non ESBL-*E. coli* or *Klebsiella* infections) | No significant differences were found in infection-related mortality rates (p=0.37), median LoS was significantly longer (6 days (IQR; 4–8) vs 4 days (IQR; 3–6) p=0.02), as was total hospital cost 10,741 USD (IQR; 6846–15,819) vs 7,083 USD (IQR; 5667–11,652) p=0.02. Median differences in cost and reimbursement between ESBL-producers vs non–ESBL-producers were 3658 USD (p=0.02) and 469 USD (p=0.56), median loss per patient with ESBL-*E. coli* or *Klebsiella* infection was 3189 USD (2011-2012 USD) | Only one time period studied and potentially missing out important factors for outcomes such as LoS | 0.56 |
| [127] | MRSA bacteremia | MSSA bacteremia | 30-day and 90-day mortality (methicillin resistant) | USA | Adult | 1 hospital | Logistic regression | 15 MRSA cases, 21 MSSA cases | No significant difference in either outcome; for 30-day mortality OR=0.62(95% CI; 0.15-2.61) p=0.52, for 90-day mortality OR=0.51 (95% CI; 0.12 - 2.14), p=0.36 | Single centre, potential testing issues and retrospective nature | 0.56 |
| [128] | *S. aureus* Heterogeneous intermediate resistance to vancomycin (hVISA) infectious endocarditis (IE) | MRSA infectious endocarditis infectious endocarditis (IE) | Mortality | Israel | All | 1 hospital | Kalplan-Meier analysis to compare survival and significance tests | 14 hVISA IE cases, 32 MRSA IE controls | Both groups were associated with high mortality but there was not a significant difference between groups (p=0.6) | Small sample size, retrospective nature and potential impact of other factors | 0.44 |
| [129] † | Resistant BSI | Susceptible BSI | 48 hour fatality rate and 30-day case fatality rate | Spain | All patients with a solid tumour | 1 specialist centre | Stepwise Logistic regression | 489 BSI episodes investigated - 61 MDR organisms, mostly ESBL-producing Enterobacteriaceae (n = 20), AMP-C-producing Enterobacteriaceae (n = 13), MRSA (n = 7), MDR *P. aeruginosa* (n = 5), *S. maltophilia* (n = 1), and *A. baumanniii* (n = 1) | Resistance was not an independent risk factor for mortality - MDR Gram positive and Gram negative bacilli had a p value = 0.22 in the univariate analysis and was not included in the multivariate analysis | Single centre and case definition | 0.78 |
| [130] | 3GC resistant *E. coli* and *K. pneumoniae*  nosocomial bloodstream infections | 3GC susceptible *E. coli* and *K. pneumoniae*  bloodstream infections | 7-day and 28-day mortality | Finland | All patients | 4-17 hospitals over time | Significance tests | 182 3GC resistant and 2,035 susceptible E. coli bloodstream infections, 25 3GC resistant and 636 susceptible Klebsiella pnuemoniae bloodstream infections | Resistance was not significantly associated with increased mortality rates (p>0.05) | Unstable sample over the study period and missing data on potentially important factors such as antibiotic usage and previous healthcare contact. | 0.44 |
| [131] | Resistant community acquired respiratory tract, urinary tract or skin and soft tissue infections | Susceptible community acquired respiratory tract, urinary tract or skin and soft tissue infections | 30-day mortality | England | >64 years old | 1 hospital and local health board records | Logistic regression | 161 total cases, 12 resistant Gram negative infections, 24 infections with any resistant organism | Resistance was not significantly associated with mortality [OR = 2.10 (95% CI; 0.26 - 16.77), p = 0.486] | Retrospective nature, data limitations, small sample size and potential important factors missing | 0.67 |
| [132] | Hospital-acquired bacteremia (MDR or partial resistance multiple species) | Susceptible hospital-acquired bacteremia | 7-day all-cause mortality | England | All inpatients | 1 hospital trust | Multivariate logistic regression analysis (variables chosen by univariate analysis significance) | 559 cases in total | Partial resistance and MDR were not found to be significantly associated with 7-day mortality [OR = 1.11 (95% CI; 0.67 to 1.81), p=0.690 and OR= 1.54 (95% CI; 0.94 to 2.54), p=0.090 respectively] - but neither were included in the multivariate analysis and therefore no ORs presented | Case definition limitations (not including healthcare-associated), missing information (on treatment), outcome assumptions (discharged meant survival for 7 days post-discharge) and all-cause mortality was used | 0.44 |
| [133] | MDR *P. aeruginosa*  healthcare associated pneumonia | Non-MDR *P. aeruginosa*  healthcare associated pneumonia | In-hospital mortality | International (Europe & US) | >=18 years old | 12 hospitals (5 countries) | Cox proportional hazards model | 226 MDR cases, 514 non-MDR cases | MDR was significantly associated with mortality [HR = 1.39 (95% CI; 1.05, 1.83) p=0.021] | Retrospective nature, case definition (heterogeneity in laboratories) and use of hospital mortality rather than other outcome measures | 0.56 |
| [134] | MDR *P. aeruginosa*  Nosocomial Pneumonia | Non-"MDR" *P. aeruginosa*  Nosocomial Pneumonia | In-hospital mortality | International (United States, France, Germany, Italy and Spain) | Adult patients | International network | Logistic regression | 50 cases were MDR, 162 total | MDR was significantly associated with mortality [OR=5.50 (95% CI; 3.56–8.51), p <0.001] | Selection bias, case definition heterogeneity and missing information on potentially important factors | 0.56 |
| [135] | Fluconazole resistant Gram-negative rod bacteraemia | Fluconazole susceptible Gram-negative rod bacteraemia | 30-day all-cause cumulative mortality (& risk of death) | USA | >= 18 year old hematopoietic cell transplant patients | 1 cancer centre | Kaplan-Meier survival analysis and Cox proportional hazards | 88 resistant cases, 167 susceptible cases, 25 no resistance data | Resistance was significantly associated with mortality (p=0.018) and increased risk of death (HR 2.11 (1.06 - 4.23)) | Retrospective and observational nature, missing data on potential factors, generalisability | 0.56 |
| [136] | MDR tuberculosis | Non-MDR tuberculosis | Treatment costs (infrastructure, wages, services consumed), hospital LoS, number of specialised visits and tests | Latvia | >=18 years old | National control programme and administrative records | Multivariate regression analysis | 92 MDR-TB, 54 non MDR-TB | MDR-TB cases were estimated to have an additional 18,729 USD in inpatient treatment costs, 2,199 USD additional prescription medication costs and 21,520 USD total costs (p<0.001) [2002 USD] compared to non-MDR TB patients. It was estimated that MDR TB patients had significantly increased hospital LoS (475.3 vs. 110.2, p-value<0.001) compared to non MDR-TB patients | Relatively small sample size, potential ascertainment bias amongst control, cross-sectional data and health-system cost only | 0.56 |
| [5] | MDR tuberculosis | Not applicable | Patient perceived socio-economic cost | Mexico | Adult patients recently completed treatment | Local programme | **Qualitative study:** Structured interviews and qualitative analysis | 20 study participants included | MDR-TB impacted individual socio-economic burden [majority of participants reported reductions in salary after MDR-TB illness] | Generalisability issue and recall bias | N/A |
| [137] | Healthcare associated MRSA infections | No MRSA infection inpatient | Length of stay and costs (Fixed and variable inpatient costs, assumed 2010 USD) | USA | Veteran Affairs patients | National network | Generalized linear modelling - "conventional costing method", "post-HAI method") & “Matching method” (whereby timing of MRSA HAIs was accounted for by propensity score matching separately for each day of the hospital stay) | For the conventional costing method; 382,812 non-MRSA cases and 3,982 MRSA cases. For the post-HAI method; 121,428 non-MRSA cases, 92 MRSA cases. For the matching method; 10,120 non-MRSA cases and 2,872 MRSA cases | Excess length of stay estimates were 17.64 (95% CI; 17.58-17.71, p< 0.0001) days, 11.43 (95% CI; 10.44- 12.43, p < 0.0001) days and 13.97 (95% CI; 10.49-17.44,p < 0.0001) days for the conventional, post-HAI and matching methods respectively. Excess total inpatient cost estimates were $31,570 (95% CI; $30,074 - $33,067, p < 0.0001), $24,015 (95% CI; $10,882 - $37,149,p < 0.0001) and $26,855 (95% CI; $22,583 - $31,126, p < 0.0001) respectively (assumed 2010 USD) | Selection bias and generalisability | 1.00 |
| [138] | Healthcare associated MRSA infections | Non-“ Healthcare associated MRSA infections” patients | Post-discharge healthcare costs and Utilization (readmission, antibiotic usage and hospital days), 365-day follow up | USA | Veteran Affairs patients | National network | Matching (propensity score) & multivariable regression (generalized linear model for cost, logistic and binomial for utilisation) | 3,592 MRSA cases and 3,592 matched controls | Positive MRSA culture was significantly associated increased inpatient costs of $12,167 (p <0 .0001). Positive MRSA culture was significantly associated with a 13.8% increase in number of prescriptions (p <0.0001), a 39.6% increased odds of having a readmission (Pp<0 .0001), and 20.4% more inpatient days (p <0 .0001) (costs in 2013 USD) | Generalisability and focus on colonisation not infection | 0.89 |
| [139] | Healthcare-associated MRSA colonisation and infections | Non-"Healthcare-associated MRSA colonisation and infections" | Post-discharge mortality (post-discharge 365 day mortality) | USA | Veteran Affairs patients | National network | Cox proportional hazards & propensity score matching | "In the ""full cohort""; No positive MRSA culture - 366,144, Positive MRSA culture - 3,599. In the ""matched"" cohort; No positive MRSA culture- 3,592 and positive MRSA | MRSA infection had a significant impact on post-discharge mortality (for the full cohort HR = 1.489, 95% CI; 1.261 - 1.758, p =0<.0001, for the matched cohort HR = 1.464, 95% CI; 1.212 - 1.769, p <.0001). | Using secondary data, potentially not accounting for time-dependent confounding, a specific patient population was utilised and potential issues with case definitions | 1.00 |
| [140] | Carbapenem resistant nonbacteremic *K. pneumoniae* infections (pneumonia and UTI) | non-carbapenem-resistant, non-ESBL-producing nonbacteremic *K. pneumoniae* infections (pneumonia and UTI) | In-hospital mortality, 90-day mortality, 30-day readmission | USA | Adult patients | 1 hospital | Variable significance tests | 48 resistant cases and 48 controls | Resistance had a non-significant and positive impact on in-hospital mortality (14% vs 10%; p =0.76), 90-day mortality (24% vs 14%; p=0.31) and 30-day readmissions (32% vs 19%; p = 0.21). | Small sample size | 0.56 |
| [141] | Methicillin-resistant *staphylococci* infections | Methicillin-susceptible *staphylococci* infections | In-hospital mortality | Japan | Patients who have undergone hepatectomy and then paracentesis for postoperative ascites | 1 hospital | Cox proportional hazards model | 13 methicillin-resistant staphylococci positive cases and 61 negative cases | Methicillin-resistant staphylococci was found to be a significant risk factor for hospital mortality in the multivariate analysis; HR=5.076 (95% CI; 1.07-24.39) p=0.041. | Only patients who had undergone paracentesis were included and false-negative results of bacterial peritonitis | 0.67 |
| [142] | ESBL-producing Enterobacter bacteraemia | ESBL-negative Enterobacter bacteraemia | Mortality, related mortality and LoS | Brazil | All | 1 hospital | Significance tests - not clear | 41 ESBL-positive cases, 164 ESBL-negative cases | There was no significant difference in mortality between groups. There was a longer LoS for ESBL-positive cases but unclear if significant from study reporting | Not explicitly outlined | 0.44 |
| [143] † | Carbapenem resistance *K. pneumoniae* infection and colonization | Non-"Carbapenem resistance *K. pneumoniae*" patients | Mean LoS, hospital mortality | Italy | Elderly | 1 hospital | Variable significance tests | 133 resistant cases, 400 controls | Resistance was related to increased length of hospital stay (35 days (+/- 24) vs 18 days (+/- 12), p<0.001) but not significantly related to mortality rate (21% vs 15% , p=0.08) | Seasonality not taken into account | 0.56 |
| [144] | Capromycin resistant XDR tuberculosis | Capromycin susceptible XDR tuberculosis | Mortality and culture conversion | South Africa | All | 1 hospital | Kaplan-Meier survival analysis | 47 capreomycin resistant cases of 52 that were genotyped | Resistance was not significantly associated with mortality (p=0.0573) or culture conversion (p=0.35) | Generalisability and length of follow up | 0.56 |
| [145] | MRSA Infective Endocarditis | Non-"MRSA" Infective Endocarditis | Mortality | Japan | Post valve surgery patients | 1 hospital | Logistic regression | 9 MRSA cases, 109 total cases | MRSA was not associated with mortality in the multivariate analysis [OR=1.08 (95% CI; 0.04-28.88) p=0.962] | Potential important factors not included | 0.67 |
| [146] | Third generation cephalosporin resistant Enterobacteriaceae | Third generation cephalosporin susceptible Enterobacteriaceae | In-hospital mortality, LoS | Brazil | >=18 years old | 1 hospital | Multivariate analysis for mortality, Cox proportional hazards model for LoS, significance tests on excess days | 62 resistant patients, 124 susceptible controls | Resistance was not significantly associated with mortality in the multivariate analysis ("non-significant" presented) but was significantly associated with LoS (p<0.001). Hospital LoS was13 days for cases and 5 for controls (p<0.001) | Single centre and small sample size | 0.56 |
| [147] | MRSA bacteraemia | MSSA bacteraemia | visual outcome (visual acuity) | Taiwan | All | 1 hospital | Linear regression | 26 MRSA cases, 59 keratitis cases | MRSA was not significantly associated with visual outcome | Retrospective nature, small sample size and potential issues with case and outcome definition | 0.56 |
| [148] | MRSA keratitis | MSSA keratitis | All-cause mortality and survival, long term survival (>200 days) | Australia | All | 1 hospital | Kaplan-Meier analysis and Cox proportional hazards regression | 76 MRSA cases, 363 MSSA cases | MRSA bacteraemia had a median survival of 398 days and were more likely to die than those with MSSA bacteraemia (log-rank P < 0.0001;HR=2.87, 95% CI 1.87 to 4.39). However the Cox regression revealed MRSA was not a significant independent factor for long-term survival (HR 1.37, 95% CI; 0.95-1.97, p=0.10). | No non-*Staphylococcus* bacteraemia, all-cause mortality was the outcome (rather than attributable) and potential missed outcomes (deaths), potentially missing factors that could affect the outcome, retrospective nature | 0.56 |
| [149] | MDR bloodstream infections | Non-"MDR" bloodstream infections | 7-day mortality | Italy | ICU patients staying in ICU longer than 48 hours | 1 ICU | Significance tests | MDR Enterobacteriaceae cases were 50, with respective non-MDR cases at 28. MDR *K. pneumoniae*  carbapenemase cases were 31, with respective non-MDR cases at 17 and MDR *P. aeruginosa*  cases were 21, with respective non-MDR cases at 20. | MDR significantly impacted Enterobacteriaceae (RR=2.17 (95% CI; 1.16-4.05) p<0.01), *K. pneumoniae*  carbapenemase producing strains (RR=2.63 (95 % CI; 1.23-5.63) p<0.01) and *P. aeruginosa*  (RR=1.90 (95% CI; 0.89-4.09), p=0.0080). It did not significantly affect other tested bacteria. | Single centre, secondary data and all-cause mortality | 0.33 |
| [150] | Methicillin-resistant complicated skin and soft tissue infections | Methicillin-susceptible complicated skin and soft tissue infections | LoS | Europe | >= 18 years old | 129 sites across 10 countries | Descriptive analysis and multivariate analysis. | 102 MRSA cases and 274 MSSA cases | Patients with confirmed diagnosis of MRSA infection had longer mean hospital stays than patients with MSSA infection (27.7 days [SD= 23.9; median = 19.5] vs 18.4 days [SD = 19.9; median = 13.0]). MRSA was not investigated as a factor in the multivariate analysis. | Missing potentially important factors for analysis | 0.56 |
| [151] | ESBL-producing gram negative bacteriuria/urinary tract infection | Non-"ESBL-producing" gram negative bacteriuria/urinary tract infection | LoS, IC admission, antimicrobial treatment and all-cause in-hospital mortality | Australia | >= 18 years old | 1 hospital | Significance tests for all outcomes apart from ICU admission which used logistic regression | 100 cases, 100 controls | ESBL-GNB was not significantly associated with all-cause mortality (p=0.4) and duration of treatment (p=0.1), but was associated with LoS after sampling (6 vs 2 days, p=0.002). ESBL-GNB was significantly associated with ICU admission, OR=4.6 (95% CI; 2.0 - 10.8), p<0.001 | Retrospective nature, limited matching, small sample size, potential bias in inclusion criteria | 0.44 |
| [152] | VAP caused by XDR *A. baumannii*  (XDRAB) | VAP caused by non-"XDR" *A. baumannii* | In-hospital mortality and LoS (ICU and hospital mean) | Turkey | Adult | 1 hospital ICU | Significance tests | 34 XDR cases, 134 *A. baumannii*  VAP cases | XDR was not significantly associated with mortality (p=0.858) but was significantly associated with longer ICU (30.4 vs 21.7 mean days, p=0.005) and hospital LoS (39.0 vs 13.2 mean days 0.001) | No clear stated limitations | 0.56 |
| [153] | KPC-producing *K. pneumoniae* | Non-"KPC-producing" *K. pneumoniae*  colonization and infection | ICU mortality | Greece | All | 1 hospital ICU | Logistic regression | 164 KPC–positive cases and 62 KPC-negative controls | KPC+ *K. pneumoniae* infection (not colonisation) during the ICU stay was found to be a significant factor for ICU mortality, OR= 2.8 (95% CI; 1.1–6.9), p=0.026 | Single centre, potential testing issues and ICU design issues | 0.78 |
| [154] | High vancomycin MIC MRSA BSI | Low vancomycin MIC MRSA BSI | 30-day mortality | South Korea | Adult patients | 1 hospital | Cox proportional hazards model | 31 MRSA cases with high vancomycin MIC, 63 MRSA cases with low vancomycin MIC | Vancomycin MIC was not significantly associated with mortality (p=0.69 in significance tests and not included in multivariate analysis) | Generalisability and case definition issues | 0.67 |
| [155] | MDR, carbapenem resistant *A. baumannii*  or non-*baumannii* ACB complex bacteremia | Susceptible *A. baumannii*  or non-*baumannii* ACB complex bacteremia | Infection-related mortality and 30-day mortality | South Korea | >=16 years old | 1 hospital | Multivariate logistic regression analysis (variables chosen by univariate analysis significance) | 98 MDR cases, 63 carbapenem resistant cases and 180 total cases | MDR and carbapenem resistance were found to be significant predictors of mortality in the univariate analysis (p=0.002 and p=0.001, respectively) but not in the multivariate logistic regression (detailed results not given) | Retrospective nature, missing potential confounding factors and single centre. | 0.56 |
| [156] | Carbapenem resistant *A. baumannii*  Bacteremia | Susceptible *A. baumannii*  or non-*baumannii* ACB complex bacteremia | 30-day mortality | South Korea | All | 1 hospital | Logistic regression | 52 carbapenem resistant cases out of 123 total | Carbapenem resistance was independently associated with 30-day mortality [OR= 4.01, 95% CI (1.51 - 10.68), p= 0.005] | Retrospective nature | 0.78 |
| [157] | High vancomycin MIC & hVISA community associated MRSA bacteremia | Non-"High vancomycin MIC & hVISA" community associated MRSA bacteremia | Crude mortality | South Korea | >=16 years old | 1 hospital that also provides primary care | Logistic regression for mortality, significance tests for LoS | 342 MRSA bacteraemia cases | Vancomycin MIC and hVISA were not associated with mortality (p>0.4 for all, not included in the multivariate analysis) | Single centre, international generalisability issues, potential other factors missing | 0.78 |
| [158] | Gram-negative bacilli susceptible to <=1 antibiotic | Gram-negative bacilli susceptible to vs >=2 | 7-, 15- and 30-day mortality | USA | All | 16 ICUs in association with 1 hospital | Significance tests | 103 cases susceptible to <=1 antibiotic, 195 controls (vs susceptible to >=2) | Resistance (case status) was not significantly associated with mortality for any time point (7-,15- and 30-day mortality; p= 0.87, 0.20 & 0.14 respectively) | Generalisability issue, case definition bias and not attributable mortality | 0.67 |
| [159] | High carbapenem MIC Enterobacteriaceae infections | Low carbapenem MIC Enterobacteriaceae infections | 30-day mortality, LoS and ICU LoS | USA | >= 18 years old | 1 hospital | Matching, significance tests and a Kaplan-Meier analysis for survival | 18 patients with MIC of 1 mg/litre and 18 patients with MIC of 2–8 mg/litre | Cases with carbapenem MICs of 2, 4, and 8 mg/litre had significantly higher 30-day mortality than those in the group with carbapenem MICs of 1 mg/litre (p = 0.04). The high-MIC group was associated with decreased overall survival by Kaplan-Meier log rank test (p=0.01). Mean total hospital LoS was longer but not significantly (57.6 days vs 34.4 days, p= 0.06), mean ICU LoS was significantly longer (56.6 days vs 21.7 days, p=0.01) in the group with MICs of 2 to 8 mg/litre than in the group with MICs of 1 mg/litre. | Potentially important cofounding factors not included, potential testing issue, small sample size and retrospective nature | 0.56 |
| [160] | ESBL-positive *K. pneumoniae*  bacteraemia | ESBL-positive *K. pneumoniae*  bacteraemia | 30-day mortality and direct medical cost (antimicrobial usage, hospitalization and laboratory and diagnostic tests during the infection-related length of hospital stay) | Hong Kong | Adult patients | 1 hospital | Logistic regression | 32 out of the 208 patients were infected with ESBL-producing *K. pneumoniae* | ESBL production was not included in the multivariate models for cost or 30-day mortality as was not found to be significant in significance tests or univariate analysis (p>0.05) | Retrospective nature, sample size and not using excess mortality | 0.56 |
| [161] | ESBL-producing microorganisms in sepsis | ESBL-negative microorganisms in sepsis | Mean duration of hospitalisation | Iran | Neonate patients | 1 hospital | Significance tests | 68 ESBL producing isolates, 103 neonates total | Mean hospital duration was longer in patients infected with beta-lactamase producers (30.2±20.5 vs. 22.8±16.6 days p=0.05) and ESBL producing strains (29.13±20.39 vs. 19±9.84 p=0.05) | None clearly stated | 0.44 |
| [162] | MDR *P. aeruginosa* | Non-"MDR" *P. aeruginosa* | Mortality | China | All | 5 hospitals | Significance tests | 188 resistant cases, 348 total | The median length of stay of the resistant patients (39 days) was greater than that of non-resistant patients (24 days) (p <0 .01). The statistical difference of mortality was significant between the two groups (26.1% vs 12.5% respectively, p <0 .01). | Selection bias and incomplete follow up | 0.44 |
| [163] | Carbapenem-resistant *K. pneumoniae*  Infections | Carbapenem-susceptible *K. pneumoniae*  Infections and and no Klebsiella Pnuemoniae Infection | Post-transplant survival | USA | Liver transplant recipients | 1 hospital | Cox regression | CRKP Infection (n = 20), No CRKP Infection (n =284) [susceptible infection (n = 36) and no *K. pneumoniae*  infection (n= 248)] | Resistance was did not significantly impact mortality compared to susceptible strains, comparing the confidence intervals that results from modelling each of these groups against no *Klebsiella* infection [CRKP HR = 6.92 (95% CI, 3.24- 14.79) and susceptible infection HR = 3.84 (95% CI, 1.86-7.94)] | Small sample size & generalisability | 0.78 |
| [164] | MDR & XDR tuberculosis | Non-"MDR or XDR" tuberculosis | Direct and indirect medical costs and non-medical costs | South Africa | All | National control programme | **Modelling Study:** Evidence synthesis and stepwise calculations (e.g. outcomes from published literature * unit cost ) | 741 XDR-TB cases, 7,386 MDR-TB cases and 336,332 sensitive TB cases | Total cost for XDR-TB was estimated to be 26,392.01 USD, MDR TB to be 6771.92 USD and 256.61 USD for susceptible TB. The costs changed minimally in the sensitivity analysis. All costs in 2011 USD. | Generalisability issue, potential validity issue, potential additional cost factors | 0.54 |
| [165] | MRSA BSI | MSSA BSI | Sepsis-related mortality and LoS | Brazil | All | 1 hospital | Significance test | 61 cases, 169 controls | Resistance was significantly associated with increased mortality (p=0.0134) | Small sample size | 0.56 |
| [166] | MDR tuberculosis | Rifampicin susceptible tuberculosis | Mortality | Europe and Argentina | All | Not stated | Cox regression | 55 MDR cases out of 175 total, 90 rifampicin susceptible | MDR TB (vs. RH-susceptible TB) was significantly associated with mortality [HR=2.28 (95% CI 1.00, 5.20), p =0.050] | Potential case selection bias due to data completeness for certain cases | 0.67 |
| [167] | Carbapenem resistant *K. pneumoniae*  bacteriuria | Carbapenem susceptible *K. pneumoniae*  bacteriuria | Overall mortality | USA | Adult post-kidney transplant patents | 2 medical centres | Kaplan-Meier survival curves and Cox proportional hazards | 20 CRKP cases and 80 CSKP cases | Resistance was significantly associated with mortality [HR=3.0 (95% CI; 1.0-9.0) p=0.04] | Generalisability issue (in low endemicity areas) and small sample size | 0.56 |
| [168] | Peritonitis (VRE, ESBL & MBL producers in multiple species) | Susceptible peritonitis | Mortality | India | All patients on peritoneal dialysis (for end-stage renal disease) | 1 hospital | Significance tests | 211 total | Resistance was significantly associated with mortality [VRE - p=0.001, ESBL - p= 0.23, MBL - p=0.038] | No clear stated limitations | 0.56 |
| [169] | *P. aeruginosa* , Enterobacteriaceae extended-spectrum β-lactamase–positive, and MRSA (PES) community associated-pneumonia | Non-"PES" community associated-pneumonia | 30-day mortality and LoS | Spain | All | 1 hospital | Logistic regression for mortality, significance tests for LoS | 94 PES patients, 1503 non-PES patients | PES patients had a significantly longer hospital stay (p<0.001) and PES was an independent factor for 30-day mortality [odds ratio = 2.51 (95% CI 1.20–5.25) P = 0.015] | Single centre, small sample size, missing data on potentially important factors | 0.67 |
| [170] | Candida isolates non-susceptible to fluconazole. | Candida isolates susceptible to fluconazole. | 7-day mortality | Spain | Cancer patients >= 16 years old | Regional network | Logistic regression | 53 non-susceptible cases and 193 susceptible cases | Resistance was not found to be significant in the univariate and analysis [OR=0.75 (95% CI; 0.26–2.16) p=0.598] and was not included in the multivariate analysis. | Observational nature, generalisability & sample size | 0.67 |
| [171] | Carbapenem-resistant *K. pneumoniae*  bacteriuria | None | Development of secondary healthcare associated infection | USA | Adult | 1 medical centre | Univariate and multivariate logistic regression | 105 cases total | Resistance not associated with secondary healthcare associated infections (which were notably absent) | Retrospective nature and generalisability issue | 0.78 |
| [172] | MRSA Ventilator Associated Pneumonia/Hospital Associated Pneumonia | MSSA Ventilator Associated Pneumonia/Hospital Associated Pneumonia | Time to clinical stability | International (Europe) | All | 27 ICUs | Significance tests | 30 MRSA cases and 15 MSSA controls | MRSA had a significant impact on time to clinical stability (p<0.05) for all the following indicators (mean days (SD)): for example fever - 8.4(3.8) for MRSA vs 2.8(0.8) for MSSA and leukocytes - 8.02(5.5) for MRSA vs 6.93(4.2) for MSSA | Heterogeneity of local protocols and treatments | 0.56 |
| [173] | Drug-resistant Streptococcus pneumonia | Susceptible Streptococcus pneumonia | Annual and incremental cost burden. Included: 1) direct costs (including medical care), 2) costs from adverse outcomes, 3) work-loss costs, and 4) cost from lost wages | USA | All | Literature, administrative data sources and expert panel | **Modelling Study:** Decision Tree Model | N/A | Resistance was estimated to account for 4% (2012 $91 million) of annual pneumococcal pneumonia direct medical costs and 5% ($233 million) of total costs (including work and productivity loss). Most of the incremental medical cost ($82 of $91 million) was estimated to be due to hospitalisations resulting from erythromycin resistance. Increased resistance to erythromycin was associated with the greatest projected cost | Potentially 'old' data and model assumptions (epidemiological/structural and biological) e.g. model assumed an increase in resistant strains would not change prescribing practices | 0.53 |
| [174] | Resistant Staphylococcus aureus bacteraemia | Susceptible Staphylococcus aureus bacteraemia | 30-day all-cause mortality | Germany | All | 2 hospitals | Multivariate logistic regression analysis (variables chosen by univariate analysis significance) | 31 MRSA cases, 317 total, 71% beta lactamase postive and 6% macrolide, lincosamide, streptogramin resistant | Resistance was significantly associated with 30-day mortality; MRSA; OR= 4.80 (95%CI; 1.43 - 16.06, p=0.01), Beta-lactamase; OR=3.12 (1.17-8.30, p=0.02), Macrolide, lincosamide, streptogramin resistance; OR=4.64 (1.32 - 16.35, p=0.02) | Generalisability issue and relatively small sample size | 0.56 |
| [175] | Ciprofloxacin resistant and Amoxicilline-clavulanate resistant ESBL+ *E. coli* bacteraemia | Ciprofloxacin susceptible and Amoxicilline-clavulanate susceptible ESBL+ *E. coli* bacteraemia | 30-day mortality | Spain | All | 13 hospitals | Multivariate logistic regression analysis (variables chosen by univariate analysis significance) | 129 ciprofloxacin resistant cases, 23 amoxicillin-clavulanate resistant cases and 191 ESBL+ *E. coli* cases total | Ciprofloxacin resistance was not significantly associated with mortality in the univariate analysis [RR=1.4 (95 CI; 0.78-2.50) p=0.2) and not included in the multivariate analysis. Amoxicillin-clavulanate resistance was significantly associated with mortality in the multivariate analysis [OR 2.15, 95% CI; 0.98 - 4.71, p=0.05]. | Relatively small sample size, generalisability issue (multicentre but only applicable to those with similar epidemiology) | 0.78 |
| [176] | Vancomycin-resistant MRSA pneumonia | Vancomycin-susceptible MRSA pneumonia | 60-day mortality | International | Adult patients | Parent study was an RCT - multicentre and international | Logistic regression | 381 MRSA cases | Vancomycin heteroresistance was not a risk factor for mortality | Did not take into account factors such as LoS, potential bias due to case diagnosis | 0.89 |
| [177] | MRSA healthcare-associated pneumonia | Non-"MRSA" healthcare-associated pneumonia | In-hospital mortality | Japan | All | 1 hospital | Logistic regression | 7 resistant cases, 6 sensitive cases | MRSA was independently associated with in-hospital mortality in the multivariate analysis [OR=15.56 (95% CI; 1.86–218.22) p=0.020] | Single centre | 0.67 |
| [178] | XDR *P. aeruginosa* infections. | Non-"XDR" *P. aeruginosa* infections. | All-cause and infection-related in-hospital mortality | Greece | All cancer patients | 1 hospital | Univariate and multivariate logistic regression | 22 XDR cases, 89 in total | The multivariate analysis showed that infection due to XDR isolates [OR=5.11, (95 % CI 1.15–22.62)] was independently associated with mortality. | Retrospective nature, small sample, single centre and potential issues with case definition | 0.78 |
| [179] | Ventilator Associated Pneumonia (multiple species -resistant organisms) | Susceptible Ventilator Associated Pneumonia | Mortality | India | >=18 years old | 4 ICUs from 1 hospital | Matching and significance test | 52 VAP cases total | MDR and PDR *Pseudomonas* was significantly associated with mortality (p=0.045) | Single centre and missing data on potential factors | 0.56 |
| [180] | Third-generation cephalosporin resistant Gram-negative BSI | Non-"Third-generation cephalosporin resistant Gram-negative" BSI | Attributable mortality | Ethiopia | Adult Patients | 1 hospital | Logistic regression | 38 patients with a positive blood culture, 21 third-generation cephalosporin resistant | Third-generation cephalosporin resistance was significantly associated with mortality OR=23.28 (95% CI; 3.3–164.4) | Not clearly stated | 0.67 |
| [181] | MRSA bacteraemia following pneumonia | Non-"MRSA" bacteraemia following pneumonia | In-hospital all-cause mortality, hospital LoS | USA | >18 years old | 1 hospital | Logistic regression for in-hospital mortality, general linear model for LoS | 93 bacteraemia cases, 672 non-bacteraemia controls | MRSA bacteraemia was not an independent factor for mortality, but had a trend towards this [OR = 1.56; 0.93 - 2.61]. MRSA bacteraemia was associated with additional LoS of 10.3 days (95 % CI 6.7 to 13.9 days, p < 0.001). | Retrospective nature, single site, small sample size and lack of data for potentially important variables | 0.56 |
| [182] | MDR tuberculosis | Non-"MDR" tuberculosis | TB-related mortality | Israel | All patients | National Registry | Multivariate logistic regression | 196 MDR cases, 4555 TB patients in total | MDR-TB was significantly associated with TB-related death [OR= 2.83 (95%CI; 1.70–4.72), p<0.001] | Potential bias with case selection (single investigator), incomplete data (on patient information such as comorbidities) and data quality issues (death certificates) | 0.67 |
| [183] | Carbapenem resistant *K. pneumoniae* | Carbapenem susceptible *K. pneumoniae* | Mortality | USA | All kidney transplant patients | 1 hospital | Kaplan-Meier survival analysis and Cox proportional hazards model | 13 resistant cases and 39 susceptible controls | Resistance was significantly associated with death in all the models [HR= 8.8 (95% CI; 2.2–35.8), p=0.002 in the unadjusted Cox model) | Retrospective nature, small sample size and generalisability issue | 0.56 |
| [184] | Fluconazole resistant Cryptococcus neoformans | Fluconazole susceptible Cryptococcus neoformans | Clinical variables | Uganda | All | 1 hospital | Significance tests & Generalized Linear Model | 61 susceptible, 33 dose-susceptible and 4 resistant isolates | Resistance did not impact any of the clinical variables of interest (p>0.05 for all) | Not clearly stated | 0.44 |
| [185] | Febrile urinary tract infections - healthcare associated and community associated (ESBL and AmpC- producing, multiple species) | Susceptible febrile urinary tract infections | All-cause in-hospital mortality | Spain | Adult males | 1 hospital | Logistic regression | 29 ESBL- or AmpC- producing cases, 479 total | ESBL- and AmpC- production was not significantly associated with mortality (in univariate and was not selected for multivariate) where p=0.16 | Single centre, retrospective, potential for selection bias and generalisability issue | 0.67 |
| [186] | MDR Hospital acquired infections | Non-"MDR" Hospital acquired infections | Mortality | Brazil | >=18 years old | 1 hospital | Significance tests | 889 total, 393 colonised with MDR and 497 not colonised, 318 infected with MDR and 571 not infected with MDR | The relative risk for mortality when comparing colonised with not colonised with MDR was (RR=) 1.37(1.16 - 1.62 95% CI) with a p<0.001 and the relative risk for mortality with comparing infected with not infected was (RR)=1.28(1.09 - 1.51 95% CI) with a p=0.004. | None stated | 0.56 |
| [187] | ESBL-positive and/or beta-lactamases (IBL)-positive, and ciprofloxacin resistant UTI | Susceptible UTI | Median duration of hospitalisation and median antibiotic costs | Turkey | All | 1 hospital | Significance tests | 45 ESBL- and/or IBL- positive cases, 43 ESBL- and/or IBL-negative cases, 48 ciprofloxacin resistant cases, 40 ciprofloxacin susceptible cases. | ESBL- and/or IBL- positive cases had significantly higher average length of hospitalisation compared to ESBL- and/or IBL-negative cases [9(3-24) days versus 5(2-14) days, p=0.001]. Ciprofloxacin resistant cases had significantly higher average length of hospitalisation compared to ciprofloxacin susceptible cases [9(2-24) days versus 4(2-17) days, p=0.001].ESBL- and/or IBL- positive cases had significantly higher antibiotic costs compared to ESBL- and/or IBL-negative cases[110.6(5.5-505.2) 2014 USD versus 19.8(6.50-384.2), p=0.001]. Ciprofloxacin resistant cases had significantly higher antibiotic costs compared to ciprofloxacin suscpetible cases [135.1(5.5 - 505.2)] compared to susceptible cases [19.8(6.5-234.6), p=0.001] (2014 USD) | Retrospective and small sample size | 0.44 |
| [188] | ESBL-producing Enterobacteriaceae BSIs | ESBL-negative Enterobacteriaceae BSIs & non-exposure inpatients | In-hospital mortality, LoS and payer cost (all attributable) | Switzerland | All | 1 hospital | Multistate models and Cox proportional hazards models for mortality and LoS, calculation for cost (LoS * unit cost) | 30 ESBL-positive cases and 96 ESBL-negative cases and all-inpatients for a year as controls (42,476 patients) | Excess LoS attributable to ESBL-positive was 9.4 days (95% CI; 0.4-18.4) and ESBL-negative was 2.6 (0.7-5.9), making ESBL-positivity associated with 6.8 excess days, hazard ratios for discharge were HR=0.62 (95% CI; 0.43-0.89) and HR= 0.90 (95% CI; 0.74-1.10) for ESBL-positive and negative respectively. Cost per acute care episode estimated at 48,674 CHF for ESBL-positive cases, 48,131 CHF for ESBL-negative cases and 13,532 CHF for controls (2009 CHF). ESBL-positivity led to an excess cost of 9,473 per Enterobacteriaceae bloodstream infection. The independent effect of ESBL-positivity was not significant on in-hospital mortality [OR=2.8 (95% CI; 0.7–11.5), p=0.15]. | Single centre, generalisability issue, cost calculation only included LoS and an accounting cost (not taking into account fixed versus variable costs) | 0.67 |
| [189] | High Vancomycin MIC in invasive Staphylococcus aureus infections | Low Vancomycin MIC in invasive Staphylococcus aureus infections | Lethality | Brazil | All | 1 hospital | Significance tests | 24 patients with MIC <2mg/L and 15 MIC 2>=2 mg/L | Resistance was not significantly associated with mortality in all significance and microbiology tests (p>0.2) | No clear stated limitations | 0.44 |
| [190] | High vancomycin MIC in MRSA pneumonia | Low vancomycin MIC in MRSA pneumonia | 30-day all-cause mortality | Canada | >= 18 year olds | 11 hospitals | Logistic regression | 161 cases total, 60 cases with vancomycin MIC ≥ 1.5 µg/mL | Vancomycin MIC ≥ 1.5 µg/mL was associated with mortality [OR 2.5 (95% CI 1.0-6.3), p=0.051] | Potential case definition bias and potentially missing important confounders | 0.78 |
| [191] | MRSA BSI (heterogeneous vancomycin-intermediate resistance (hVISA) and β -lactam antibiotic-induced vancomycin resistance (BIVR) phenotypes impact) | MRSA BSI (vancomycin susceptible and β -lactam antibiotic-induced vancomycin susceptible) | 30-day all-cause mortality | Japan | All | 1 hospital | Logistic regression | 162 MRSA isolates; 34 hVISA and 39 BIVR | hVISA and BIVR positivity was associated with mortality [OR= 7.66 (95% CI 1.28 – 45.6) p = 0.025] | Retrospective nature and small sample size | 0.67 |
| [192] | VRE infection | No VRE infection | Mortality (time from transplant to all-cause death) | USA | Adult allogeneic hematopoietic cell transplantation patient | 1 clinic | Cox proportional hazards regression | 76 VRE-bacteraemia cases, 800 patients | VRE- bacteraemia was significantly associated with worse mortality [HR= 4.28, (95% CI 3.23–5.66) P<0.001] in multivariable analysis. | Single centre | 0.67 |
| [193] | Resistance in multiple species - 3 bacteria & 3 other microbes | No resistance (different scenarios presented) | Monetary cost and proportion of GDP loss | Global | All | Surveillance, literature, expert opinion | **Modelling Study:** Economic impact was modelled through a dynamic equilibrium model - Computable general equilibrium models | N/A | On average over a forty year horizon, the world GDP loss was between USD 53 billion to 3 trillion per year - main regions affected by AMR were Eurasia, the High region and, to a lesser extent, due to its comparatively lower income, Sub-Saharan Africa. However, Sub-Saharan Africa would be the most negatively affected by AMR relative to its GDP. The costs in year 10 ranged from USD 10 billion to 625 billion. But by year 40, the range increased from 188 billion to 9.8 trillion. | Only a certain number of diseases and only looking at impact on GDP and did not include 'secondary' health care cost. Did not account for the fact that increased healthcare spending can contribute the economy and offset some of the decreases, as increased hospital time requires work and consumption. Did not look at cost of action. Uncertainty around the parameter inputs due to lack of information. | 0.55 |
| [194] | MDR Ventilator associated pneumonia | Non-"MDR" Ventilator associated pneumonia | 28-day survival, hospital mortality, LoS (hospital & ICU) | USA | >= 18 years old | 1 hospital | 28-day survival modelled by Kaplan-Meier analysis, Cox proportional hazards model used for hospital mortality and significance tests for other outcomes | 49 MDR cases, 107 VAP cases total | Resistance was significantly associated with 28-day survival (p=0.006), hospital LoS (37 vs 31 median days, p=0.07), ICU LoS (31 vs 27, p=0.08), however this differed when cases split further into different organism types. In non-fermenting Gram-negative rods [HR=1.37(95% CI: 0.62-3.06)], there was no association between MDR and in-hospital mortality, but, in all other organisms, MDR was significantly associated with increased mortality [HR= 6.15 (95% CI: 1.80-21.05) p = 0.004]. | Retrospective nature, single centre, case definition issues and not enough power to explore further | 0.67 |
| [195] | General resistance to standard prophylactic antibiotics | No resistance | Infection-related mortality | USA | Post -Caesarean section, Transrectal prostate biopsy, Spinal surgery, Surgical abortion, Hysterectomy, Pacemaker implantation, Total hip replacement, Appendectomy, Colorectal surgery, Hip fracture and Cancer chemotherapy (leukaemia, lymphoma, myeloma). | Literature | **Modelling Study:** Evidence synthesis and stepwise calculations | N/A | Between 38.7% and 50.9% of pathogens causing surgical site infections and 26.8% of pathogens causing infections after chemotherapy were resistant to standard prophylactic antibiotics in the USA. A 30% reduction in the efficacy of antibiotic prophylaxis for these procedures would result in 6,300 infection-related deaths (2,100 for a10% reduction - 15 000 for a 70% reduction). 13 120 infections (42%) per year after prostate biopsy were attributable to fluoroquinolone resistance. | Case definition issues, potential missing information on important factors (such as high-risk vs low-risk patients or infection control practices) and assumptions of resistance being similar across organisms. | 0.49 |
| [196] | MRSA bacteraemia | MSSA bacteraemia | Total cost of inpatient care main outcome, mortality and LoS | Canada | >=18 years old | 4 hospitals | Costs from Ontario Case Costing Initiative - included laboratory tests, medicines, nursing care, administration and IT costs, comparison of MRSA vs MSSA by linear regression models with propensity score weighting. Significance tests for LoS | 58 MRSA cases 3375 MSSA cases | Resistance was not significantly associated with increased costs [MRSA had 1.3 times higher direct costs than MSSA (95% CI; 0.91-1.85), p=0.148], mortality [p=0.2863] or median hospital LoS [22.50 vs 14.00, p=0.0951]. It was significantly associated with increased ICU LoS [12 vs 6 median days, p=0.0313]. | Retrospective, only those with a laboratory report within 72 hours of admission, some costs like physician costs or home care costs not included | 0.67 |
| [197] | MDR *P. aeruginosa*  bacteremia | Non-"MDR" *P. aeruginosa*  bacteremia | Mortality and ICU LoS | Germany | All burn patients | 1 specialist ICU | Mortality - multivariate logistic regression, LoS - Cox proportional hazards model | 45 cases, 42 controls | In the logistic regression MDR was not significantly independently associated with mortality [OR =1.076 (95% CI 0.356–3.254), p = 0.897]. From the Kaplan–Meier analysis time in the burn ICU after bacteraemia was not significantly different in both groups (median stay MDR bacteraemia 25 days vs susceptible PAB 19 days; p =0.945 by the log-rank test). In the Cox proportional hazards regression MDR was not significantly independently associated with burn ICU LoS (HR = 0.994 (95% CI 0.513–1.925) p = 0.985] | Small sample size and lack of data (e.g. antibiotic treatment) | 0.67 |
| [198] | MRSA bacteraemia | MSSA bacteraemia | Mortality and burn ICU LoS | Germany | Burn patients | 1 burn ICU | Backward selection logistic regression for mortality and univariate Kaplan-Meier analysis (with the log-rank test) and multivariate Cox proportional hazards model for ICU LoS | 33 MRSA cases and 41 MSSA controls | MRSA did not significantly increase the risk for mortality compared with MSSA [OR=1.55, (95% CI; 0.56–4.28), p= 0.40). With the Kaplan–Meier analysis, burn ICU LoS after bacteraemia was not significantly different (median MRSA 28.5 days vs. MSSA 21.0 days; p= 0.32). In the multivariate Cox proportional-hazards regression methicillin resistance was not significantly independently associated with longer burn ICU LoS after bacteraemia [HR=1.22, (95% CI 0.65–2.27); p = 0.535]. | Time dependent bias for LoS and missing information (on antibiotic treatment) | 0.67 |
| [199] | MDR *P. aeruginosa* pneumonia | Non-"MDR" *P. aeruginosa* pneumonia | ICU mortality | Italy | >=18 years old | 1 hospital ICU | Logistic regression and Kaplan-Meier | 42 MDR cases, 110 PA pneumonia cases in total | MDR was not significantly associated with mortality from the multivariate analysis (MDR result not presented) | Single centre, retrospective and potential other factors that may affect mortality missing | 0.67 |
| [200] | Colistin resistant *K. pneumoniae*  carbapenemase infections | Colistin susceptible *K. pneumoniae*  carbapenemase infections | 14-day mortality | Italy | >= 18 years old | 5 hospitals | Logistic regression | Colistin resistance in 62 out of 132 cases | Colistin resistance was found to be an independent risk factor for 14-day mortality [OR=2.18 (95% CI, 1.37–3.46) p =0.001] | Retrospective nature, possible misclassification and observational nature | 0.78 |
| [201] | High fluconazole MIC Candida albicans candidaemia | Low fluconazole MIC Candida albicans candidaemia | Infection-related 30-day mortality | Australia | All | National network | Logistic regression | 5 cases had an MIC =< 0.125, 217 cases in general with varying MIC results | There was a significant association between rising MICs and infection-related mortality [OR=1.4 for every MIC stratum (95% CI 1.06–1.71) p=0.015] when the fluconazole MIC was treated as a continuous variable | Retrospective and small numbers in the higher MIC levels | 0.67 |
| [202] | Carbapenem resistant Klebsiella pnuemoniae infection | Carbapenem susceptible Klebsiella pnuemoniae infection | Mortality and ICU LoS | Greece | All | 1 hospital ICU | Logistic regression for mortality, significance tests for LoS | 80 CRKP, 24 CSKP cases | Resistance was not significantly associated with mortality (p=0.19) and ICU stay (31 vs 26.2 mean days, p=0.21) | No clear stated limitations | 0.67 |
| [203] | MDR pneumonia (multiple species) | Non-"MDR" pneumonia (multiple species) | In-ICU and 28-day mortality | Singapore | All | 1 hospital ICU | Cox proportional hazards regression analysis (variables chosen by univariate unadjusted hazard ratios) | 119 multidrug resistant cases, 230 non-multidrug resistant cases | Multidrug resistance was not significantly associated with in-ICU mortality [unadjusted HR=1.43 (0.86-2.88, p=0.17)] so not included in the adjusted model, but was significantly associated with 28-day mortality [adjusted HR=1.65 (1.01 - 2.68, p=0.04)]. | Quantitative sputum cultures not performed | 0.67 |
| [204] | Nosocomial Resistant Gram-negative Bacilli infection | Nosocomial susceptible Gram-negative Bacilli infection | Total hospital cost, cost per day, LoS | Singapore | Adult patients (>21 years) | Medical and Surgical ICU in 1 hospital | Propensity score matching and significance tests | 76 resistant cases, 65 susceptible infected controls, 1549 patients in total | Resistance was significantly associated with increased total hospitalisation cost [p=0.0001 compared to no infection, $2795.9 vs $2009.6 p=0.0004 compared to susceptible infection]. Resistance was associated (not significantly) with increased total hospital stay and hospital stay after admission to the ICU when compared to no infection [p=0.01 for both] but not when compared to susceptible infections [p=0.39 and p=0.41 respectively] | Single centre, only measured direct costs | 0.67 |
| [205] | MDR *P. aeruginosa*  keratitis | Non-"MDR" *P. aeruginosa*  keratitis | Clinical outcome (e.g. resolution rates) and duration of therapy | India | All | Unclear | Significance test | 23 MDR cases and 67 non-MDR controls | Resistance was not associated with duration of therapy or resolution rates. However, there was a significant difference in some clinical outcomes. | Retrospective study and potential case definition bias | 0.44 |
| [206] | Carbapenem-resistant *A. baumannii* | Carbapenem-susceptible *A. baumannii* | Mortality and ICU LoS | Lithuania | >=18 years old | 1 hospital | Logistic regression | 40 CRAB cases and 59 carbapenem-sensitive cases | Resistance was not significantly associated with mortality (p=0.461). Resistance was associated with a longer ICU stay after infection [36.12 vs 19.33 mean days, p=0.005] | No clear stated limitations | 0.56 |
| [207] | Heteroresistant vancomycin-intermediate MRSA bacteraemia | Non-"Heteroresistant vancomycin-intermediate " MRSA bacteraemia | 30-day survival | Taiwan | All | 1 hospital | Cox regression | 16 hVISA cases, 284 MRSA bacteraemia cases total | High vancomycin MIC and VSSA was associated with 30-day mortality [OR = 2.349 (95% CI; 1.078–5.118)], but high vancomycin MIC and hVISA was not [OR = 2.163 (95% CI; 0.686–6.821)] | Small sample size, potential other factors impacting outcomes, generalisability issues, potential case definition issues | 0.56 |
| [208] | Isoniazid-monoresistant tuberculosis | Isoniazid susceptible tuberculosis | Successful treatment (including cure) and unsuccessful treatment (including death) | Taiwan | All | 1 hospital | Logistic regression | 90 high resistant concentration cases, 44 low concentration cases | Resistance was not significantly associated with mortality [OR = 1.03 (95% CI; 0.29–3.61), p= 0.969] | Retrospective nature, small sample and missing info (genetic analysis not performed) | 0.67 |
| [209] | Caspofungin resistant and MDR Candidaemia | Susceptible Candidaemia | 30-day and 14-day all-cause mortality | USA | >= 18 years with acute leukaemia | 1 cancer centre | Cox regression | 14 caspofungin resistant & 13 MDR isolates out of 69 isolates where susceptibility available | Caspofungin resistance was significantly associated with mortality [14-day mortality - HR = 3.02 (95% CI; 1.28–7.09), p=0.011, 30-day mortality - HR= 2.96 (95% CI; 1.38–6.37), p=0.05], MDR was significantly associated with mortality [14-day mortality HR=3.02 (1.27–7.14), p=0.012, 30-day mortality HR= 2.86 (95% CI; 1.31–6.21), p=0.008] | Retrospective nature, generalisability issue, crude rather than attributable mortality as the outcome | 0.56 |
| [210] | Carbapenem resistant bloodstream infections | Non-"Carbapenem resistant" bloodstream infections | Infection-related mortality | China | Febrile Neutropenia Patients Undergoing Hematopoietic Stem Cell Transplantation | 1 transplant unit | Logistic regression | 12 carbapenem resistant cases and 85 total | Resistance was significantly associated with mortality [RR=4.4 (95% CI; 1.14–17.28), p=0.041] | Retrospective nature, small sample size & generalisability | 0.78 |
| [211] | Ceftriaxone-resistant *Streptococcus pneumoniae* pneumonia | Ceftriaxone-susceptible *Streptococcus pneumoniae* pneumonia | Clinical cure, (infection-related) LoS, in-hospital mortality, 30-day readmissions | USA | 18 – 89 years old | 1 medical centre | Variable significance tests | 10 resistant, 20 susceptible cases | Resistance was not significantly related to any of the outcomes. Results for resistant vs susceptible were: median LoS 17 vs 15, p=0.46, infection-related LoS 9 vs 8 p=0.74, in-hospital mortality p=1.00, and 30-day readmission for pneumonia p=1.00. | Retrospective and single centre | 0.44 |
| [212] | Metallo-beta-lactamase-producing *P. aeruginosa* | Metallo-beta-lactamase-negative *P. aeruginosa* | In-hospital mortality and LoS | Germany | Adult patients | 3 hospitals | Cox regression | 18 MBL-PA and 95 non-MBL-PA isolates | Neither MBL-PA nor MDR-PA remained associated with mortality in the multivariate analysis [MBL-PA HR= 0.98 (95% CI; 0.45-2.1), p=0.97, MDR-PA HR= 1.37(0.68-2.72), p=0.37]. No significant influence of MBL-PA or MDR-PA on LOS in the fully adjusted models. | Small sample | 0.67 |
| [213] | MDR tuberculosis | Non-"MDR" tuberculosis | TB treatment outcome (e.g. adverse) and TB cost (direct medical care and non-medical, lost income, total expense and 'catastrophic' costs) | Peru | >15 years old | Survey from National control programme | Regression (univariate and multivariate) | 93 MDR TB cases, 876 TB cases, 487 controls | MDR was associated with adverse treatment outcome [OR=8.37 (95% CI; 4.67–15.0), p<0.001] and independently associated with incurring catastrophic costs [OR=1.61 (95% CI = 0.98–2.64), p<0.06] | Potential other cost variables and different definitions of cost variables, bias caused by follow-up length, and generalisability issues. | 0.78 |
| [214] | Community onset UTIs caused by multiple species (levofloxacin- or cefazolin-nonsusceptible isolatesor uropathogens with ESBL producers) | Susceptible community onset UTIs | LoS | Taiwan | >= 18 years old | 1 hospital | Significance tests | 136 total cases, 14 ESBL-producing Enterobacteriaceae isolates, of which all but one were resistant to levofloxacin. Out of 122 non-ESBL producing isolates there were 35, 31 and 16 non-susceptible to levofloxacin, cefazolin, and ceftriaxone, respectively. | LoS was longer in to levofloxacin non-susceptible (16.1 vs. 7.5 days; p<0.01), cefazolin-non-susceptible (15.4 vs.8.4 days; p<0.01), or ESBL-producing (16.7 vs. 9.6 days; p<0.01) cases, compared to antimicrobial-susceptible comparators. | Limited scope in terms of outcomes and missing potentially important factors | 0.44 |
| [215] | MRSA healthcare-associated infections | MSSA and non-Staphylococcus aureus healthcare-associated infections | Fatality and hospital LoS | China | All | 1 hospital | Cox proportional hazards for the LoS and logistic regression for fatality | 57 MRSA cases, 116 MSSA cases, 102 non-*Staphylococcus* cases | MRSA infections were associated with increased risk of fatality (MRSA versus MSSA) [OR=2.7, 95% CI 1.0–7.0], (MRSA versus *S. aureus* negative) [OR 31.6, 95% CI 4.4-inf]. MRSA was significantly associated with increased LoS compared to MSSA [OR 1.5 (95% CI; 1.0-2.2), p=0.046] and to non-*Staphylococcus* cases [OR 1.8(95% CI; 1.2-2.7), p=0.002]. | Retrospective nature, potential medical coding errors and a relatively small sample size | 0.56 |
| [216] | MRSA Mediastinitis | Non-"MRSA" Mediastinitis | Mortality | Turkey | Adult mediastinitis patients post-open heart surgery | 1 hospital | Logistic regression | 47 MRSA cases, 117 total | MRSA was associated with mortality [OR = 12.11, 95 % CI; 3.15– 46.4, p= 0.000] | Retrospective nature and missing data (vancomycin MICs) | 0.56 |
| [217] | MDR *P. aeruginosa*  infections | Susceptible *P. aeruginosa*  infections | Mortality and length of stay | China | Paediatric burns patients | 1 hospital | Logistic regression & Matching (the methods are not clear regarding results then presented) | Unclear case and control defined sample sizes | OR=4.7, p= 0.03 for resistance impact on mortality and OR=2.0, p=0.002 for resistance impact on length of stay. (Hazard ratios were also presented for length of stay, however it is not clear in the methods section how these were derived) | Not clearly stated | 0.33 |
| [218] | MRSA infections | MSSA infections | Death and duration of hospitalization (categorised) | Pakistan | Up to 12 years | 1 hospital | Significance tests | 30 MRSA cases, 30 MSSA cases | Duration of hospitalization was significantly longer in the MRSA group as compared to MSSA group (p= 0.02 comparing number of patients staying in the 7 - 21 day group), with significantly more susceptible patients staying less than 7 days compared to the resistant group (p=0.04). Twelve patients from the MRSA group remained from less than 7 days duration while this number was 23 for MSSA group; p-value =0.04). Mortality was significantly higher in the MRSA group compared to the MSSA group (p-value =0.001). | Not clearly stated | 0.33 |

Abbreviations: 3GC; third-generation cephalosporin, AU$; australian dollars, BSI; bloodstream infection CHF; swiss francs, *A. baumanniii; A. baumanniii ,* ART; antiretroviral therapy, BSI; bloodstream infection, CR; carbapenem-resistant, CRAB; carbapenem-resistant *A. baumanniii* , CRKP; Carbapenem-resistant *K. pneumoniae* , *E. coli*; *E. coli*, ESBL; Extended Spectrum Beta-lactamase, E*. arogenes; Enterobacter aeogenes*, ESC; extended-spectrum cephalosporin, EUR; Euros, GDP; Gross Domestic Product, GNR; Gram-negative rod, HAI; healthcare-associated infection, HR; hazard ratio, hVISA; heterogeneous vancomycin-intermediate *Staphylococcus aureus*, IBL; Beta-lactamase, ICU; intensive care unit, INR; Indian rupee, IRR; incidence rate ratio, *K. pneumoniae; K. pneumoniae* , KPC; *K. pneumoniae*  Carbapenemase, LoS; length of stay, MBL; metallo-beta-lactamase, MDR; multidrug resistance, ME; multiplicative effect, MIC; minimum inhibitory concentration, N/A; not applicable or not available, MRSA; MRSA, MSSA; MSSA, OR; odds ratio, p; p-value, PDR; pan drug resistant, *P. aeruginosa; P. aeruginosa* , RR; relative risk, TB; tuberculosis, SLI; injectable second-line drug, *S. maltophilia; Stenotrophomonas maltophilia*, UTI; urinary tract infection, VAP; ventilator associated pneumonia, VSSA; vancomycin susceptible *Staphylococcus aureus*, VRE; Vancomycin-resistant *Enterococcus,* VSE; Vancomycin-suscpetible *Enterococcus*, XDR; extensively drug resistant

**References**

1. Wells G., Shea B, O’Connell D, Peterson J, Welch V, Losos M, et al. The Newcastle-Ottawa Scale (NOS) for assessing the quality of nonrandomised studies in meta-analyses [Internet]. University of Ottawa. 2014 [cited 2016 Feb 1]. Available from: http://www.ohri.ca/programs/clinical_epidemiology/oxford.asp

2. Hartling L, Hamm M, Milne A, Vandermeer B, Santaguida PL, Ansari M, et al. Validity and Inter-Rater Reliability Testing of Quality Assessment Instruments. Validity and Inter-Rater Reliability Testing of Quality Assessment Instruments. 2012.

3. Philips Z, Bojke L, Sculpher M, Claxton K, Golder S. Good Practice Guidelines for Decision-Analytic Modelling in Health Technology Assessment. PharmacoEconomics. 2006;24:355–71.

4. Higgins JPT, Green S. Cochrane Handbook for Systematic Reviews of Interventions Version 5.1.0 [updated March 2011]. The Cochrane Collaboration. 2011. p. Table 7.7.a: Formulae for combining groups.

5. Morris MD, Quezada L, Bhat P, Moser K, Smith J, Perez H, et al. Social, Economic, and Psychological Impacts of MDR-TB Treatment in Tijuana, Mexico: A Patient’s Perspective. Int J Tuberc Lung Dis. 2013;17:954–60.

6. Abernethy JK, Johnson AP, Guy R, Hinton N, Sheridan EA, Hope RJJ. Thirty day all-cause mortality in patients with Escherichia coli bacteraemia in England. Clinical Microbiology and Infection. Elsevier; 2015;21:251.e1-251.e8.

7. Al Mohajer M, Musher DM, Minard CG, Darouiche RO. Clinical significance of Staphylococcus aureus bacteriuria at a tertiary care hospital. Scandinavian Journal of Infectious Diseases. 2013;45:688–95.

8. Allen KB, Fowler VGJ, Gammie JS, Hazel J., Onorato M., DiNuble MJ, et al. Comparative Effectiveness of Induction Therapy for Human Immunodeficiency Virus-Associated Cryptococcal Meningitis: A Network Meta-Analysis. Open Forum Infectious Diseases. 2015;2:1–8.

9. Aminzadeh Z, Yadegarynia D, Fatemi A, Tahmasebian Dehkordi E, Azad Armaki S. Vancomycin minimum inhibitory concentration for methicillin-resistant Staphylococcus aureus infections; is there difference in mortality between patients? Jundishapur Journal of Microbiology. 2014;7:2012–4.

10. Amit S, Mishali H, Kotlovsky T, Schwaber MJ, Carmeli Y. Bloodstream infections among carriers of carbapenem-resistant Klebsiella pneumoniae: Etiology, incidence and predictors. Clinical Microbiology and Infection. Elsevier; 2015;21:30–4.

11. The AMR Review. Antimicrobial Resistance : Tackling a crisis for the health and wealth of nations. 2014.

12. Andria N, Henig O, Kotler O, Domchenko A, Oren I, Zuckerman T, et al. Mortality burden related to infection with carbapenem-resistant Gram-negative bacteria among haematological cancer patients: A retrospective cohort study. Journal of Antimicrobial Chemotherapy. 2015;70:3146–53.

13. Anuwatnonthakate A, Whitehead SJ, Varma JK, Silachamroon U, Kasetjaroen Y, Moolphate S, et al. Effect of mycobacterial drug resistance patterns on patients’ survival: a cohort study in Thailand. Glob J Health Sci. 2013;5:60–72.

14. Apostolopoulou E, Raftopoulos V, Filntisis G, Kithreotis P, Stefanidis E, Galanis P, et al. Surveillance of device-associated infection rates and mortality in 3 greek intensive care units. American Journal of Critical Care. 2013;22.

15. Apostolopoulou E, Raftopoulos V, Zarkadas P, Toska A, Veldekis D, Tsilidis K. Risk factors and attributable mortality of carbapenem-resistant acinetobacter baumannii infections. Health Science Journal. 2014;8:126–36.

16. Babu KV, Visweswaraiah D, Kumar A. The influence of Imipenem resistant metallo-beta-lactamase positive and negative Pseudomonas aeruginosa nosocomial infections on mortality and morbidity. Journal of Natural Science, Biology and Medicine. 2014;5:345.

17. Balcan B, Olgun S, Torlak F, Sagmen SB, Eryuksel E, Karakurt S. Determination of Factors Affecting Mortality of Patients with Sepsis in a Tertiary Intensive Care Unit. Turkish Thoracic Journal/Türk Toraks Dergisi. 2015;16:128–32.

18. Barnett AG, Page K, Campbell M, Martin E, Rashleigh-Rolls R, Halton K, et al. The increased risks of death and extra lengths of hospital and ICU stay from hospital-acquired bloodstream infections: a case-control study. BMJ Open. 2013;3:e003587.

19. Bergin SP, Thaden JT, Ericson JE, Cross H, Messina J, Clark RH, et al. Neonatal Escherichia coli Bloodstream Infections: Clinical Outcomes and Impact of Initial Antibiotic Therapy. The Pediatric infectious disease journal. 2015;34:933–6.

20. Biehle LR, Cottreau JM, Thompson DJ, Filipek RL, O’Donnell JN, Lasco TM, et al. Outcomes and risk factors for mortality among patients treated with carbapenems for klebsiella spp. bacteremia. PLoS ONE. 2015;10:6–13.

21. Bodro M, Sanclemente G, Lipperheide I, Allali M, Marco F, Bosch J, et al. Impact of antibiotic resistance on the development of recurrent and relapsing symptomatic urinary tract infection in kidney recipients. American Journal of Transplantation. 2015;15:1021–7.

22. Boender TS, Hoenderboom BM, Sigaloff KCE, Hamers RL, Wellington M, Shamu T, et al. Pretreatment HIV drug resistance increases regimen switches in sub-saharan Africa. Clinical Infectious Diseases. 2015;61:1749–58.

23. Boncagni F, Francolini R, Nataloni S, Skrami E, Gesuita R, Donati A, et al. Epidemiology and clinical outcome of healthcare-associated Infections: A 4-year experience of an Italian ICU. Minerva Anestesiologica. 2015;81:765–75.

24. Branch-Elliman W, Lee GM, Golen TH, Gold HS, Baldini LM, Wright SB. Health and Economic Burden of Post-Partum Staphylococcus aureus Breast Abscess. PLoS ONE. 2013;8:1–7.

25. Capone A, Giannella M, Fortini D, Giordano A, Meledandri M, Ballardini M, et al. High rate of colistin resistance among patients with carbapenem-resistant Klebsiella pneumoniae infection accounts for an excess of mortality. Clinical Microbiology and Infection. European Society of Clinical Infectious Diseases; 2013;19:E23–30.

26. Casapao a. M, Leonard SN, Davis SL, Lodise TP, Patel N, Goff D a., et al. Clinical Outcomes in Patients with Heterogeneous Vancomycin-Intermediate Staphylococcus aureus Bloodstream Infection. Antimicrobial Agents and Chemotherapy. 2013;57:4252–9.

27. Castón JJ, González-Gasca F, Porras L, Illescas S, Romero MD, Gijón J. High vancomycin minimum inhibitory concentration is associated with poor outcome in patients with methicillin-susceptible Staphylococcus aureus bacteremia regardless of treatment. Scandinavian journal of infectious diseases. 2014;46:783–6.

28. Centers for Disease Control and Prevention. Antibiotic resistance threats in the United States, 2013. Current. 2013;114.

29. Chan MCW, Lee N, Lui GCY, Ngai KKL, Wong RYK, Choi KW, et al. Comparisons of oseltamivir-resistant (H275Y) and concurrent oseltamivir-susceptible seasonal influenza A(H1N1) virus infections in hospitalized adults, 2008-2009. Influenza and other Respiratory Viruses. 2013;7:235–9.

30. Chandy SJ, Naik GS, Balaji V, Jeyaseelan V, Thomas K, Lundborg CS. High cost burden and health consequences of antibiotic resistance: The price to pay. Journal of Infection in Developing Countries. 2014;8:1096–102.

31. Chaulk J, Carbonneau M, Qamar H, Keough A, Chang HJ, Ma M, et al. Third-generation cephalosporin-resistant spontaneous bacterial peritonitis: a single-centre experience and summary of existing studies. Can J Gastroenterol Hepatol. 2014;28:83–8.

32. Cheah ALY, Spelman T, Liew D, Peel T, Howden BP, Spelman D, et al. Enterococcal bacteraemia: Factors influencing mortality, length of stay and costs of hospitalization. Clinical Microbiology and Infection. European Society of Clinical Infectious Diseases; 2013;19:E181–9.

33. Chittawatanarat K, Jaipakdee W, Chotirosniramit N, Chandacham K, Jirapongcharoenlap T, K. CC, et al. Microbiology, resistance patterns, and risk factors of mortality in ventilator-associated bacterial pneumonia in a Northern Thai tertiary-care university based general surgical intensive care unit. Infection and drug resistance. 2014;7:203–10.

34. Cho S-Y, Lee D-G, Choi S-M, Kwon J-C, Kim S-H, Choi J-K, et al. Impact of vancomycin resistance on mortality in neutropenic patients with enterococcal bloodstream infection: a retrospective study. BMC infectious diseases. 2013;13:504.

35. Choi HR, Bedair H. Mortality following revision total knee arthroplasty: A matched cohort study of septic versus aseptic revisions. Journal of Arthroplasty. Elsevier Inc.; 2014;29:1216–8.

36. Chopra T, Marchaim D, Awali R a., Krishna a., Johnson P, Tansek R, et al. Epidemiology of Bloodstream Infections Caused by Acinetobacter baumannii and Impact of Drug Resistance to both Carbapenems and Ampicillin-Sulbactam on Clinical Outcomes. Antimicrobial Agents and Chemotherapy. 2013;57:6270–5.

37. Chung-Delgado K, Guillen-Bravo S, Revilla-Montag A, Bernabe-Ortiz A. Mortality among MDR-TB cases: Comparison with drug-susceptible tuberculosis and associated factors. PLoS ONE. 2015;10.

38. Chusri S, Chongsuvivatwong V, Rivera JI, Silpapojakul K, Singkhamanan K, McNeil E, et al. Clinical Outcomes of Hospital-Acquired Infection with Acinetobacter nosocomialis and Acinetobacter pittii. Antimicrobial Agents and Chemotherapy. 2014;58:4172–9.

39. Cilloniz C, Albert RK, Liapikou A, Gabarrus A, Rangel E, Bello S, et al. The Effect of Macrolide Resistance on the Presentation and Outcome of Patients Hospitalized for Streptococcus pneumoniae Pneumonia. American Journal of Respiratory and Critical Care Medicine. 2015;191:1265–72.

40. Cobos-Carrascosa E, Soler-Palacín P, Nieves Larrosa M, Bartolomé R, Martín-Nalda A, Antoinette Frick M, et al. Staphylococcus aureus Bacteremia in Children. The Pediatric Infectious Disease Journal. 2015;34:1329–34.

41. Coccolini F, Sartelli M, Catena F, Montori G, Di Saverio S, Sugrue M, et al. Antibiotic resistance pattern and clinical outcomes in acute cholecystitis: 567 consecutive worldwide patients in a prospective cohort study. International Journal of Surgery. Elsevier Ltd; 2015;21:32–7.

42. Commons RJ, Robinson CH, Gawler D, Davis JS, Price RN. High burden of diabetic foot infections in the top end of Australia: An emerging health crisis (DEFINE study). Diabetes Research and Clinical Practice. 2015;110:147–57.

43. Coombs GW, Nimmo GR, Daly DA, Le TT, Pearson JC, Tan H-L, et al. Australian Staphylococcus aureus Sepsis Outcome Programme annual report, 2013. Communicable diseases intelligence quarterly report. 2014;38:E309-19.

44. Cornejo-Juárez P, Vilar-Compte D, Pérez-Jiménez C, Ñamendys-Silva SA, Sandoval-Hernández S, Volkow-Fernández P. The impact of hospital-acquired infections with multidrug-resistant bacteria in an oncology intensive care unit. International Journal of Infectious Diseases. 2015;31:e31–4.

45. Correa L, Martino MDV, Siqueira I, Pasternak J, Gales AC, Silva CV, et al. A hospital-based matched case–control study to identify clinical outcome and risk factors associated with carbapenem-resistant Klebsiella pneumoniae infection. BMC Infectious Diseases. BMC Infectious Diseases; 2013;13:80.

46. Cortés JA, Reyes P, Gómez CH, Cuervo SI, Rivas P, Casas CA, et al. Clinical and epidemiological characteristics and risk factors for mortality in patients with candidemia in hospitals from Bogotá, Colombia. The Brazilian Journal of Infectious Diseases. 2014;18:631–7.

47. Cox H, Ramma L, Wilkinson L, Azevedo V, Sinanovic E. Cost per patient of treatment for rifampicin-resistant tuberculosis in a community-based programme in Khayelitsha, South Africa. Tropical Medicine and International Health. 2015;20:1337–45.

48. De Jager P, Chirwa T, Naidoo S, Perovic O, Thomas J. Nosocomial outbreak of New Delhi metallo-Beta-lactamase-1-producing Gram-negative bacteria in South Africa: A case-control study. PLoS ONE. 2015;10:1–12.

49. de Oliveira Costa P, Atta EH, da Silva ARA. Predictors of 7- and 30-day mortality in pediatric intensive care unit patients with cancer and hematologic malignancy infected with Gram-negative bacteria. The Brazilian journal of infectious diseases : an official publication of the Brazilian Society of Infectious Diseases. Elsevier Editora Ltda; 2014;18:2–10.

50. Denis B, Lafaurie M, Donay JL, Fontaine JP, Oksenhendler E, Raffoux E, et al. Prevalence, risk factors, and impact on clinical outcome of extended-spectrum beta-lactamase-producing Escherichia coli bacteraemia: A five-year study. International Journal of Infectious Diseases. International Society for Infectious Diseases; 2015;39:1–6.

51. Diel R, Nienhaus A, Lampenius N, Rüsch-Gerdes S, Richter E. Cost of multi drug resistance tuberculosis in Germany. Respiratory Medicine. 2014;108:1677–87.

52. Diel R, Vandeputte J, De Vries G, Stillo J, Wanlin M, Nienhaus A. Costs of tuberculosis disease in the European Union: A systematic analysis and cost calculation. European Respiratory Journal. 2014;43:554–65.

53. Dramowski A, Cotton MF, Rabie H, Whitelaw A. Trends in paediatric bloodstream infections at a South African referral hospital. BMC Pediatrics. 2015;15:33.

54. Ducomble T, Faucheux S, Helbig U, Kaisers UX, K??nig B, Knaust A, et al. Large hospital outbreak of KPC-2-producing Klebsiella pneumoniae: Investigating mortality and the impact of screening for KPC-2 with polymerase chain reaction. Journal of Hospital Infection. 2015;89:179–85.

55. Ericson JE, Popoola VO, Smith PB, Benjamin DK, Fowler VG, Benjamin DK, et al. Burden of Invasive *Staphylococcus aureus* Infections in Hospitalized Infants. JAMA Pediatrics. 2015;169:1105.

56. Ershova J V., Kurbatova E V., Moonan PK, Cegielski JP. Mortality Among Tuberculosis Patients With Acquired Resistance to Second-line Antituberculosis Drugs--United States, 1993-2008. Clinical Infectious Diseases. 2014;59:465–72.

57. Esteve-Palau E, Solande G, S??nchez F, Sorl?? L, Montero M, G??erri R, et al. Clinical and economic impact of urinary tract infections caused by ESBL-producing Escherichia coli requiring hospitalization: A matched cohort study. Journal of Infection. 2015;71:667–74.

58. Farmakiotis D, Kyvernitakis A, Tarrand JJ, Kontoyiannis DP. Early initiation of appropriate treatment is associated with increased survival in cancer patients with Candida glabrata fungaemia: A potential benefit from infectious disease consultation. Clinical Microbiology and Infection. Elsevier; 2015;21:79–86.

59. Fitzpatrick MA, Ozer E, Bolon MK, Hauser AR. Influence of ACB complex genospecies on clinical outcomes in a U.S. hospital with high rates of multidrug resistance. Journal of Infection. 2015;70:144–52.

60. Folgori L, Livadiotti S, Carletti M, Bielicki J, Pontrelli G, Degli Atti MLC, et al. Epidemiology and Clinical Outcomes of Multidrug-Resistant Gram-Negative Bloodstream Infections in a European Tertiary Pediatric Hospital during a 12-Month Period. The Pediatric infectious disease journal. 2014;33:929–32.

61. Ford CD, Lopansri BK, Haydoura S, Snow G, Dascomb KK, Asch J, et al. Frequency, risk factors, and outcomes of vancomycin-resistant Enterococcus colonization and infection in patients with newly diagnosed acute leukemia: different patterns in patients with acute myelogenous and acute lymphoblastic leukemia. Infection control and hospital epidemiology. 2015;36:47–53.

62. Ford CD, Lopansri BK, Gazdik M a, Snow GL, Webb BJ, Konopa KL, et al. The clinical impact of vancomycin-resistant Enterococcus colonization and bloodstream infection in patients undergoing autologous transplantation. Transplant infectious disease : an official journal of the Transplantation Society. 2015;17:688–94.

63. Francis JR, Blyth CC, Colby S, Fagan JM, Waring J. Multidrug-resistant tuberculosis in Western Australia, 1998–2012. The Medical Journal of Australia. 2014;200:328–32.

64. Freire MP, Van Der Heijden IM, do Prado GVB, Cavalcante LS, Boszczowski I, Bonazzi PR, et al. Polymyxin use as a risk factor for colonization or infection with polymyxin-resistant *Acinetobacter baumannii* after liver transplantation. Transplant Infectious Disease. 2014;16:369–78.

65. Fu Q, Ye H, Liu S. Risk factors for extensive drug-resistance and mortality in geriatric inpatients with bacteremia caused by Acinetobacter baumannii. American Journal of Infection Control. Elsevier Inc; 2015;43:857–60.

66. Gandhi NR, Brust JCM, Moodley P, Weissman D, Heo M, Ning Y, et al. Minimal diversity of drug-resistant Mycobacterium tuberculosis strains, South Africa. Emerging Infectious Diseases. 2014;20:426–33.

67. Gulen TA, Guner R, Celikbilek N, Keske S, Tasyaran M. Clinical importance and cost of bacteremia caused by nosocomial multi drug resistant acinetobacter baumannii. International Journal of Infectious Diseases. International Society for Infectious Diseases; 2015;38:32–5.

68. Gürntke S, Kohler C, Steinmetz I, Pfeifer Y, Eller C, Gastmeier P, et al. Molecular epidemiology of extended-spectrum beta-lactamase (ESBL)-positive Klebsiella pneumoniae from bloodstream infections and risk factors for mortality. Journal of Infection and Chemotherapy. Elsevier Ltd; 2014;20:817–9.

69. Ha YE, Kang C-II, Cha MK, Park SY, Wi YM, Chung DR, et al. Epidemiology and clinical outcomes of bloodstream infections caused by extended-spectrum β-lactamase-producing Escherichia coli in patients with cancer. International Journal of Antimicrobial Agents. Elsevier; 2015;42:403–9.

70. Haeusler GM, Mechinaud F, Daley AJ, Starr M, Shann F, Connell TG, et al. Antibiotic-resistant Gram-negative Bacteremia in Pediatric Oncology Patients—Risk Factors and Outcomes. The Pediatric Infectious Disease Journal. 2013;32:723–6.

71. Han SB, Jung SW, Bae EY, Lee JW, Lee D-G, Chung N-G, et al. Extended-spectrum β-lactamase-producing Escherichia coli and Klebsiella pneumoniae bacteremia in febrile neutropenic children. Microbial drug resistance (Larchmont, N.Y.). 2015;21:244–51.

72. Hanberger H, Antonelli M, Holmbom M, Lipman J, Pickkers P, Leone M, et al. Infections, antibiotic treatment and mortality in patients admitted to ICUs in countries considered to have high levels of antibiotic resistance compared to those with low levels. BMC Infectious Diseases. 2014;14:513.

73. Hattemer A, Hauser A, Diaz M, Scheetz M, Shah N, Allen JP, et al. Bacterial and clinical characteristics of health care-and community-acquired bloodstream infections due to pseudomonas aeruginosa. Antimicrobial Agents and Chemotherapy. 2013;57:3969–75.

74. Henig O, Weber G, Hoshen MB, Paul M, German L, Neuberger A, et al. Risk factors for and impact of carbapenem-resistant Acinetobacter baumannii colonization and infection: matched case-control study. European journal of clinical microbiology & infectious diseases : official publication of the European Society of Clinical Microbiology. 2015;34:2063–8.

75. Hernandez C, Cobos-Trigueros N, Feher C, Morata L, De La Calle C, Marco F, et al. Community-onset bacteraemia of unknown origin: clinical characteristics, epidemiology and outcome. European Journal of Clinical Microbiology and Infectious Diseases. 2014;33:1973–80.

76. Hernández C, Feher C, Soriano A, Marco F, Almela M, Cobos-Trigueros N, et al. Clinical characteristics and outcome of elderly patients with community-onset bacteremia. The Journal of infection. 2014;70:135–43.

77. Hill DM, Schroeppel TJ, Magnotti LJ, Clement LP, Sharpe JP, Fischer PE, et al. Methicillin-Resistant *Staphylococcus aureus* in Early Ventilator-Associated Pneumonia: Cause for Concern? Surgical Infections. 2013;14:520–4.

78. Hope R, Blackburn RM, Verlander NQ, Johnson a. P, Kearns a., Hill R, et al. Vancomycin MIC as a predictor of outcome in MRSA bacteraemia in the UK context. Journal of Antimicrobial Chemotherapy. 2013;68:2641–7.

79. Hoxha A, Karki T, Giambi C, Montano C, Sisto A, Bella A, et al. Attributable mortality of carbapenem-resistant Klebsiella pneumoniae infections in a prospective matched cohort study in Italy, 2012-2013. Journal of Hospital Infection. 2016;92:61–6.

80. Hu H-C, Kao K-C, Chiu L-C, Chang C-H, Hung C-Y, Li L-F, et al. Clinical outcomes and molecular typing of heterogenous vancomycin-intermediate Staphylococcus aureus bacteremia in patients in intensive care units. BMC infectious diseases. 2015;15:444.

81. Huang S-R, Liu M-F, Lin C-F, Shi Z-Y. Molecular surveillance and clinical outcomes of carbapenem-resistant Escherichia coli and Klebsiella pneumoniae infections. Journal of microbiology, immunology, and infection. Elsevier Taiwan LLC; 2014;47:1–10.

82. Hübner C, Hübner NO, Hopert K, Maletzki S, Flessa S. Analysis of MRSA-attributed costs of hospitalized patients in Germany. European Journal of Clinical Microbiology and Infectious Diseases. 2014;33:1817–22.

83. Huh K, Kang CI, Kim J, Cho SY, Ha YE, Joo EJ, et al. Risk factors and treatment outcomes of bloodstream infection caused by extended-spectrum cephalosporin-resistant Enterobacter species in adults with cancer. Diagnostic Microbiology and Infectious Disease. Elsevier Inc.; 2014;78:172–7.

84. Hussein K, Raz-Pasteur a., Finkelstein R, Neuberger a., Shachor-Meyouhas Y, Oren I, et al. Impact of carbapenem resistance on the outcome of patients’ hospital-acquired bacteraemia caused by Klebsiella pneumoniae. Journal of Hospital Infection. Elsevier Ltd; 2013;83:307–13.

85. Inchai J, Pothirat C, Bumroongkit C, Limsukon A, Khositsakulchai W, Liwsrisakun C. Prognostic factors associated with mortality of drug-resistant Acinetobacter baumannii ventilator-associated pneumonia. Journal of Intensive Care. 2015;3:9.

86. Iroezindu MO, Chima EI, Isiguzo GC, Mbata GC, Onyedum CC, Onyedibe KI, et al. Sputum bacteriology and antibiotic sensitivity patterns of community-acquired pneumonia in hospitalized adult patients in Nigeria: a 5-year multicentre retrospective study. Scandinavian Journal of Infectious Diseases. 2014;46:875–87.

87. Isea-Peña MC, Sanz-Moreno JC, Esteban J, Fernández-Roblas R, Fernández-Guerrero ML. Risk factors and clinical significance of invasive infections caused by levofloxacin-resistant Streptococcus pneumoniae. Infection. 2013;41:935–9.

88. Issler-Fisher AC, McKew G, Fisher OM, Harish V, Gottlieb T, Maitz PKM. Risk factors for, and the effect of MRSA colonization on the clinical outcomes of severely burnt patients. Burns. Elsevier Ltd and International Society of Burns Injuries; 2015;41:1212–20.

89. Ivády B, Kenesei É, Tóth-Heyn P, Kertész G, Tárkányi K, Kassa C, et al. Factors influencing antimicrobial resistance and outcome of Gram-negative bloodstream infections in children. Infection. 2016;44:309–21.

90. Jia X, Ma W, Xu X, Yang S, Zhang L. Retrospective analysis of hospital-acquired linezolid-nonsusceptible enterococci infection in Chongqing, China, 2011-2014. American Journal of Infection Control. Elsevier Inc; 2015;43:e101–6.

91. Joo E-JJ, Peck KRR, Ha YEE, Kim Y-SS, Song Y-GG, Lee S-SS, et al. Impact of acute kidney injury on mortality and medical costs in patients with meticillin-resistant Staphylococcus aureus bacteraemia: a retrospective, multicentre observational study. Journal of Hospital Infection. 2013;83:300–6.

92. Kang C-I, Song J-H, Kim SH, Chung DR, Peck KR, Thamlikitkul V, et al. Association of levofloxacin resistance with mortality in adult patients with invasive pneumococcal diseases: a post hoc analysis of a prospective cohort. Infection. 2013;41:151–7.

93. Keddy KH, Sooka A, Musekiwa A, Smith AM, Ismail H, Tau NP, et al. Clinical and microbiological features of salmonella meningitis in a South African Population, 2003-2013. Clinical Infectious Diseases. 2015;61:S272–82.

94. Kim S-H, Kwon J-C, Choi S-M, Lee D-G, Park SH, Choi J-H, et al. Escherichia coli and Klebsiella pneumoniae bacteremia in patients with neutropenic fever: factors associated with extended-spectrum β-lactamase production and its impact on outcome. Annals of Hematology. 2013;92:533–41.

95. Kim C-J, Kim H-B, Oh M, Kim YYK, Kim A, Oh S-H, et al. The burden of nosocomial staphylococcus aureus bloodstream infection in South Korea: a prospective hospital-based nationwide study. BMC infectious diseases. 2014;14:590.

96. Kim SJ, Park KH, Chung JW, Sung H, Choi SH, Choi SH. Prevalence and impact of extended-spectrum Beta-lactamase production on clinical outcomes in cancer patients with enterobacter species bacteremia. Korean Journal of Internal Medicine. 2014;29:637–46.

97. Kim MJ, Song K-H, Kim N-H, Choe PG, Park WB, Bang JH, et al. Clinical outcomes of spontaneous bacterial peritonitis due to extended-spectrum beta-lactamase-producing Escherichia coli or Klebsiella pneumoniae: a retrospective cohort study. Hepatology International. 2014;8:582–7.

98. Kim DH, Tate J, Dresen WF, Papa FC, Bloch KC, Kalams SA, et al. Cardiac implanted electronic device-related infective endocarditis: Clinical features, management, and outcomes of 80 consecutive patients. PACE - Pacing and Clinical Electrophysiology. 2014;37:978–85.

99. Kini AR, Shetty V, Kumar AM, Shetty SM, Shetty A. Community-associated, methicillin-susceptible, and methicillin-resistant Staphylococcus aureus bone and joint infections in children. Journal of Pediatric Orthopaedics B. 2013;22:158–66.

100. Kitazono H, Rog D, Sa G, Nm C, Acinetobacter RGE. Acinetobacter baumannii infection in solid organ transplant recipients. Clinical Transplantation. 2015;227–32.

101. KPMG. The global economic impact of anti-microbial resistance. 2014.

102. Krueger AL, Greene S a., Barzilay EJ, Henao O, Vugia D, Hanna S, et al. Clinical Outcomes of Nalidixic Acid, Ceftriaxone, and Multidrug-Resistant Nontyphoidal *Salmonella* Infections Compared with Pansusceptible Infections in FoodNet Sites, 2006–2008. Foodborne Pathogens and Disease. 2014;11:335–41.

103. Kumar A, Randhawa VS, Nirupam N, Rai Y, Saili A. Risk factors for carbapenem-resistant Acinetobacter baumanii blood stream infections in a neonatal intensive care unit, Delhi, India. The Journal of Infection in Developing Countries. 2014;8:2–7.

104. Lai H-H, Liou B-H, Chang Y-Y, Kuo S-C, Lee Y-T, Chen T-L, et al. Risk factors and clinical outcome of sulbactam nonsusceptibility in monomicrobial Acinetobacter nosocomialis bacteremia. Journal of Microbiology, Immunology and Infection. Elsevier Taiwan LLC; 2016;49:371–7.

105. Lanini S, Costa AN, Puro V, Procaccio F, Grossi PA, Vespasiano F, et al. Incidence of carbapenem-resistant gram negatives in Italian transplant recipients: A nationwide surveillance study. PLoS ONE. 2015;10:1–15.

106. Lee BY, Singh A, David MZ, Bartsch SM, Slayton RB, Huang SS, et al. The economic burden of community-associated methicillin-resistant Staphylococcus aureus (CA-MRSA). Clinical Microbiology and Infection. Elsevier; 2013;19:528–36.

107. Lee H-Y, Chen C-L, Wu S-R, Huang C-W, Chiu C-H. Risk factors and outcome analysis of acinetobacter baumannii complex bacteremia in critical patients. Critical care medicine. 2014;42:1081–8.

108. Lee JY, Chong YP, Kim T, Hong HL, Park SJ, Lee ES, et al. Bone and joint infection as a predictor of community-acquired methicillin-resistant Staphylococcus aureus bacteraemia: A comparative cohort study. Journal of Antimicrobial Chemotherapy. 2014;69:1966–71.

109. Lee H-Y, Chen C-L, Liu S-Y, Yan Y-S, Chang C-J, Chiu C-H. Impact of Molecular Epidemiology and Reduced Susceptibility to Glycopeptides and Daptomycin on Outcomes of Patients with Methicillin-Resistant Staphylococcus aureus Bacteremia. PLoS ONE. 2015;10:e0136171.

110. Lee HY, Huang CW, Chen CL, Wang YH, Chang CJ, Chiu CH. Emergence in Taiwan of novel imipenem-resistant Acinetobacter baumannii ST455 causing bloodstream infection in critical patients. Journal of Microbiology, Immunology and Infection. Elsevier Taiwan LLC; 2015;48:588–96.

111. Lee JK, Lee J, Park YS, Lee CH, Yim JJ, Yoo CG, et al. Clinical manifestations of pneumonia according to the causative organism in patients in the intensive care unit. Korean Journal of Internal Medicine. 2015;30:829–36.

112. Leistner R, Sakellariou C, Gürntke S, Kola A, Steinmetz I, Kohler C, et al. Mortality and molecular epidemiology associated with extended-spectrum β-lactamase production in Escherichia coli from bloodstream infection. Infection and Drug Resistance. 2014;7:57–62.

113. Leistner R, Gürntke S, Sakellariou C, Denkel LA, Bloch A, Gastmeier P, et al. Bloodstream infection due to extended-spectrum beta-lactamase (ESBL)-positive K. pneumoniae and E. coli: an analysis of the disease burden in a large cohort. Infection. 2014;42:991–7.

114. Leistner R, Bloch A, Sakellariou C, Gastmeier P, Schwab F. Costs and length of stay associated with extended-spectrum β-lactamase production in cases of Escherichia coli bloodstream infection. Journal of Global Antimicrobial Resistance. Taibah University; 2014;2:107–9.

115. Lemos E V., de la Hoz FP, Alvis N, Einarson TR, Quevedo E, Castañeda C, et al. Impact of carbapenem resistance on clinical and economic outcomes among patients with Acinetobacter baumannii infection in Colombia. Clinical Microbiology and Infection. 2014;20:174–80.

116. Liao L, Xing H, Su B, Wang Z, Ruan Y, Wang X, et al. Impact of HIV drug resistance on virologic and immunologic failure and mortality in a cohort of patients on antiretroviral therapy in China. Aids. 2013;27:1815–24.

117. Lichtenfels E, D’Azevedo P., Frankini A., Erling N, Aerts N. Morbidity and mortality associated with arterial surgery site infections by resistant microorganisms. Jornal Vascular Brasileiro. 2014;13:175–81.

118. Liew Y-X, Tan T-T, Lee W, Ng J-L, Chia D-Q, Wong G-C, et al. Risk factors for extreme-drug resistant Pseudomonas aeruginosa infections in patients with hematologic malignancies. American Journal of Infection Control. Elsevier Inc; 2013;41:140–4.

119. Lin K-Y, Lauderdale T-L, Wang J-T, Chang S-C. Carbapenem-resistant Pseudomonas aeruginosa in Taiwan: Prevalence, risk factors, and impact on outcome of infections. Journal of microbiology, immunology, and infection = Wei mian yu gan ran za zhi. Elsevier Taiwan LLC; 2014;49:1–8.

120. Lin W-T, Wu C-D, Cheng S-C, Chiu C-C, Tseng C-C, Chan H-T, et al. High Prevalence of Methicillin-Resistant Staphylococcus aureus among Patients with Septic Arthritis Caused by Staphylococcus aureus. PloS one. 2015;10:e0127150.

121. Lloyd-Smith P, Younger J, Lloyd-Smith E, Green H, Leung V, Romney MG. Economic analysis of vancomycin-resistant enterococci at a Canadian hospital: assessing attributable cost and length of stay. Journal of Hospital Infection. Elsevier Ltd; 2013;85:54–9.

122. Lübbert C, Becker-Rux D, Rodloff AC, Laudi S, Busch T, Bartels M, et al. Colonization of liver transplant recipients with KPC-producing Klebsiella pneumoniae is associated with high infection rates and excess mortality: A case-control analysis. Infection. 2014;42:309–16.

123. Lubell Y, Dondorp A, Guérin PJ, Drake T, Meek S, Ashley E, et al. Artemisinin resistance – modelling the potential human and economic costs. Malaria Journal. 2014;13:452.

124. Lucena A, Dalla Costa LM, Nogueira KS, Matos AP, Gales AC, Paganini MC, et al. Nosocomial infections with metallo-beta-lactamase-producing Pseudomonas aeruginosa: Molecular epidemiology, risk factors, clinical features and outcomes. Journal of Hospital Infection. Elsevier Ltd; 2014;87:234–40.

125. Macedo-Viñas M, De Angelis G, Rohner P, Safran E, Stewardson a., Fankhauser C, et al. Burden of meticillin-resistant Staphylococcus aureus infections at a Swiss University hospital: excess length of stay and costs. Journal of Hospital Infection. 2013;84:132–7.

126. MacVane SH, Tuttle LO, Nicolau DP. Impact of extended-spectrum β-lactamase-producing organisms on clinical and economic outcomes in patients with urinary tract infection. Journal of Hospital Medicine. 2014;9:232–8.

127. Manandhar S, Pai G, Gidwani H, Nazim S, Buehrle D, Shutt KA, et al. Does Staphylococcus aureus Bacteriuria Predict Clinical Outcomes in Patients With Bacteremia? Analysis of 274 Patients With Staphylococcus aureus Blood Stream Infection. Infectious Diseases in Clinical Practice. 2016;24:151–4.

128. Maor Y, Belausov N, Ben-David D, Smollan G, Keller N, Rahav G. hVISA and MRSA endocarditis: An 8-year experience in a tertiary care centre. Clinical Microbiology and Infection. 2013;20:O730–6.

129. Marín M, Gudiol C, Garcia-Vidal C, Ardanuy C, Carratalà J. Bloodstream Infections in Patients With Solid Tumors. Medicine. 2014;93:143–9.

130. Martelius T, Jalava J, Kärki T, Möttönen T, Ollgren J, Lyytikäinen O. Nosocomial bloodstream infections caused by Escherichia coli and Klebsiella pneumoniae resistant to third-generation cephalosporins, Finland, 1999-2013: Trends, patient characteristics and mortality. Infectious Diseases. 2016;48:229–34.

131. Marwick C, Santiago VH, McCowan C, Broomhall J, Davey P. Community acquired infections in older patients admitted to hospital from care homes versus the community: cohort study of microbiology and outcomes. BMC geriatrics. 2013;13:12.

132. Melzer M, Welch C. Outcomes in UK patients with hospital-acquired bacteraemia and the risk of catheter-associated urinary tract infections. Postgraduate medical journal. 2013;89:329–34.

133. Micek ST, Wunderink RG, Kollef MH, Chen C, Rello J, Chastre J, et al. An international multicenter retrospective study of Pseudomonas aeruginosa nosocomial pneumonia: Impact of multidrug resistance. Critical care (London, England). 2015;19:219.

134. Micek ST, Kollef MH, Torres A, Chen C, Rello J, Chastre J, et al. Pseudomonas aeruginosa Nosocomial Pneumonia: Impact of Pneumonia Classification. Infection Control & Hospital Epidemiology. 2015;36:1190–7.

135. Miles-Jay A, Butler-Wu S, Rowhani-Rahbar A, Pergam SA. Incidence rate of fluoroquinolone resistant gram-negative rod bacteremia among allogeneic hematopoietic cell transplant patients during an era of levofloxacin prophylaxis. Biol Blood Marrow Transplant. 2015;257:539–45.

136. Miller TTL, Cirule A, Wilson F a FF a, Holtz TH, Riekstina V, Cain KP, et al. The value of effective public tuberculosis treatment: an analysis of opportunity costs associated with multidrug resistant tuberculosis in Latvia. Cost effectiveness and resource allocation : C/E. 2013;11:9.

137. Nelson RE, Samore MH, Jones M, Greene T, Stevens VW, Liu CF, et al. Reducing Time-dependent Bias in Estimates of the Attributable Cost of Health Care-associated Methicillin-resistant Staphylococcus aureus Infections: A Comparison of Three Estimation Strategies. Med Care. 2015;53:827–34.

138. Nelson RE, Jones M, Liu C-F, Samore MH, Evans ME, Graves N, et al. The Impact of Healthcare-Associated Methicillin-Resistant Staphylococcus Aureus Infections on Post-Discharge Healthcare Costs and Utilization. Infection Control & Hospital Epidemiology. 2015;36:534–542 9p.

139. Nelson RE, Stevens VW, Jones M, Samore MH, Rubin MA. Health care associated methicillin-resistant Staphylococcus aureus infections increases the risk of postdischarge mortality. American Journal of Infection Control. Elsevier Inc; 2015;43:38–43.

140. Ny P, Nieberg P, Wong-Beringer A. Impact of carbapenem resistance on epidemiology and outcomes of nonbacteremic Klebsiella pneumoniae infections This work was presented in part at the Interscience Conference on Antimicrobial Agents and Chemotherapy, Washington, DC, September 7, 2014 (post. American Journal of Infection Control. Elsevier Inc; 2015;43:1076–80.

141. Nitta H, Beppu T, Itoyama A, Higashi T, Sakamoto K, Nakagawa S, et al. Poor outcomes after hepatectomy in patients with ascites infected by methicillin-resistant staphylococci. Journal of Hepatobiliary-Pancreatic Sciences. 2015;22:166–76.

142. Nogueira KS, Paganini MC, Conte A, Cogo L, da Silva M, Taborda de Messias Reason I, et al. Emergence of extended-spectrum Beta-lactamase producing Enterobacter spp. in patients with bacteremia in a tertiary hospital in southern Brazil. Enferm Infecc Microbiol Clin. 2014;32:87–92.

143. Nouvenne A, Ticinesi A, Lauretani F, Maggio M, Lippi G, Guida L, et al. Comorbidities and disease severity as risk factors for Carbapenem-Resistant klebsiella pneumoniae colonization: Report of an experience in an internal medicine unit. PLoS ONE. 2014;9:1–8.

144. O’Donnell MR, Pillay M, Pillay M, Werner L, Master I, Wolf A, et al. Primary capreomycin resistance is common and associated with early mortality in patients with extensively drug-resistant tuberculosis in KwaZulu-Natal, South Africa. JAIDS, Journal of Acquired Immune Deficiency Syndromes. 2015;69:536–43.

145. Okada Y, Hosono M, Sasaki Y, Hirai H, Suehiro S. Preoperative increasing C-reactive protein affects the outcome for active infective endocarditis. Annals of Thoracic and Cardiovascular Surgery. 2014;20:48–54.

146. Oliveira MC, Oliveira CRA, Gonçalves KV, Santos MS, Tardelli ACS, Nobre VA. Enterobacteriaceae resistant to third generation cephalosporins upon hospital admission: Risk factors and clinical outcomes. Brazilian Journal of Infectious Diseases. 2015;19:239–45.

147. Ong SJ, Huang Y-C, Tan H-Y, Ma DHK, Lin H-C, Yeh L-K, et al. Staphylococcus aureus Keratitis: A Review of Hospital Cases. PLoS ONE. 2013;8:e80119.

148. Ong CW, Hons M, Rn JLR, Nursing B, Collignon PJ. Long-term survival outcome following Staphylococcus aureus bacteraemia. 2013;102–9.

149. Orsi GB, Giuliano S, Franchi C, Ciorba V, Protano C, Giordano A, et al. Changed epidemiology of ICU acquired bloodstream infections over 12 years in an Italian teaching hospital. Minerva Anestesiologica. 2015;81:980–8.

150. Ostermann H, Blasi, Medina J, Pascual E, McBride K, Garau J. Resource use in patients hospitalized with complicated skin and soft tissue infections in Europe and analysis of vulnerable groups : the REACH study. J Med Econ. 2014;17:719–29.

151. Osthoff M, McGuinness SL, Wagen AZ, Eisen DP. Urinary tract infections due to extended-spectrum beta-lactamase-producing Gram-negative bacteria: identification of risk factors and outcome predictors in an Australian tertiary referral hospital. International journal of infectious diseases : IJID : official publication of the International Society for Infectious Diseases. International Society for Infectious Diseases; 2015;34:79–83.

152. Özgür ES, Horasan ES, Karaca K, Ersöz G, Naycı Atış S, Kaya A. Ventilator-associated pneumonia due to extensive drug-resistant Acinetobacter baumannii: Risk factors, clinical features, and outcomes. American Journal of Infection Control. 2014;42:206–8.

153. Papadimitriou-Olivgeris M, Marangos M, Fligou F, Christofidou M, Sklavou C, Vamvakopoulou S, et al. KPC-producing Klebsiella pneumoniae enteric colonization acquired during intensive care unit stay: the significance of risk factors for its development and its impact on mortality. Diagnostic Microbiology and Infectious Disease. 2013;77:169–73.

154. Park SY, Oh IH, Lee HJ, Ihm CG, Son JS, Lee MS, et al. Impact of reduced vancomycin MIC on clinical outcomes of methicillin-resistant Staphylococcus aureus Bacteremia. Antimicrobial Agents and Chemotherapy. 2013;57:5536–42.

155. Park K-H, Shin J-H, Lee SY, Kim SH, Jang MO, Kang S-J, et al. The Clinical Characteristics, Carbapenem Resistance, and Outcome of Acinetobacter Bacteremia According to Genospecies. PLoS ONE. 2013;8:e65026.

156. Park SY, Choo JW, Kwon SH, Yu SN, Lee EJ, Kim TH, et al. Risk Factors for Mortality in Patients with Acinetobacter baumannii Bacteremia. Infection & chemotherapy. 2013;45:325–30.

157. Park K-H, Chong YP, Kim S-H, Lee S-O, Choi S-H, Lee MS, et al. Community-associated MRSA strain ST72-SCCmecIV causing bloodstream infections: clinical outcomes and bacterial virulence factors. Journal of Antimicrobial Chemotherapy. 2014;1185–92.

158. Patel SJ, Oliveira AP, Zhou JJ, Alba L, Furuya EY, Weisenberg SA, et al. Risk factors and outcomes of infections caused by extremely drug-resistant gram-negative bacilli in patients hospitalized in intensive care units. American Journal of Infection Control. 2014;42:626–31.

159. Patel TS, Nagel JL. Clinical outcomes of enterobacteriaceae infections stratified by carbapenem MICs. Journal of Clinical Microbiology. 2015;53:201–5.

160. Pau CKY, Ma FFT, Ip M, You JHS. Characteristics and outcomes of *Klebsiella pneumoniae* bacteraemia in Hong Kong. Infectious Diseases. 2015;47:283–8.

161. Peirovifar A, Ahangarzadeh Rezaee M, Mostafa Gharehbaghi M. Prevalence of Multidrug Resistant Extended-SpectrumBeta-Lactamase Producing Gram-Negative Bacteria inNeonatal Sepsis. International Journal of Women’s Health and Reproduction Sciences. 2014;2:138–45.

162. Peng Y, Bi J, Shi J, Li Y, Ye X, Chen X, et al. Multidrug-resistant Pseudomonas aeruginosa infections pose growing threat to health careeassociated infection control in the hospitals of Southern China: A case-control surveillance study. American Journal of Infection Control. Elsevier Inc; 2014;42:1308–11.

163. Pereira MR, Scully BF, Pouch SM, Uhlemann A-C, Goudie S, Emond JE, et al. Risk factors and outcomes of carbapenem-resistant Klebsiella pneumoniae infections in liver transplant recipients. Liver Transplantation. 2015;21:1511–9.

164. Pooran A, Pieterson E, Davids M, Theron G, Dheda K. What is the Cost of Diagnosis and Management of Drug Resistant Tuberculosis in South Africa? PLoS ONE. 2013;8:e54587.

165. Porto JP, Santos RO, Filho PPG, Ribas RM. Active surveillance to determine the impact of methicillin resistance on mortality in patients with bacteremia and influences of the use of antibiotics on the development of MRSA infection. Revista da Sociedade Brasileira de Medicina Tropical. 2013;46:713–8.

166. Post FA, Grint D, Werlinrud AM, Panteleev A, Riekstina V, Malashenkov EA, et al. Multi-drug-resistant tuberculosis in HIV positive patients in Eastern Europe. The Journal of infection. 2014;68:259–63.

167. Pouch SM, Kubin CJ, Satlin MJ, Tsapepas DS, Lee JR, Dube G, et al. Epidemiology and outcomes of carbapenem-resistant Klebsiella pneumoniae bacteriuria in kidney transplant recipients. Transplant Infectious Disease. 2015;17:800–9.

168. Prasad KN, Singh K, Rizwan a., Mishra P, Tiwari D, Prasad N, et al. Microbiology and Outcomes of Peritonitis in Northern India. Peritoneal Dialysis International. 2014;34:188–94.

169. Prina E, Ranzani OT, Polverino E, Cill??niz C, Ferrer M, Fernandez L, et al. Risk factors associated with potentially antibiotic-resistant pathogens in community-acquired pneumonia. Annals of the American Thoracic Society. 2015;12:153–60.

170. Puig-Asensio M, Ruiz-Camps I, Fernández-Ruiz M, Aguado JM, Muñoz P, Valerio M, et al. Epidemiology and outcome of candidaemia in patients with oncological and haematological malignancies: RESULTS from a population-based surveillance in Spain. Clinical Microbiology and Infection. Elsevier Ltd; 2015;21:491.e1-491.e10.

171. Qureshi Z a, Syed A, Clarke LG, Doi Y, Shields RK. Epidemiology and clinical outcomes of patients with carbapenem-resistant Klebsiella pneumoniae bacteriuria. Antimicrobial agents and chemotherapy. 2014;58:1–18.

172. Rello J, Molano D, Villabon M, Reina R, Rita-Quispe R, Previgliano I, et al. Differences in hospital- and ventilator-associated pneumonia due to Staphylococcus aureus (methicillin-susceptible and methicillin-resistant) between Europe and Latin America: a comparison of the EUVAP and LATINVAP study cohorts. Medicina intensiva / Sociedad Española de Medicina Intensiva y Unidades Coronarias. 2013;37:241–7.

173. Reynolds C a, Finkelstein J a, Ray GT, Moore MR, Huang SS. Attributable healthcare utilization and cost of pneumonia due to drug-resistant streptococcus pneumonia: a cost analysis. Antimicrobial resistance and infection control. 2014;3:16.

174. Rieg S, Jonas D, Kaasch AJ, Porzelius C, Peyerl-Hoffmann G, Theilacker C, et al. Microarray-Based Genotyping and Clinical Outcomes of Staphylococcus aureus Bloodstream Infection: An Exploratory Study. PLoS ONE. 2013;8:e71259.

175. Rodríguez-Baño J, Mingorance J, Fernández-Romero N, Serrano L, López-Cerero L, Pascual A. Outcome of bacteraemia due to extended-spectrum β-lactamase-producing Escherichia coli: Impact of microbiological determinants. Journal of Infection. 2013;67:27–34.

176. Rose HR, Holzman RS, Altman DR, Smyth DS, Wasserman GA, Kafer JM, et al. Cytotoxic virulence predicts mortality in nosocomial pneumonia due to methicillin-resistant Staphylococcus aureus. Journal of Infectious Diseases. 2015;211:1862–74.

177. Sakoda Y, Ikegame S, Ikeda-Harada C, Takakura K, Kumazoe H, Wakamatsu K, et al. Retrospective analysis of nursing and healthcare-associated pneumonia: Analysis of adverse prognostic factors and validity of the selection criteria. Respiratory Investigation. Elsevier; 2014;52:114–20.

178. Samonis G, Vardakas KZ, Kofteridis DP, Dimopoulou D, Andrianaki AM, Chatzinikolaou I, et al. Characteristics, risk factors and outcomes of adult cancer patients with extensively drug-resistant Pseudomonas aeruginosa infections. Infection. 2014;42:721–8.

179. Saravu K, Preethi V, Kumar R, Guddattu V, Shastry AB, Mukhopadhyay C. Determinants of ventilator associated pneumonia and its impact on prognosis: A tertiary care experience. Indian J Crit Care Med. 2013;17:337–42.

180. Seboxa T, Amogne W, Abebe W, Tsegaye T, Azazh A, Hailu W, et al. High mortality from blood stream infection in Addis Ababa, Ethiopia, is due to antimicrobial resistance. PLoS ONE. 2015;10:1–14.

181. Shorr AF, Zilberberg MD, Micek ST, Kollef MH. Outcomes associated with bacteremia in the setting of methicillin-resistant Staphylococcus aureus pneumonia: a retrospective cohort study. Critical Care. Critical Care; 2015;19:312.

182. Shuldiner J, Leventhal a., Chemtob D, Mor Z. Mortality of tuberculosis patients during treatment in israel, 2000-2010. International Journal of Tuberculosis and Lung Disease. 2014;18:818–23.

183. Simkins J, Muggia V, Cohen HW, Minamoto GY. Carbapenem-resistant *Klebsiella pneumoniae* infections in kidney transplant recipients: a case-control study. Transplant Infectious Disease. 2014;16:775–82.

184. Smith KD, Achan B, Hullsiek KH, Mcdonald TR, Okagaki LH, Alhadab AA, et al. Increased Antifungal Drug Resistance in Clinical Isolates of Cryptococcus neoformans in Uganda. Antimicrobial Agents and Chemotherapy. 2015;59:7197–204.

185. Smithson A, Ramos J, Bastida MT, Bernal S, Jove N, Ni??o E, et al. Differential characteristics of healthcare-associated compared to community-acquired febrile urinary tract infections in males. European Journal of Clinical Microbiology and Infectious Diseases. 2015;34:2395–402.

186. Souza ES, Belei RA, Carrilho CMD de M, Matsuo T, Yamada-Ogatta SF, Andrade G, et al. Mortality and risks related to healthcare-associated infection. Texto & Contexto - Enfermagem. 2015;24:220–8.

187. Sozen H, Caylak S, Cetinkaya M, Citil BE, Sahins C, Deliktas H, et al. Clinical and Economic Outcomes Associated with Urinary Tract Infections Caused by Extended Spectrum Beta-lactamase Producing Bacteria in a Tertiary Care Hospital. Studies on Ethno-Medicine. 2015;9:173–9.

188. Stewardson A, Fankhauser C, De Angelis G, Rohner P, Safran E, Schrenzel J, et al. Burden of bloodstream infection caused by extended-spectrum β-lactamase-producing enterobacteriaceae determined using multistate modeling at a Swiss University Hospital and a nationwide predictive model. Infection control and hospital epidemiology : the official journal of the Society of Hospital Epidemiologists of America. 2013;34:133–43.

189. Sulla F, Bussius D., Acquesta F, Navarini A, Sasagawa S., Mimica M. Vancomycin minimum inhibitory concentrations and lethality in Staphylococcus aureus bacteremia. Germs. 2015;5:39–43.

190. Tadros M, Williams V, Coleman BL, McGeer AJ, Haider S, Lee C, et al. Epidemiology and Outcome of Pneumonia Caused by Methicillin-Resistant Staphylococcus aureus (MRSA) in Canadian Hospitals. PLoS ONE. 2013;8:4–11.

191. Takata T, Miyazaki M, Futo M, Hara S, Shiotsuka S, Kamimura H, et al. Presence of both heterogeneous vancomycin-intermediate resistance and β-lactam antibiotic-induced vancomycin resistance phenotypes is associated with the outcome in methicillin-resistant Staphylococcus aureus bloodstream infection. Scandinavian Journal of Infectious Diseases. 2013;45:203–12.

192. Tavadze M, Rybicki L, Mossad S, Avery R, Yurch M, Pohlman B, et al. Risk factors for vancomycin-resistant enterococcus bacteremia and its influence on survival after allogeneic hematopoietic cell transplantation. Bone marrow transplantation. Nature Publishing Group; 2014;49:1310–6.

193. Taylor J, Hafner M, Yerushalmi E, Smith R, Bellasio J, Vardavas R, et al. Estimating the economic costs of antimicrobial resistance. 2014.

194. Tedja R, Nowacki A, Fraser T, Fatica C, Griffiths L, Gordon S, et al. The impact of multidrug resistance on outcomes in ventilator-associated pneumonia. American Journal of Infection Control. 2014;42:542–5.

195. Teillant A, Gandra S, Barter D, Morgan DJ, Laxminarayan R. Potential burden of antibiotic resistance on surgery and cancer chemotherapy antibiotic prophylaxis in the USA: A literature review and modelling study. The Lancet Infectious Diseases. Elsevier Ltd; 2015;15:1429–37.

196. Thampi N, Showler A, Burry L, Bai AD, Steinberg M, Ricciuto DR, et al. Multicenter study of health care cost of patients admitted to hospital with Staphylococcus aureus bacteremia: Impact of length of stay and intensity of care. American Journal of Infection Control. Elsevier Inc; 2015;43:739–44.

197. Theodorou P, Thamm O., Perbix W, Phan V. Pseudomonas aeruginosa bacteremia after burn injury: The impact of multiple-drug resistance. J Burn Care Res. 2013;34:649–58.

198. Theodorou P, Lefering R, Perbix W, Spanholtz T a., Maegele M, Spilker G, et al. Staphylococcus aureus bacteremia after thermal injury: The clinical impact of methicillin resistance. Burns. Elsevier Ltd and International Society of Burns Injuries; 2013;39:404–12.

199. Tumbarello M, De Pascale G, Trecarichi EM, Spanu T, Antonicelli F, Maviglia R, et al. Clinical outcomes of Pseudomonas aeruginosa pneumonia in intensive care unit patients. Intensive Care Medicine. 2013;39:682–92.

200. Tumbarello M, Trecarichi EM, De Rosa FG, Giannella M, Giacobbe DR, Bassetti M, et al. Infections caused by KPC-producing Klebsiella pneumoniae: Differences in therapy and mortality in a multicentre study. Journal of Antimicrobial Chemotherapy. 2014;70:2133–43.

201. Van Hal SJ, Chen SCA, Sorrell TC, Ellis DH, Slavin M, Marriott DM. Support for the EUCAST and revised CLSI fluconazole clinical breakpoints by Sensititre® YeastOne® for Candida albicans: A prospective observational cohort study. Journal of Antimicrobial Chemotherapy. 2014;69:2210–4.

202. Vardakas KZ, Matthaiou DK, Falagas ME, Antypa E, Koteli A, Antoniadou E. Characteristics, risk factors and outcomes of carbapenem-resistant Klebsiella pneumoniae infections in the intensive care unit. Journal of Infection. Elsevier Ltd; 2015;70:592–9.

203. Vasudevan A, Chuang L, Jialiang L, Mukhopadhyay A, Goh EY-Y, Tambyah P a. Inappropriate empirical antimicrobial therapy for multidrug-resistant organisms in critically ill patients with pneumonia is not an independent risk factor for mortality: Results of a prospective observational study of 758 patients. Journal of Global Antimicrobial Resistance. Taibah University; 2013;1:123–30.

204. Vasudevan A, Memon B, Mukhopadhyay A, Li J, Tambyah P. The costs of nosocomial resistant gram negative intensive care unit infections among patients with the systemic inflammatory response syndrome- a propensity matched case control study. Antimicrobial Resistance and Infection Control. 2015;4:3.

205. Vazirani J, Wurity S, Ali MH. Multidrug-resistant Pseudomonas aeruginosa keratitis: Risk factors, clinical characteristics, and outcomes. Ophthalmology. Elsevier Inc; 2015;122:2110–4.

206. Vitkauskiene A, Dambrauskiene A, Cerniauskiene K, Rimdeika R, Sakalauskas R. Risk factors and outcomes in patients with carbapenem-resistant Acinetobacter infection. Scandinavian Journal of Infectious Diseases. 2013;45:213–8.

207. Wang JL, Lai CH, Lin HH, Chen WF, Shih YC, Hung CH. High vancomycin minimum inhibitory concentrations with heteroresistant vancomycin-intermediate Staphylococcus aureus in meticillin-resistant S. aureus bacteraemia patients. International Journal of Antimicrobial Agents. Elsevier B.V.; 2013;42:390–4.

208. Wang T-Y, Lin S-M, Shie S-S, Chou P-C, Huang C-D, Al. E. Clinical Characteristics and Treatment Outcomes of Patients with Isoniazid-Monoresistant Tuberculosis. PLoS ONE. 2014;9.

209. Wang E, Farmakiotis D, Yang D, Mccue DA, Kantarjian HM, Kontoyiannis DP, et al. The ever-evolving landscape of candidaemia in patients with acute leukaemia: Non-susceptibility to caspofungin and multidrug resistance are associated with increased mortality. Journal of Antimicrobial Chemotherapy. 2015;70:2362–8.

210. Wang L, Wang Y, Fan X, Tang W, Hu J. Prevalence of Resistant Gram-Negative Bacilli in Bloodstream Infection in Febrile Neutropenia Patients Undergoing Hematopoietic Stem Cell Transplantation. Medicine. 2015;94:e1931.

211. Wenzler E, Goff DA, Bazan JA, Bauer KA. Clinical Outcomes in Patients With Ceftriaxone-Resistant Streptococcus pneumoniae Pneumonia. Infectious Diseases in clinical practice. 2014;22:263–6.

212. Willmann M, Kuebart I, Marschal M, Schröppel K, Vogel W, Flesch I, et al. Effect of metallo-β-lactamase production and multidrug resistance on clinical outcomes in patients with Pseudomonas aeruginosa bloodstream infection: a retrospective cohort study. BMC Infectious Diseases. 2013;13:515.

213. Wingfield T, Boccia D, Tovar M, Gavino A, Zevallos K, Montoya R, et al. Defining Catastrophic Costs and Comparing Their Importance for Adverse Tuberculosis Outcome with Multi-Drug Resistance: A Prospective Cohort Study, Peru. PLoS Medicine. 2014;11:e1001675.

214. Wu YH, Chen PL, Hung YP, Ko WC. Risk factors and clinical impact of levofloxacin or cefazolin nonsusceptibility or ESBL production among uropathogens in adults with community-onset urinary tract infections. Journal of Microbiology, Immunology and Infection. Elsevier Taiwan LLC; 2014;47:197–203.

215. Yao Z, Peng Y, Chen X, Bi J, Li Y, Ye X, et al. Healthcare associated infections of Methicillin-resistant Staphylococcus aureus: A case-control-control study. PLoS ONE. 2015;10:1–9.

216. Yavuz SŞ, Şensoy A, Çeken S, Deniz D, Yekeler I. Methicillin-resistant Staphylococcus aureus infection: An independent risk factor for mortality in patients with poststernotomy mediastinitis. Medical Principles and Practice. 2014;23:517–23.

217. Zhang HT, Liu H. Laboratory-based evaluation of MDR strains of Pseudomonas in patients with acute burn injuries. International Journal of Clinical and Experimental Medicine. 2015;8:16512–9.

218. Zulqarnain A, Maqbool B, Iqbal I. Comparison of Duration of Hospitalization and Clincial Outcome in Children Infected with Methicillin Resistant Staphylococcus Aureus and Methicillin Sensitive Staphylococcus Aureus. Med. Forum. 2014;25:21–3.
